# Norcantharidin ameliorates estrogen deficient-mediated bone loss by attenuating the activation of extracellular signal-regulated kinase/ROS/NLRP3 inflammasome signaling

**DOI:** 10.3389/fphar.2022.1019478

**Published:** 2022-11-03

**Authors:** Guang Yang, Huikang Xu, Minjun Yao, Shigui Yan, Mengrui Wu, Chenhe Zhou

**Affiliations:** ^1^ Department of Orthopedic Surgery, The Second Affiliated Hospital, Zhejiang University School of Medicine, Hangzhou City, Zhejiang, China; ^2^ Orthopedics Research Institute of Zhejiang University, Hangzhou City, Zhejiang, China; ^3^ Key Laboratory of Motor System Disease Research and Precision Therapy of Zhejiang Province, Hangzhou City, Zhejiang, China; ^4^ Clinical Research Center of Motor System Disease of Zhejiang Province, Hangzhou City, China; ^5^ State Key Laboratory for Diagnosis and Treatment of Infectious Diseases, National Clinical Research Center for Infectious Diseases, Collaborative Innovation Center for Diagnosis and Treatment of Infectious Diseases, The First Affiliated Hospital, College of Medicine, Zhejiang University, Hangzhou, China; ^6^ Department of Cell and Developmental Biology, College of Life Sciences, Zhejiang University, Hangzhou, China

**Keywords:** osteoclast, osteoporosis, norcantharidin, ERK, ROS, NLRP3 inflammasome

## Abstract

Osteoporosis, characterized by reduced bone mass, aberrant bone architecture, and elevated bone fragility, is driven by a disruption of bone homeostasis between bone resorption and bone formation. However, up to now, no drugs are perfect for osteoporosis treatment due to different defects. In this study, we demonstrated that norcantharidin (NCTD) could inhibit osteoclast formation and bone resorption by attenuating the ERK, ROS and NLRP3 inflammasomes pathways *in vitro*. Moreover, our *in vivo* study further confirms its preventive effects on estrogen-deficiency bone loss by inhibiting osteoclast formation and functions. Therefore, we could conclude that NCTD might be a potential candidates for the prevention and treatment of osteoporosis.

## Introduction

Osteoporosis, characterized by reduced bone mass, aberrant bone architecture, and elevated bone fragility, is driven by a disruption of bone homeostasis between bone resorption and bone formation (Ivaska et al., 2010). About 34% of females above 50 years old were suffering from osteoporosis worldwide, and two million fractures were reported related to osteoporosis in the United States, accounting for over 19 billion dollars annual health costs (Burge et al., 2007; Wright et al., 2014). Estrogen deficiency and age are considered to be the two major causes of osteoporosis, leading to the overactivation of osteoclasts and relatively weak osteogenesis (Rosen, 2000). Up to now, various strategies aiming at regulating abnormal bone metabolism have been utilized to restore bone mass clinically, including anabolic reagents and anti-resorptive reagents. However, some concerns limit long-time clinical use, such as the increased osteonecrosis of the jaw after bisphosphonates treatment, and the high cost of teriparatide treatment (Pfister et al., 2006; Ferreira et al., 2021). Recent years, a variety of compounds were extracted or synthesized to explore their effects on bone cells and osteoporosis, and some have been confirmed effective (Chen et al., 2018). However, issues were found in most of these agents preclinically, such as the cardiovascular adverse effects of odanacatib (Drake et al., 2017). Therefore, screening a potential drug is urgent demands for osteoporosis treatment.

Multiple anti-osteoporotic medications have been widely used in clinic, including anabolic drugs and anti-catabolic drugs, aiming at directly or secondary improving bone mass (Bernabei et al., 2014). However, up to now, no drugs are perfect for osteoporosis treatment due to different defects. For example, bisphosphonates, a first-line anti-osteoporotic drug, is proven to induce side-effects such as osteonecrosis of the jaw and atypical fractures. Teriparatide, a classical anabolic drug, is not allowed over 24 months treatment duration during lifetime. Therefore, it is urgent to find out an effective drug with few side-effects for osteoporosis therapy.

Recent years, increased number of new compounds have been sought and verified effective on improving bone quality. Norcantharidin (NCTD), a demethylated form of cantharidin, was used as a adjuvant drug for cancer treatment and immune enhancement for decades years (Wang G. et al., 2018; Zhou et al., 2020). Besides, NCTD also exhibits various other pharmacological activities, such as benefical effects on renal tubulointerstitial fibrosis, antioxidant effect (Li et al., 2012; Zhou et al., 2020). However, to date, how NCTD affects bone metabolism is still unclear. Huang et al. (2020), has found that a composite containing NCTD could promote osteogenesis *in vitro*, but the exact mechanism is still unknown. As NCTD has demonstrated inhibitory effects on multiple signaling pathways *in vitro*, such as the ERK, AKT, JNK signaling, which are crucial in osteoclastogenesis (Zhou et al., 2018a). Therefore, we hypothesize that NCTD might exhibit a suppressive effect on osteoclast formation and functions, subsequently regulating bone metabolism. In this study, we explored the effect of NCTD on osteoclast formation and function, and further investigated its underlying mechanism.

## Methods and reagents

### Media and reagents

NCTD, obtain from Selleck Chemicals (Houston, United States) and dissolved in DMSO. Alpha modification of Eagle’s medium (a-MEM) and penicillin/streptomycin were gained from Boster Bio (Wuhan, China), while fetal bovine serum (FBS) was purchased from Gibco-BRL (Sydney, Australia). Primary antibodies against ERK, JNK, p38, phosphorylated p-ERK, p-JNK, p-p38, IkBa, p-IkBa, AKT, p-AKT, NOD-like receptor thermal protein domain associated protein 3 (NLRP3), apoptosis-associated speck-like protein containing CARD (ASC) and GAPDH were supplied from Cell Signaling Technology (Cambridge, MA, United States); while primary antibodies specific for NFATc1 and Cathepsin K were obtained from Santa Cruz Biotechnology (Santa Cruz, CA, United States).

### Extraction of bone marrow-derived macrophages and osteoclast differentiation

Bone marrow cells were obtained from C57BL/6 mice aged 3–6 weeks, and the method was referred to the previous report (Davis, 2013). Briefly, sterilized instruments, PBS, cell culture dishes, complete medium and cytokines such as M-CSF (R&D, United States) and receptor activator of nuclear factor-kappa B ligand (RANKL, R&D, United States) had been prepared in advance. After the mice were euthanized, the bone of the lower limbs were separated, and the tibia and femurs were collected. All the cells in the bone marrow were collected along the center of the bone and centrifuged for 5 min. After the erythrocytes were lysed and cleared, the remaining cells were cultured in Petri dishes containing the α-MEM complete medium. In the process of cell culture in bone marrow, 20 ng/ml M-CSF was added to the complete culture medium. After 2–3 days, adherent cells in the dish were bone marrow derived-macrophage (BMM).

After digesting BMM with 0.05% trypsin-EDTA for 5min, cells were counted by cell counter and finally seeded on 12-well, 24-well, or 96-well plates. The formation of osteoclasts could be observed under a light microscope 5–6 days after the addition of 10 ng/ml M-CSF and 15 ng/ml RANKL to cells cultured in a complete medium.

### Fluorescence quantitative polymerase chain reaction

Following the instructions, RNeasy kits (Qiagen, German) were used to extract total RNA from cells. After obtaining and quantifying RNA, reverse transcription was performed using the Hifair^®^ Ⅲ first Strand cDNA Synthesis SuperMix kit (YEASEN, China). After cDNA was obtained, the relative quantification of the target gene in cDNA can be completed with the Hieff^®^ qPCR SYBR Green Master Mix kit (YEASEN, China). GAPDH was used as an internal reference for quantitative calculation. All procedures were performed on Bio-Rad CFX96 Connect and Roche 480Ⅱ instrument. The primer sequences required for quantitative PCR were presented as follows: GAPDH, forward 5′-ACC​CAG​AAG​ACT​GTG​GAT​GG-3′ and reverse 5′-CAC​ATT​GGG​TAG​GAA​CAC-3′; Cathepsin K, forward 5′-CTT​CCA​ATA​CGT​GCA​GCA​GA-3′ and reverse 5′-TCT​TCA​GGG​CTT​TCT​CGT​TC-3′; CTR, forward 5′-TGC​AGA​CAA​CTC​TTG​GTT​GG-3′ and reverse 5′-TCG​GTT​TCT​TCT​CCT​CTG​GA-3′; TRAP, forward 5′-CTG​GAG​TGC​ACG​ATG​CCA​GCG​ACA-3′ and reverse 5′-TCC​GTG​CTC​GGC​GAT​GGA​CCA​GA-3′; NFATc1, forward 5′-CCG​TTG​CTT​CCA​GAA​AAT​AAC​A-3′ and reverse 5′-TGT​GGG​ATG​TGA​ACT​CGG​AA-30′; V-ATPase d2, forward 5′-AAG​CCT​TTG​TTT​GAC​GCT​GT-3′ and reverse 5′-TTC​GAT​GCC​TCT​GTG​AGA​TG-3′; V-ATPase a3, forward 5′-TGG​CTA​CCG​TTC​CTA​TCC​TG-3′ and reverse 5′-CTT​GTC​CGT​GTC​CTC​ATC​CT-3′;

### Cytotoxicity assay

To verify the effect of the NCTD on cell state, we detected cell viability with Cell Counting Kit-8 (CCK8) kit (Bioshap Life sciences, China). BMMs were seeded onto 96-well plates, and the control group and experimental group with gradient NCTD concentrations were devised, with three replicates in each group. After 48 or 96 h of cell growth, a complete medium containing CCK8 reagent was prepared at 10:1. The original culture medium of cells was discarded, and 100ul culture medium containing CCK8 reagent was added to each well. The 96-well plate was transferred to an incubator at 37°C and incubated for 1 h–4 h. Then the values in the 96-well plate were read on a multifunctional microplate reader and calculated with Microsoft Excel.

### F-actin ring assay of osteoclasts

Before the assay, the bone slices soaked in 70% alcohol were taken out and put in a 96-well plate to dry. After alcohol was volatilized, BMM was seeded on the bone fragment in the 96-well plate, and the BMM was induced into mature osteoclasts with cytokines as above shown. After the formation of osteoclasts, the cells were fixed with 4% paraformaldehyde at 4°C for 30 min, then the paraformaldehyde was discarded and washed twice with PBS. After the cell was permeabilized by 0.2% Triton X-100, the cells on the bone slices were stained with FITC-phalloidine and DAPI respectively. Finally, the bone slices were mounted on the glass slides with the Antifade solution (Bytotime Biotechnology, China) and observed with a fluorescence confocal microscope (Olympus FV3000).

### Western blotting

To find the signal pathway targeted by NCTD during osteoclast differentiation and verify the effect of NCTD on osteoclast differentiation, target protein of BMMs through different treatments were quantified by Western Blotting as we reported previously (Zhou et al., 2018a). The protein bands was scanned by a BIORAD chemiluminescence imager. GAPDH was used as an internal reference, and protein expression in cells can be relatively quantified by band thickness using ImageJ.

### Bone resorption assay

The bone slices were plated in 96-well plates and 1.5 × 104 BMM were seeded on the bone slices. In the slices, the BMM was induced into osteoclasts by cytokines M-CSF and RANKL. After the formation of osteoclasts, mechanical force and ultrasound were used to remove the cells on the surface of bone slices. The bone slices were fixed with 4% paraformaldehyde for 20 min, and then treated with 3% H2O2 for 10 min. The bone slices were incubated with WGA, SABC, and DAB successively, and the excess staining solution was washed with distilled deionized water. Ultimately, the slides were mounted with 50% glycerol and observed under the optical microscope.

### Establishment of ovariectomized-induced osteoporosis model in mice

The mice used in this study were C57BL/6 from SLAC company, and the number of mice used was 24. The raising of mice and experimental design were approved by the Animal Care and Use Ethics Committee of Zhejiang University. OVX were performed to mimic postmenopausal osteoporosis in humans. All experimental mice were divided into four groups, namely SHAM group, OVX group, low dose (LD) NCTD group and high dose (HD) NCTD group, with six mice in each group (*N* = 6). Specifically, mice in SHAM group underwent sham operation and intraperitoneal injection of PBS with the same amount as NCTD of other groups. In OVX group, mice were only injected with PBS equal to NCTD after OVX; LD group was intraperitoneal injection of 1 mg/kg NCTD after OVX; The HD group was administrated 2.5 mg/kg NCTD after OVX. All mice were injected with PBS/NCTD twice a week and euthanized after 6 weeks. One in the HD group was died during NCTD treatment, while the left 23 mice were collected for following analysis.

### Histological analyses

As mentioned above, the left femur of mice was decalcified and paraffin embedded (Zhou et al., 2018b). The paraffin blocks were placed in the refrigerator at −20°C for more than 30 min, and the paraffin slicing machine was used to cut the paraffin blocks into 5 μm tissue sections. Paraffin sections were immersed in xylene, gradient concentration of ethanol and distilled deionized water respectively for removing paraffin. Then the sections were stained with hematoxylin and eosin (H&E) and TRAP and observed with a light microscope. H&E staining and Trap staining were used to analyze the histomorphological characteristics of bone using ImageJ.

### Micro-CT scanning

The left tibia of mice was placed in a scanning tube filled with 70% ethanol in an orderly manner, and the samples were scanned by a high-resolution microscopic CT scanner (SKYSCAN1272). The specific parameters of the left tibia were bone volume/tissue volume (BV/TV), connectivity density (Conn.Dn), structural model index (SMI), trabecular number (Tb.N), trabecular thickness (Tb.Th) and trabecular separation (Tb.Sp). The scanning thickness was 12 μm, the interval was 300 ms and the voltage was 70 kV. 3D reconstruction and measurement were performed on the 2D thin layer image of bone tissue, and the threshold was set at 220–250 to distinguish bone tissue from soft tissue.

### Reactive oxygen species assay

ROS Kit (Boster Bio, Wuhan, China) was used to detect reactive oxygen species. BMMs obtained in accordance with the above procedures were seeded in 24-well plates, and after cell adherence, the cells were pretreated with 2 μM NCTD for 4 h–6 h. DCFH-DA was diluted in serum-free medium at 1:1,000 and added into BMM cell culture to load probes into cells. Finally, DCF fluorescence was observed by fluorescence microscope after cells were treated with cytokine RANKL for 20 min. The data was analyzed using ImageJ.

### Statistics

All data in this study were represented by mean ± SD. All experiments have been repeated for at least three times. These data were statistically analyzed by Graphpad prism 9. The differences between the two group of data were compared using the unpaired student’s *t*-test. ONE WAY ANOVA was used to compare the differences among more than two groups. *p* < 0.05 was considered a statistically significant difference.

## Results

### Norcantharidin suppressed osteoclastogenesis at a nontoxic concentration

**FIGURE 1:**
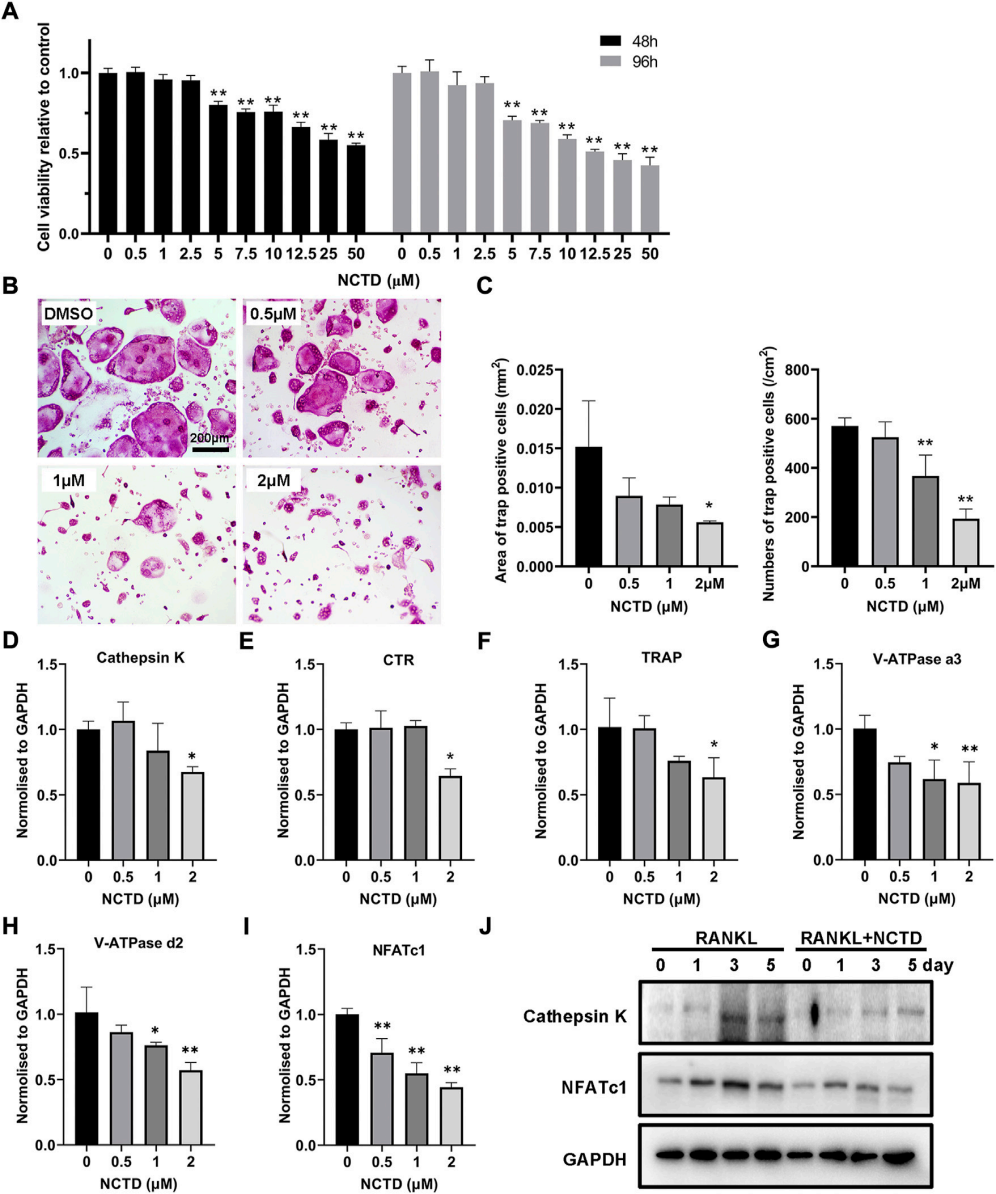
NCTD affected osteoclast formation in BMM in a non-toxic range. **(A)** After adding gradient concentration of NCTD into BMM cell culture, CCK8 kit was used to detect the cell viability to figure out the non-toxic range of NCTD for BMM (*n* = 5). **(B)** BMM cells were induced into osteoclasts with cytokines M-CSF and RANKL, and DMSO/NCTD was added to observe the effect of NCTD on osteoclastogenesis. **(C)** The area and number of osteoclasts induced by BMM were quantified. **(D–I)** After BMM was treated with different concentrations of NCTD, the osteoclast makers of Cathepsin K, CTR, TRAP, V-ATPase a3, V-ATPase d2, and NFAT c1 were detected by fluorescence quantitative PCR. **(J)** Western blotting was used to investigate the effects of NCTD addition on Cathepsin K and NFATc1 during osteoclast formation. All experiments were repeated independently for three times. Values are expressed as mean ± SD; *p < 0.05, **p < 0.01 vs. the control group.

For cell viability detection, the BMM was seeded in 96-well plates, and the cells were treated with different concentrations of NCTD. The relative decrease of cells could be observed by the Microplate Reader. As shown in the figure, the number of cells in the well plate decreased obviously when the concentration of NCTD was more than 2.5 μM (*p* < 0.01) (Figure 1A). In the process of osteoclastogenesis, NCTD at the concentrations of 0.5, 1, and 2 μM resulted in a significant reduction in the formation of osteoclasts (Figure 1B). The area and number of osteoclasts were quantified by ImageJ, and the histogram showed that NCTD could reduce the area and number of osteoclasts induced by NCTD in dose-dependent manners (Figure 1C). Through fluorescence quantitative PCR detection of osteoclast formation markers in BMM treated with different concentrations of NCTD, it was observed that the expression of these genes (Cathepsin K, CTR, TRAP, V-ATPase a3, V-ATPase d2 and NFAT c1) would be down-regulated with the increase of drug concentration (Figures 1D–I). In Western blotting assays, NCTD down-regulated protein expression of NFAT c1 and Cathepsin K during osteoclast formation (Figure 1J).As a result, NCTD could affect osteoclastogenesis without damaging cell viability.

### Norcantharidin attenuated F-actin ring formation and osteoclastic bone resorption

**FIGURE 2:**
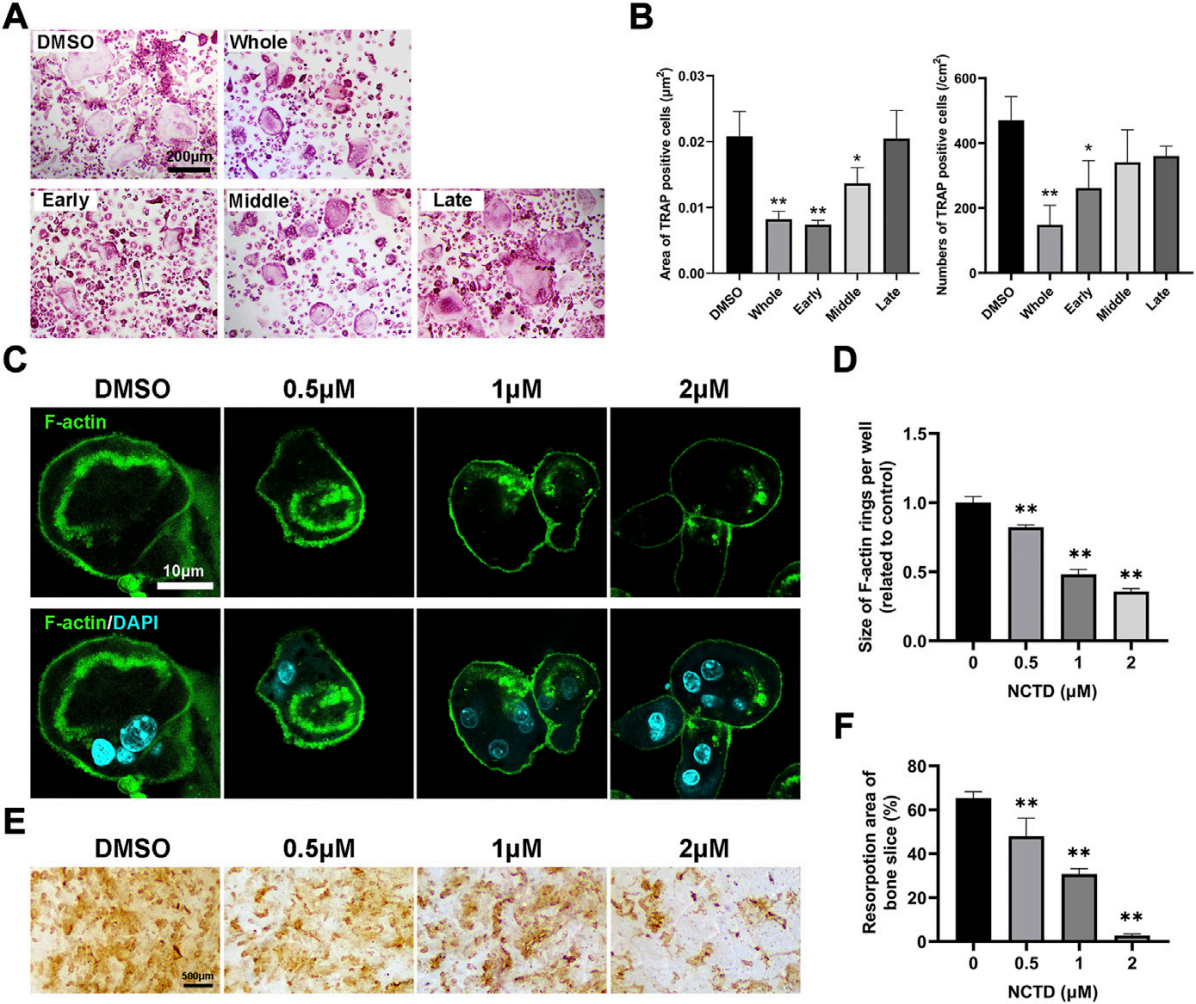
NCTD inhibited the formation of F-actin rings in osteoclasts and inhibited bone resorption. **(A)** The effects of NCTD treatment at different time periods (Early stage, middle stage and late stage) on osteoclast formation were observed with the light microscope. **(B)** The areas and numbers of osteoclasts in BMM cells treated by NCTD at different stages were quantified. **(C)** BMM was induced into osteoclasts on bone slices. After treatment with different concentrations of drugs, FITC-Phalloidin and DAPI staining were performed and observed by fluorescence microscope. **(D)** While observing the F-actin ring in the process of BMM inducing osteoclast, the size of F-actin ring was quantified. **(E,F)** BMM was induced into osteoclasts on bone slices, and the effect of different concentrations of NCTD on bone resorption was observed. Furtherly, the results of bone resorption were further quantified. All experiments were repeated independently for three times. Values are expressed as mean ± SD; *p < 0.05, **p < 0.01 vs. the control group.

To explore which stage NCTD affects during osteoclastogenesis, we treated cells with NCTD at different stages: early stage, middle stage and late stage. Briefly, for early stage group, NCTD was used at day 0 to day 2; For the middle group, NCTD was used at day 2 to day 4; Dor the late group, NCTD was used at day 4 to day 6; For the whole group, NCTD was used at day 0- day 6; For the DMSO group, cells were induced into osteoclasts without NCTD treatment. TRAP staining and data quantification found that NCTD treatment in the early and middle stages significantly attenuated osteoclasts size, while NCTD treatment in the early stage reduced the number of osteoclasts (Figures 2A,B). The F-actin ring, a marker of mature osteoclast, could be observed under the microscope after staining the cells with FITC-Phalloidin. The F-actin ring is a major part of the cytoskeleton, closely related to osteoclast migration and bone resorption (Lakkakorpi and Vaananen, 1991). After being seeded on bovine bone slices, osteoclasts would phagocytose the bone slices and form bone absorption pits or traces. As shown in Figures 2C,D, F-actin rings became smaller and incomplete when osteoclasts were treated with NCTD. In consist with the results above, bone resorption assay also showed decreased resorptive pits after NCTD treatment (Figures 2E,F).

### Norcantharidin inhibited osteoclast formation by downregulating ROS and NLRP3 inflammasomes formation

**FIGURE 3:**
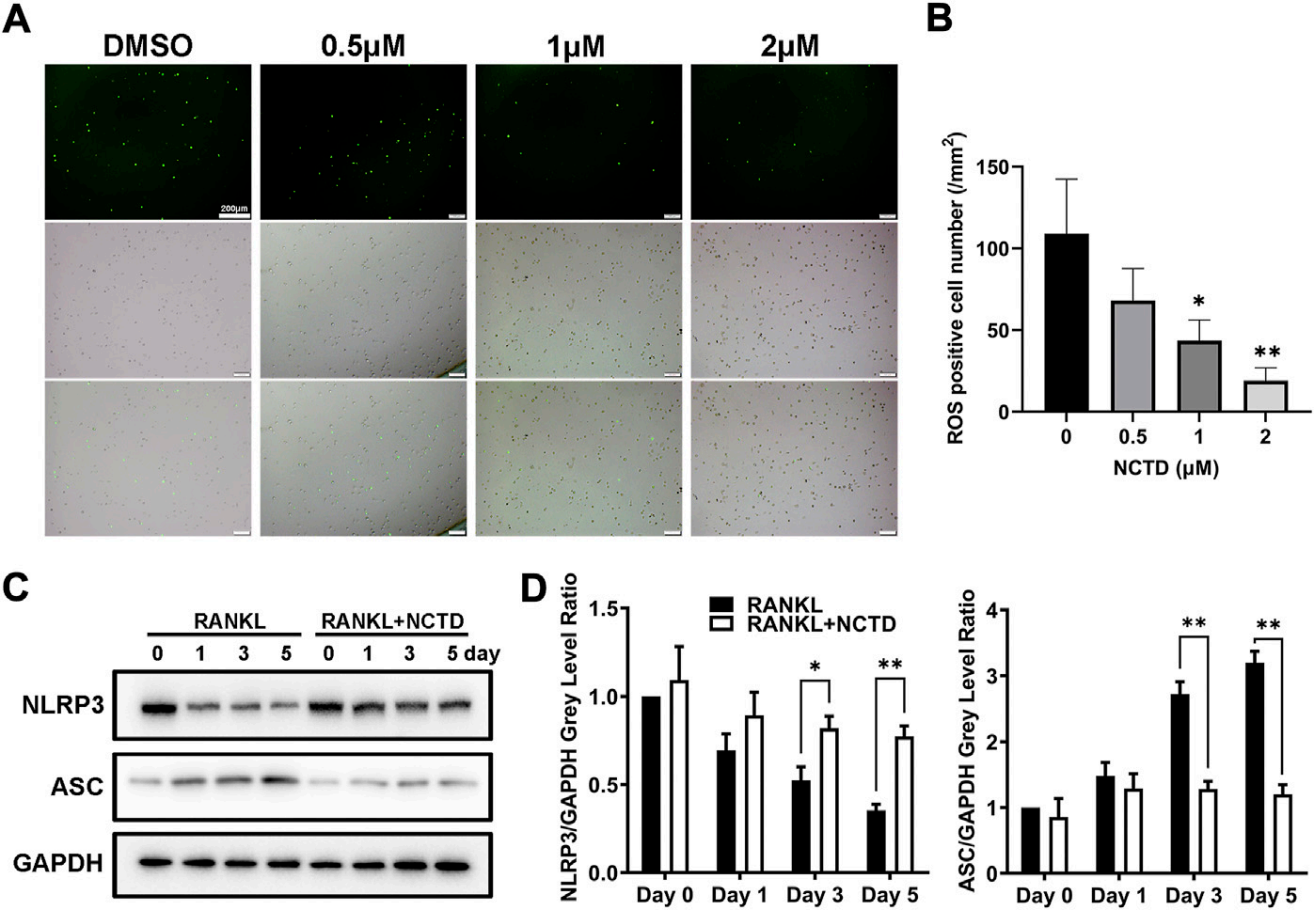
NCTD reduced the content of ROS and inhibited the formation of inflammasome in BMM cells. **(A)** During BMM differentiating into osteoclast with RANKL, ROS kit was used to detect the ROS content in BMM. **(B)** The ROS observed under fluorescence microscope was quantified. **(C)** Western blotting was used to measure NLRP3 and ASC expression level in BMM induced osteoclasts at day 0, 1, 3, and 5. **(D)** The NLRP3 and ASC bands of Western blotting were quantified. All experiments were repeated independently for three times. Values are expressed as mean ± SD; *p < 0.05, **p < 0.01 vs. the control group.

By TRAP staining, quantitative PCR and Western blotting of BMM cells, we found that NCTD could inhibit osteoclast formation and the expression of osteoclast-related markers. ROS is the product of oxygen metabolism. When the content of ROS is excessive, the body will be in an unbalanced state of antioxidation-oxidation, which will increase osteoclasts and promote bone absorption (Toker et al., 2012). In ROS assays, we found that NCTD could reduce RANKL-induced ROS production in BMM cells (Figures 3A,B). Therefore, it is speculated that NCTD might inhibit osteoclast formation by down-regulating RANKL-induced ROS levels. NLRP3 and ASC are important substances that form inflammasomes, which regulate osteoclast differentiation and bone resorption. The expression of NLRP3 gradually decreased during osteoclast induction, but the treatment of NCTD suppressed the reduction of NLRP3 under the stimulation of RANKL (Figures 3C,D). After quantifying the gray values of protein bands in Western blotting of ASC and NLRP3, we found that NCTD upregulated the reduced expression of NLRP3 under the treatment of RANKL, as well as down-regulated the RANKL-induced increase of ASC, indicating that NCTD reduced RANKL-induced production of the inflammasome. Consequently, NCTD inhibits osteoclast by affecting RANKL-induced ROS production and inflammasome formation in BMM cells.

### Norcantharidin inhibited osteoclast formation by the extracellular signal-regulated kinase signaling

**FIGURE 4:**
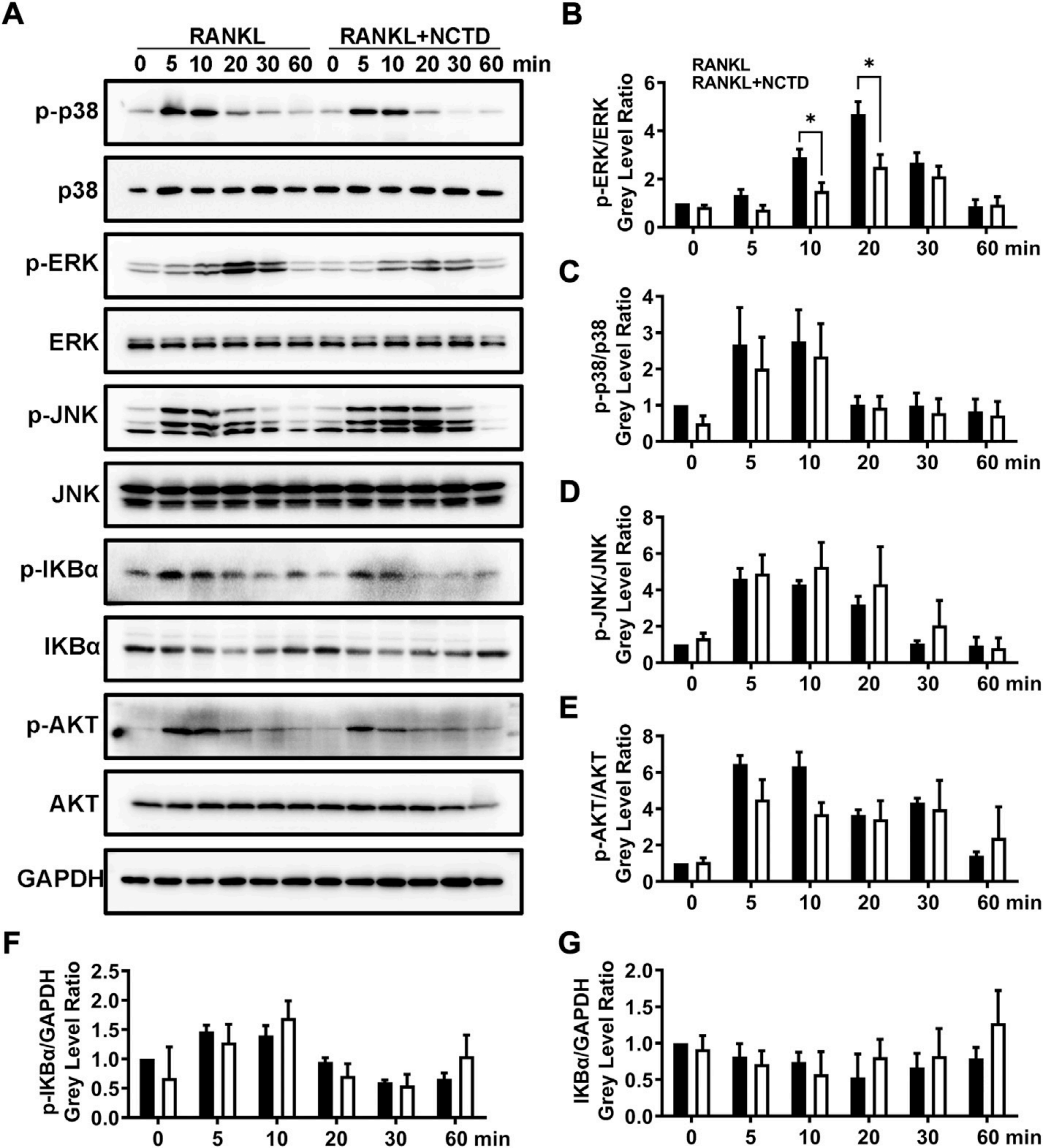
NCTD can downregulate the expression of ERK in BMM cells during osteoclast formation. **(A)** Western blotting was used to measure p-p38, p38, p-ERK, ERK, p-JNK, JNK, p-IKBα, IKBα, p-AKT, AKT and GAPDH expression level on BMMs after RANKL stimulating at 0, 5, 10, 20, 30 and 60 min. **(B)** The phosphorylation degree of p-ERK can be showed by the p-ERK /ERK gray value ratio in Western blotting. **(C)** Western blotting bands of P-P38/P38 were quantified. **(D)** The phosphorylation degree of P-JNK can be showed by the p-JNK/JNK gray value ratio in Western blotting. **(E)** The phosphorylation degree of P-AKT can be showed by the p-AKT/AKT gray value ratio in Western blotting. **(F)** The Western blotting bands of P-IKBα/GAPDH were quantified and represented by histograms. **(G)** The Western blotting bands of IKBα/GAPDH were quantified. All experiments were repeated independently for three times. Values are expressed as mean ± SD; *p < 0.05, **p < 0.01 vs. the control group.

In order to find out which signaling pathway NCTD acts in the process of inhibiting osteoclast formation, we used Western blotting to analyze. We measured the phosphorylation level of P38, extracellular signal-regulated kinase (ERK), JNK, IKBα, and AKT by Western blotting, and found that only ERK protein was significantly downregulated after NCTD treatment (Figures 4A–G). ERK is a member of the MAPK family, and its related signaling pathways are involved in cell proliferation and differentiation, cytoskeleton formation, apoptosis and cell canceration. ERK signaling pathway can promote osteoclast activation, which is closely related to the formation of osteoporosis (Wang et al., 2021). ImageJ was used to detect the gray values of the bands in Western blotting, and significant differences were found in ERK protein expression after 10 and 20 min of NCTD treatment, while no significant differences were found in other proteins. These results indicated that ERK signaling pathway is an important way through which NCTD reduces ROS production and affects osteoclast activation.

### Norcantharidin prevented OVX-induced osteoporosis in mice by reducing osteoclast formation

**FIGURE 5:**
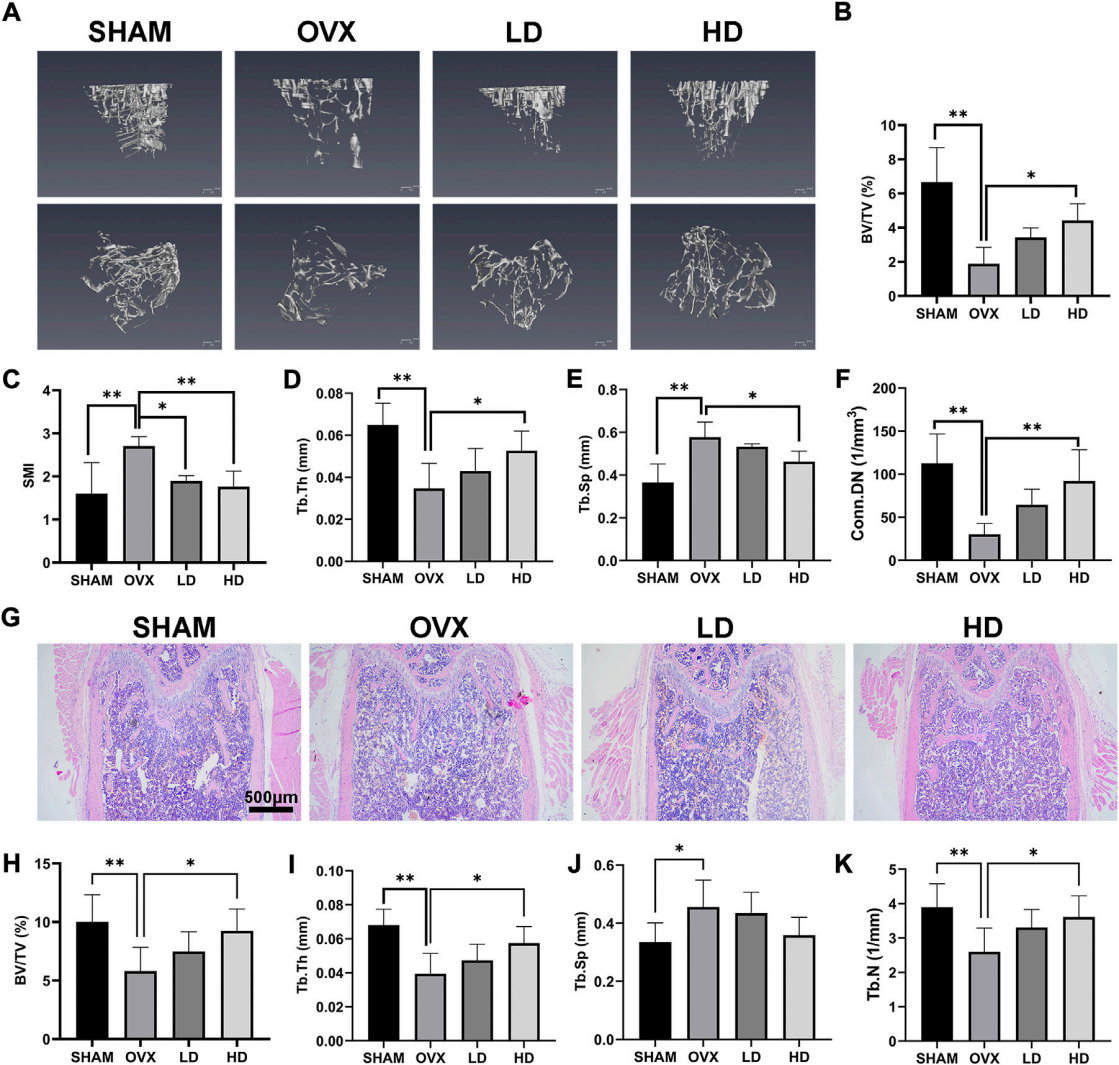
NCTD can alleviate OVX-induced osteoporosis in mice. **(A)** After the osteoporosis model was established after OVX surgery, the left tibia of the mice was taken for micro-CT scanning after intraperitoneal injection of PBS/NCTD. **(B)** BV/TV (%) were quantified after micro-CT scanning of the tibia. **(C)** SMI results of micro-CT scanning of tibia in mice. **(D)** Tb.Th (mm) quantification of micro-CT scanning. **(E)** Quantitative histogram of micro-CT scanning results Tb.Sp(mm). **(F)** Conn.DN (1/mm^3^) quantification of micro-CT scanning. **(G)** After OVX surgery, the mice were established into osteoporosis model and then the mice were intraperitoneally injected with NCTD/PBS. The left femur of the mice was taken for paraffin section and H&E staining. **(H–K)** The BV/TV (%), Tb.Th (mm),Tb.Sp (mm),Tb.N (1/mm) in H&E staining results of mouse femur were quantitatively analyzed and their according histogram was made. Values are expressed as mean ± SD, *n* = 5–6; *p < 0.05, **p < 0.01 vs. the control group.

In order to investigate the role of NCTD in OVX-induced osteoporosis in mice, a 3D tibial model was reconstructed by micro-CT analysis, and the data of BV/TV (%), SIM, Tb.Th (mm), Tb.Sp (mm) and Conn.Dn (1/mm^3^) were obtained by microscopic CT scanner. As shown in Figure 5A, 3D reconstruction showed that OVX could cause the bone density reduction and osteopenia of tibia in mice, which were improved after NCTD treatment in LD and HD groups. Micro-CT analysis showed that BV/TV, TB. Th and CONN. Dn all decreased after OVX, and were reversed after treatment with NCTD in the low-dose (1 mg/kg) and high-dose (2.5 mg/kg) groups (Figures 5B,D,F). In contrast, SMI and Tb.Sp increased after OVX treatment and decreased after NCTD treatment (Figures 5C,E). According to micro-CT scan results, NCTD can prevent the progression of osteoporosis and inhibit bone absorption, and the higher the concentration of NCTD, the better the therapeutic.

To further explore the role of NCTD in the OVX-induced mouse model of osteoporosis, the femurs of mice were histologically analyzed by H&E staining. H&E staining results showed that the bone trabecular thinning and osteopenia occurred in the mouse femur after OVX, which were improved after NCTD treatment with low (1 mg/kg) and high dose (2.5 mg/kg), and the values of BV/TV, Tb.TH, TB.SP, and TB.N were consistent with the results of micro-CT analysis (Figures 5G–K).

**FIGURE 6:**
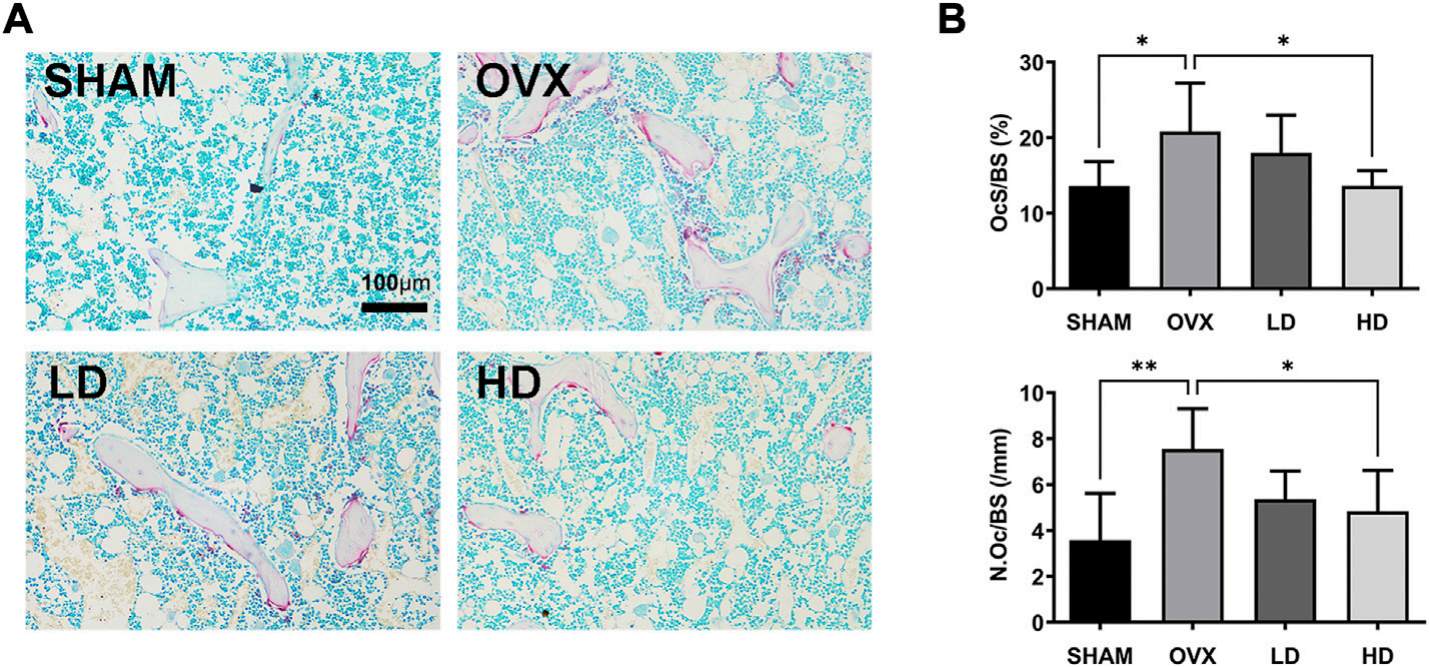
NCTD attenuates osteoclast formation *in vivo*. **(A)** The representive images of TRAP staining in each group. **(B)** The N.Oc/BS and OcS/BS were measured with TRAP-stained sections. in each group. Values are expressed as mean ± SD, *n* = 5–6; *p < 0.05, **p < 0.01 vs. the control group.

Next, TRAP staining is performed to confirmed the effects of NCTD. As showed in Figure 6, the number of osteoclasts increased after OVX, while NCTD significantly inhibited OVX-induced osteoclast formation in a dose-dependent manner.

## Discussion

Excessive osteoclast formation and/or function are the major causes of imbalanced bone homeostasis, resulting in a number of pathological osteolytic diseases, such as osteoporosis, rheumatoid arthritis, periprosthetic osteolysis (Crotti et al., 2004; McHugh, 2017). Osteoporosis is the most common osteoclastic disorder in clinic, which causes a substantial economic burden. Therefore, diverse drugs are investigated for clinical osteoporotic treatment. However, various adverse effects limit the extensive application of these drugs, which increases the demand of developing a new anti-osteoporotic agent with fewer side-effects. In this study, we found that NCTD exhibits suppressive effects on RANKL-induced osteoclast formation and related bone resorption. Moreover, NCTD achieves the effects by targeting ERK and ROS/NLRP3 pathways. Additionally, NCTD exerts a beneficial effect on preventing OVX-induced osteoporosis through attenuating osteoclast activity *in vivo*. Thus, our *in vitro* and *in vivo* study summarized that NCTD might be a potential agents for the treatment of osteoporosis.

Osteoclast plays a crucial role in bone metabolism. When RANKL binds to its receptor RANK, osteoclastogenesis initiates, subsequently activating a series of downstream signaling pathways, including the MAPK (ERK, JNK, p38) and NF-κB pathways, resulting in the cell fusion, osteoclast maturation and osteoclastic bone resorption. The ERK signaling pathway is critical for survival, differentiation, polarity and function of osteoclasts, which involves two forms, ERK1 and ERK2 (Lee et al., 2018). The phosphorylation of ERK controls a number of transcription factors, including the key factors NFATc1 and c-fos, which finally promoting osteoclastogenesis (Lee et al., 2009). Previous studies have confirmed that NCTD exerts inhibitory effects in multiple cells (Zheng et al., 2014; Peng et al., 2016). In the present study, we explored the effects of NCTD on the ERK signaling during RANKL-stimulated osteoclastogenesis. As expected, the Western Blotting results confirmed that NCTD attenuated the phosphorylation level elevation of ERK markedly. However, NCTD did not influence the phosphorylation of the NF-κB, p38 and JNK signaling pathways.

ROS has been confirmed to participate in osteoclast formation and function by modulating a series of signaling cascades (Garrett et al., 1990; Bax et al., 1992). The stimulation of RANKL promotes osteoclastogenesis by increasing intracellular ROS levels in BMMs, whereas the utilization of ROS inhibitor, N-acetyl cysteine (NAC), significantly reduced the formation of osteoclast (Lean et al., 2003; Lee et al., 2005). Moreover, ROS also improves osteoclastic bone resorption (Wang X. et al., 2018). In addition, *in vivo* study further confirmed that the suppression of ROS could attenuate the number of osteoclasts and OVX-induced bone loss (Lean et al., 2003). A previous study has found that NCTD could upregulate the ROS level in TSGH 8301 cells (Yu et al., 2012). In contrast, in our study, RANKL-induced elevated ROS levels were blocked by the presence of NCTD. This might be caused by the differences between BMMs and TSGH 8301 cells.

Increased evidence demonstrates that NLRP3 inflammasomes is also involved in osteoclastogenesis and bone resorption, which is consist of NLRP3, pro-caspase-1 and ASC (Schroder and Tschopp, 2010). Bonar et al. (2012) showed that the continuous activation of NLRP3 stimulates osteoclast differentiation *in vivo* and *in vitro*, leading to the elevation of bone resorption and subsequent bone loss. In addition, RANKL-induced activation of NLRP3 inflammasomes exerts positive effects on promoting osteoclastic transcription factors, B lymphocyte-induced maturation protein 1 (Blimp1) and NFATc1, facilitating osteoclast differentiation and maturation (Wang et al., 2016; Wang et al., 2020). In the present study, we found that the RANKL-induced decrease of NLRP3 expression was rescued with the participation of NCTD, as well as the inhibition of elevated ASC levels.

In consistent with the results found *in vitro*, NCTD exhibited a beneficial effect on protecting bone mass in the OVX-induced osteoporosis model. Estrogen deficiency has been regarded as a classical animal model for osteoporosis, with enhanced formation and activation of osteoclasts. As expected, NCTD impaired OVX-induced bone loss in a dose-dependent manner by attenuating osteoclast *in vivo*.

**FIGURE 7:**
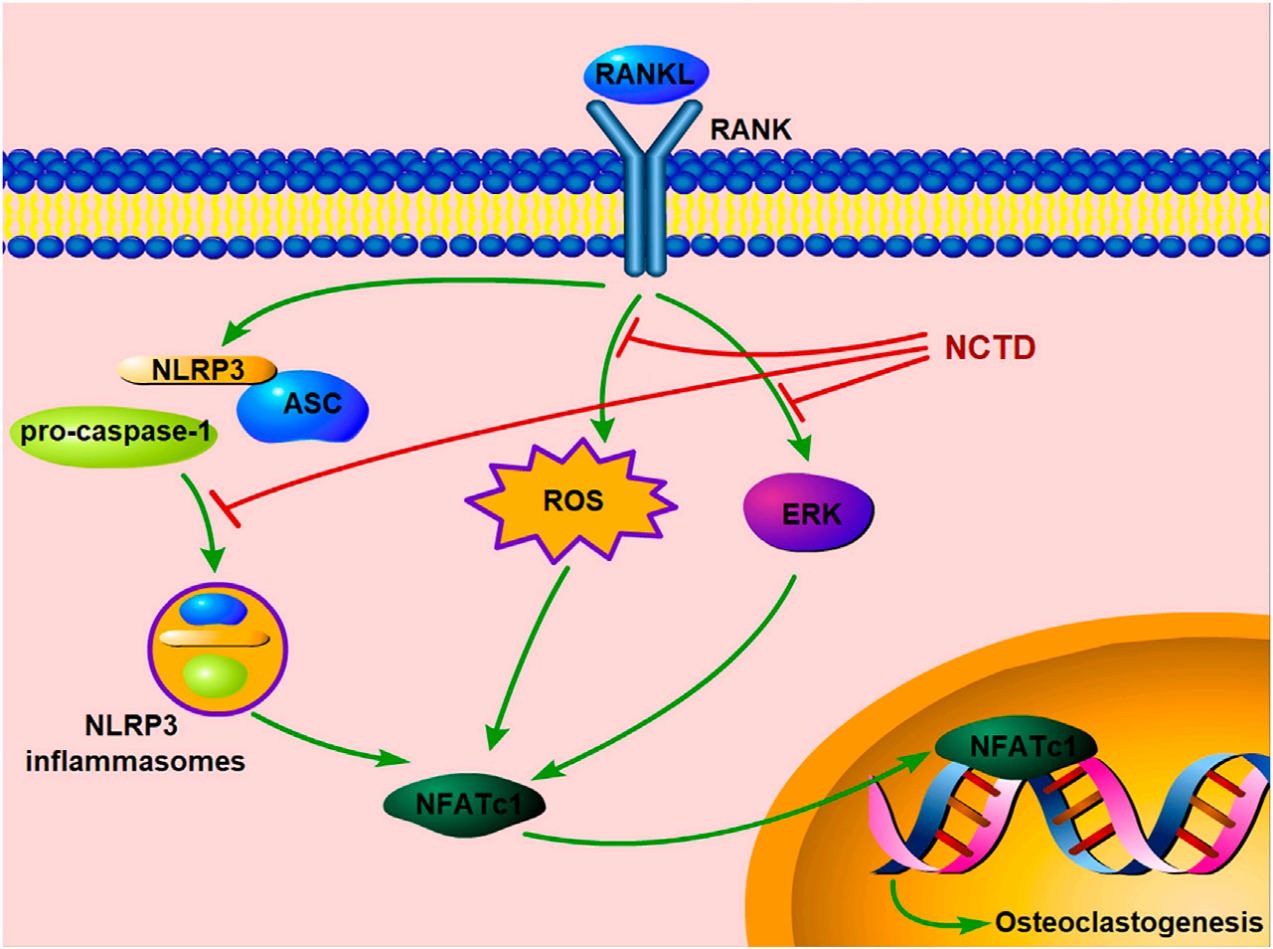
Schematic diagram indicates the potential protective effect of NCTD on osteoporosis.

In conclusion, our study exhibited that NCTD could inhibit osteoclast formation and bone resorption by attenuating the ERK, ROS and NLRP3 inflammasomes pathways *in vitro*. Moreover, our *in vivo* study further confirms its preventive effects on estrogen-deficiency bone loss by inhibiting osteoclast formation and functions (Figure 7). Therefore, we could conclude that NCTD might be a potential candidate for the prevention and treatment of osteoporosis.

## Data Availability

The original contributions presented in the study are included in the article/Supplementary Material, further inquiries can be directed to the corresponding authors.
