# Supplementary figures and images for: Norcantharidin ameliorates estrogen deficient-mediated bone loss by attenuating the activation of extracellular signal-regulated kinase/ROS/NLRP3 inflammasome signaling
Source: Front Pharmacol. 2022 Nov 3;13:1019478. doi: 10.3389/fphar.2022.1019478 (PMC9669383; doi:10.3389/fphar.2022.1019478)

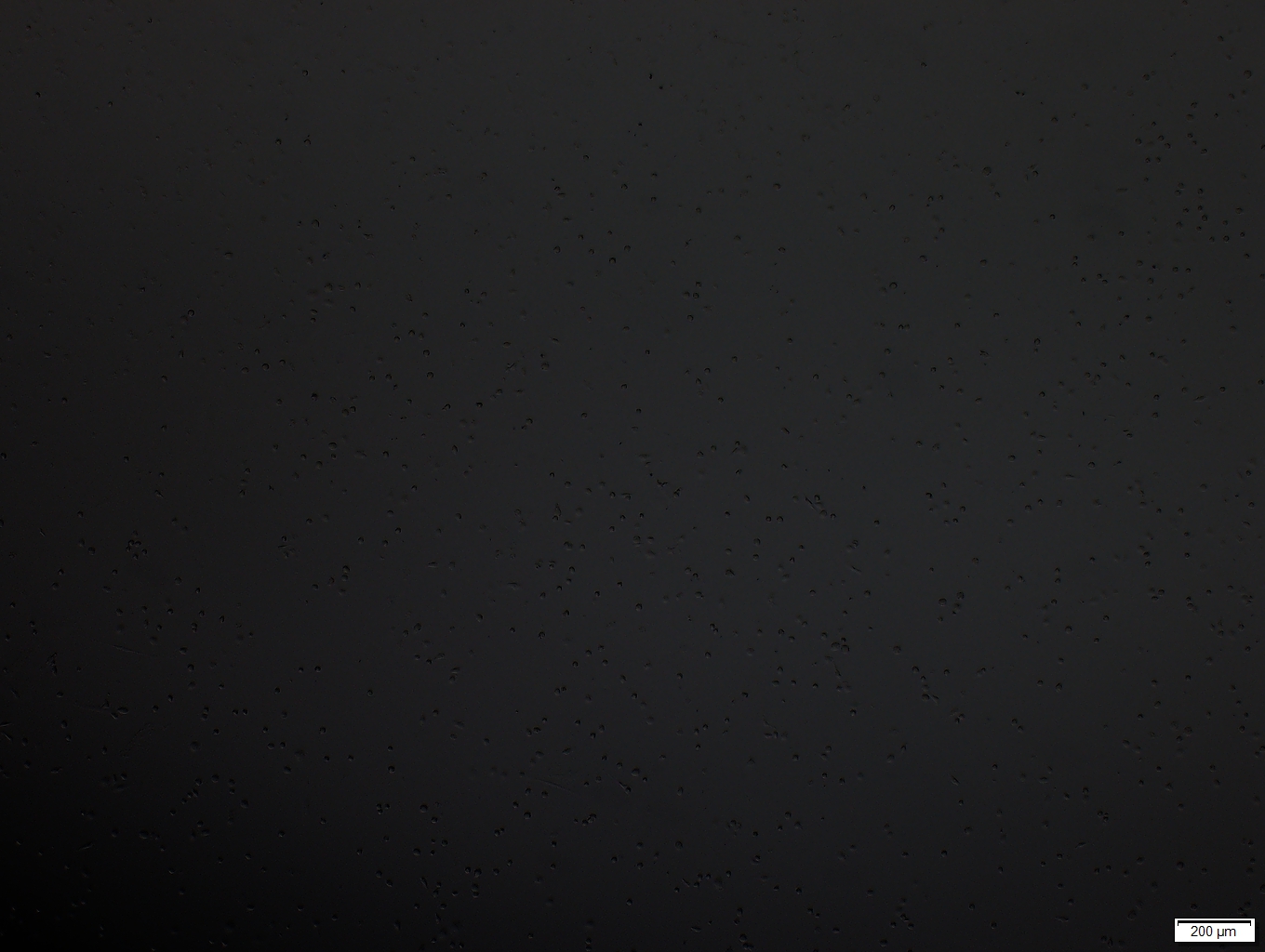

Supplement: Supplementary file 1 [file DataSheet1.ZIP › Figure 3/A-B/0.5-1.jpg]

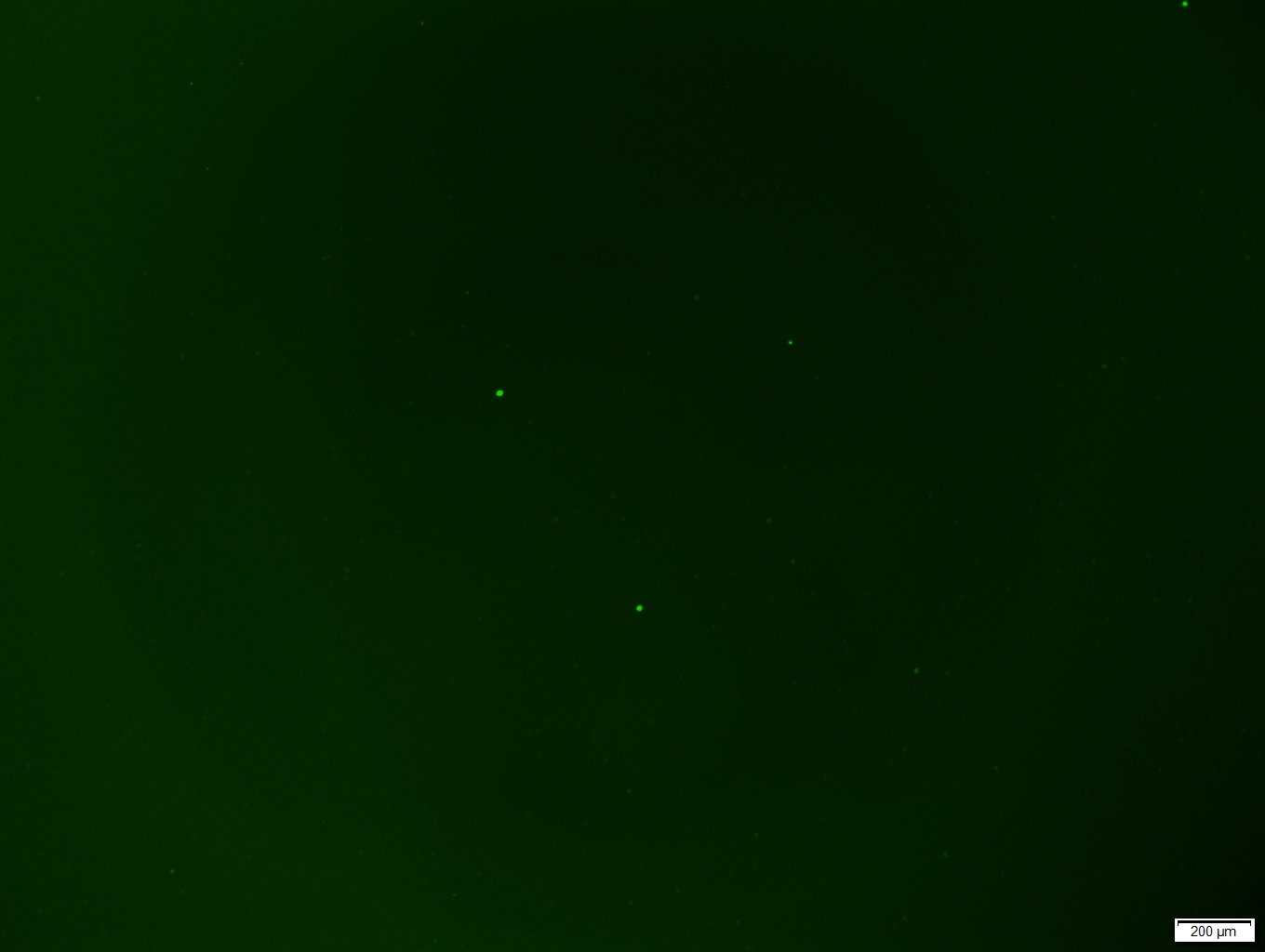

Supplement: Supplementary file 1 [file DataSheet1.ZIP › Figure 3/A-B/0.5.jpg]

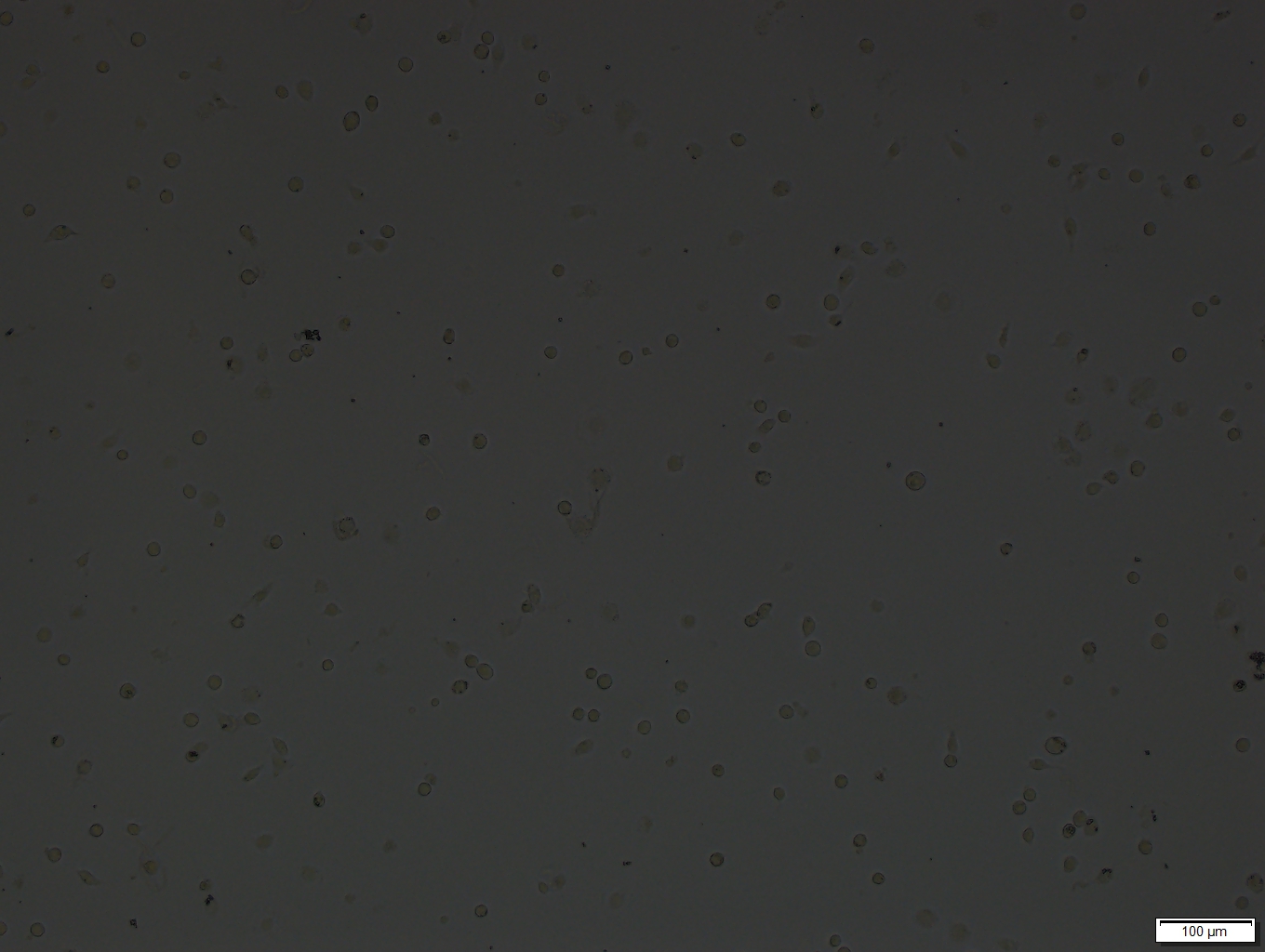

Supplement: Supplementary file 1 [file DataSheet1.ZIP › Figure 3/A-B/1-1.jpg]

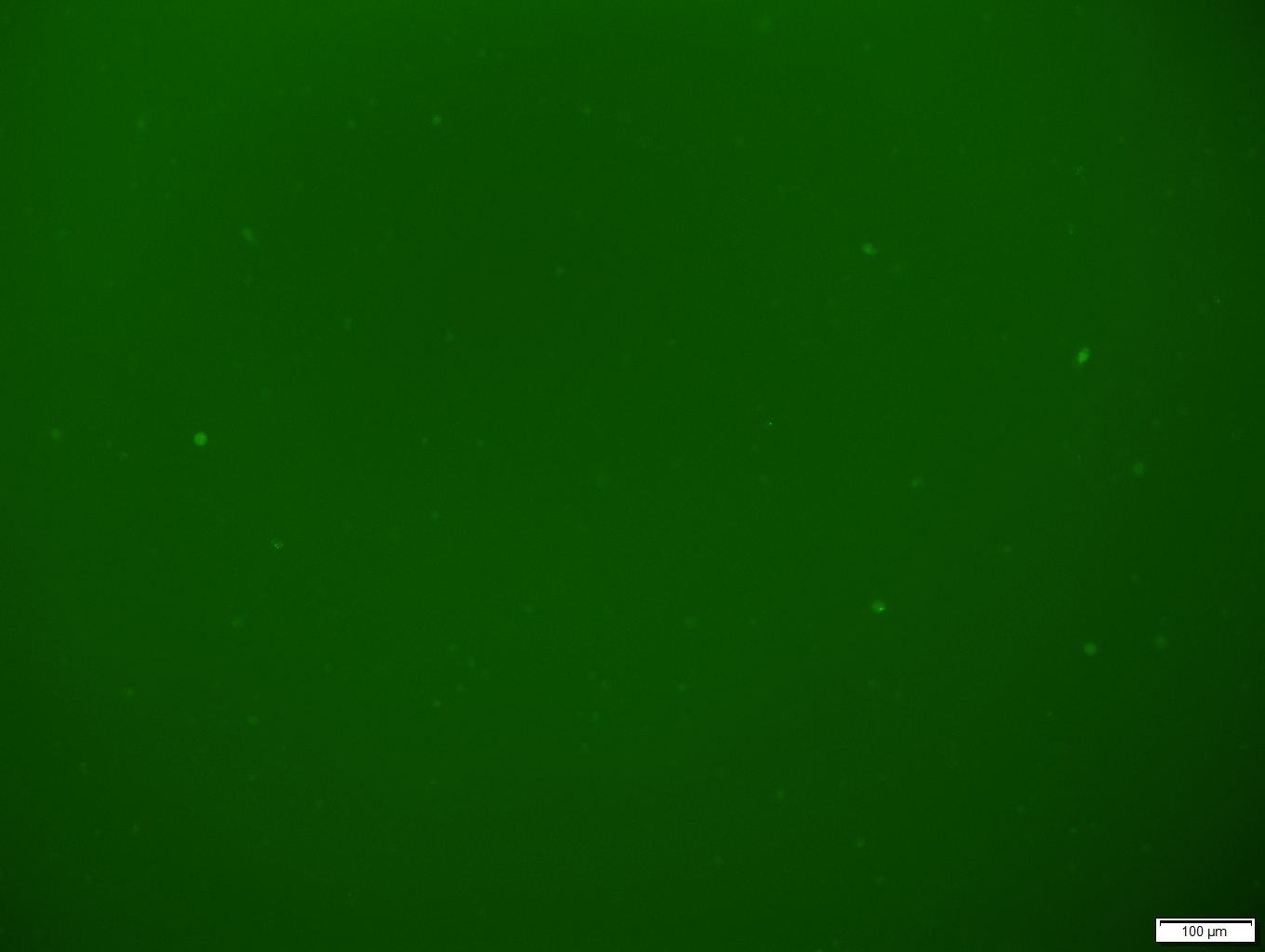

Supplement: Supplementary file 1 [file DataSheet1.ZIP › Figure 3/A-B/1.jpg]

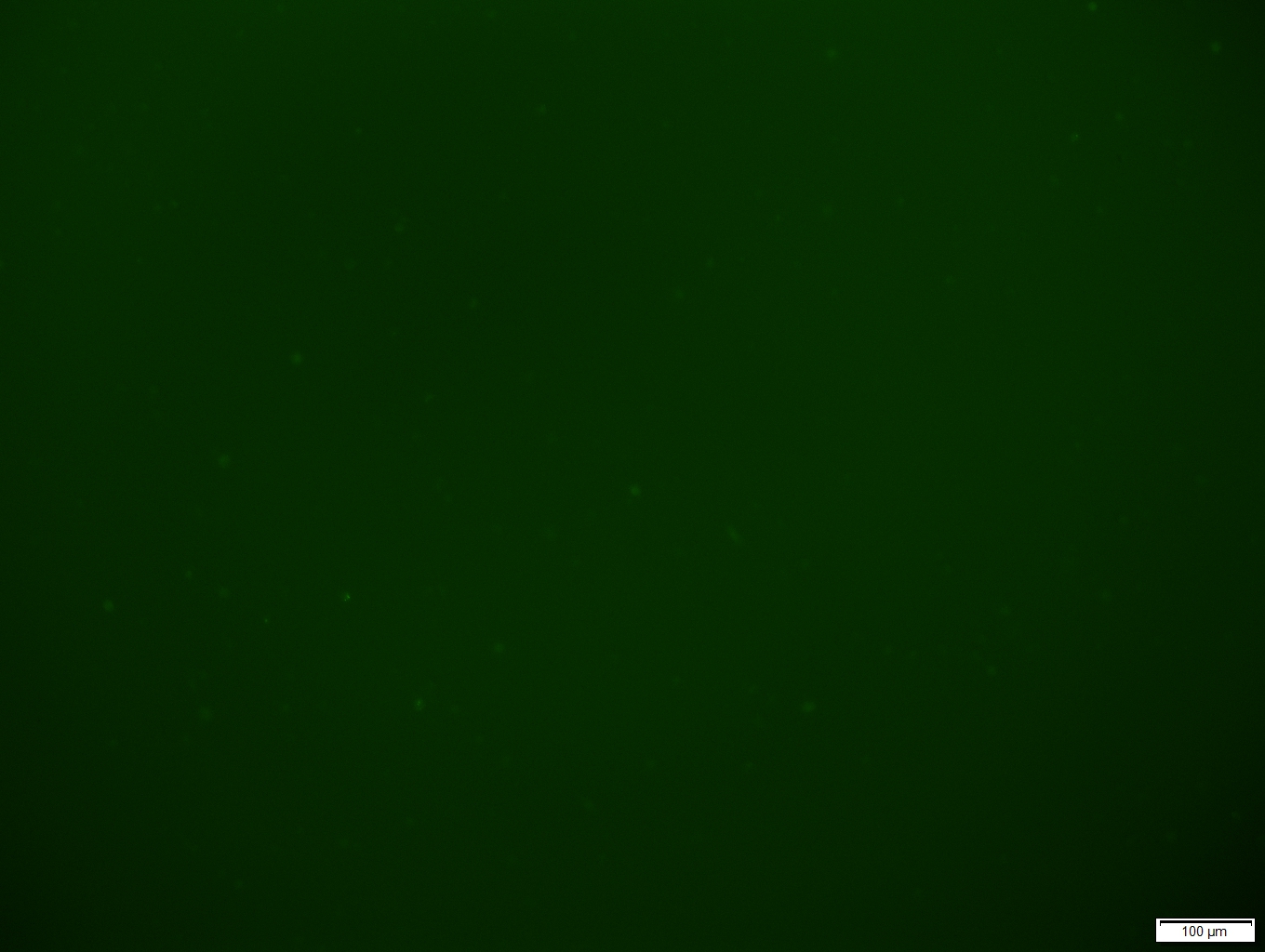

Supplement: Supplementary file 1 [file DataSheet1.ZIP › Figure 3/A-B/2-1.jpg]

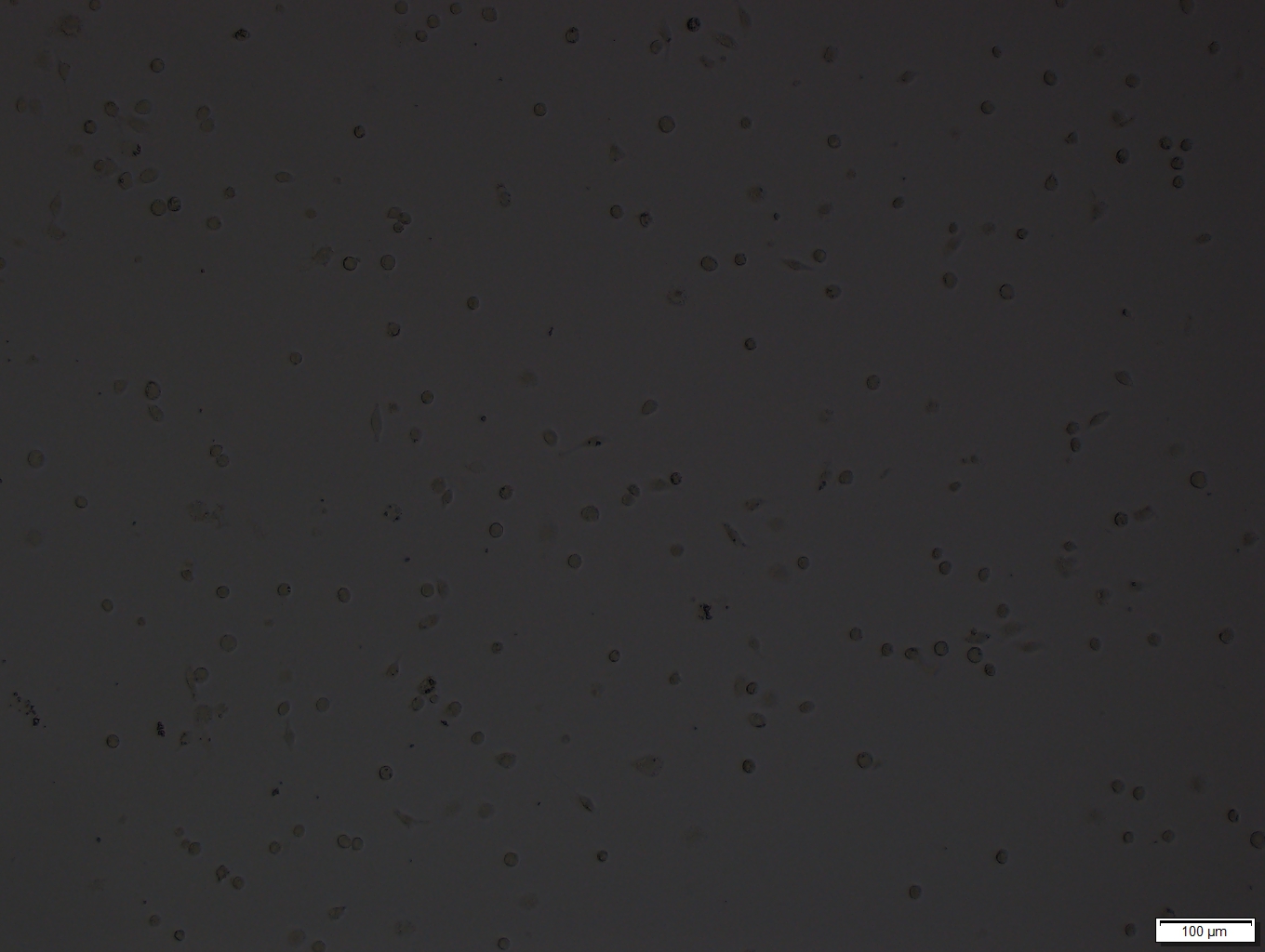

Supplement: Supplementary file 1 [file DataSheet1.ZIP › Figure 3/A-B/2.jpg]

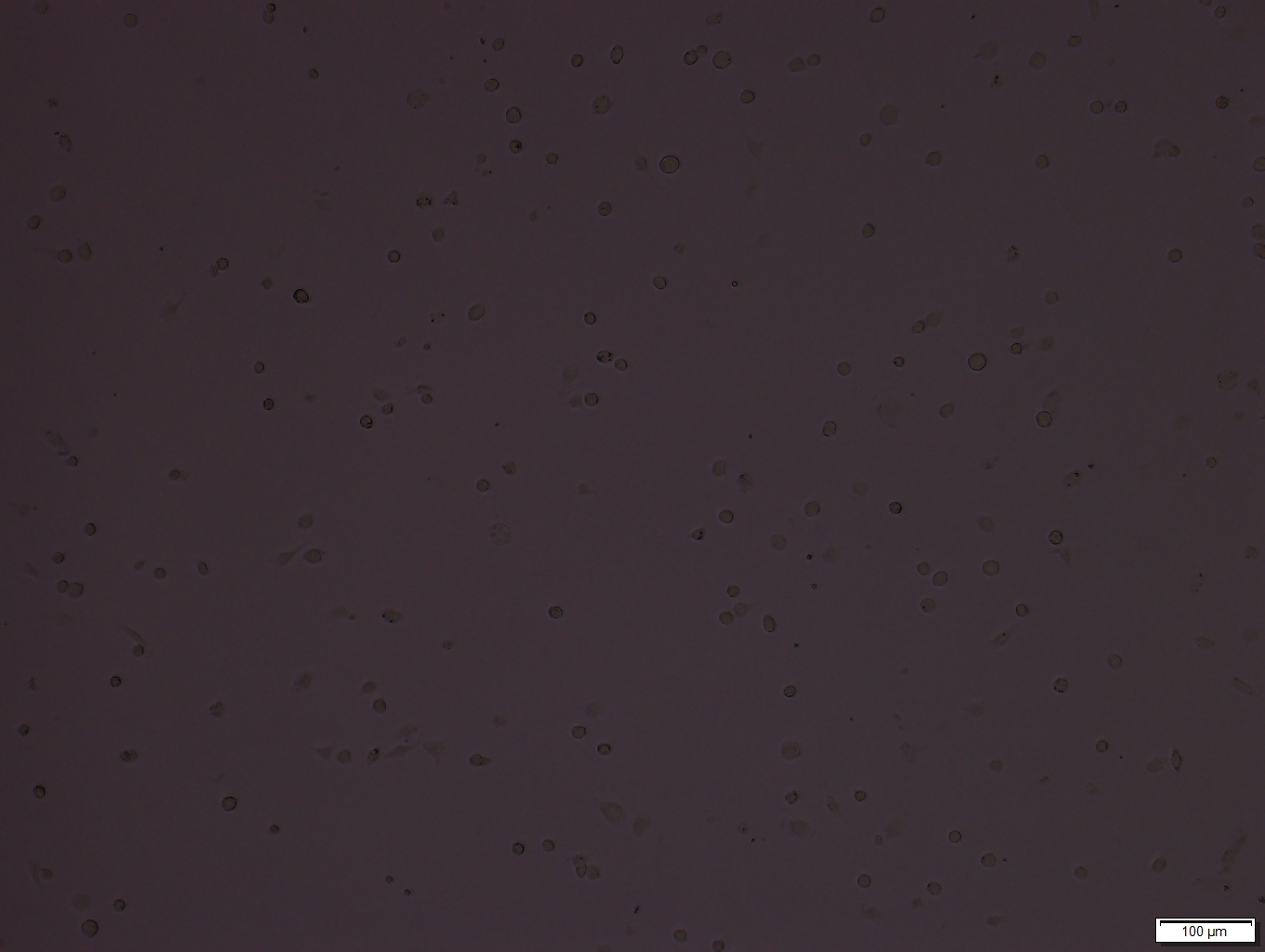

Supplement: Supplementary file 1 [file DataSheet1.ZIP › Figure 3/A-B/dmso-1.jpg]

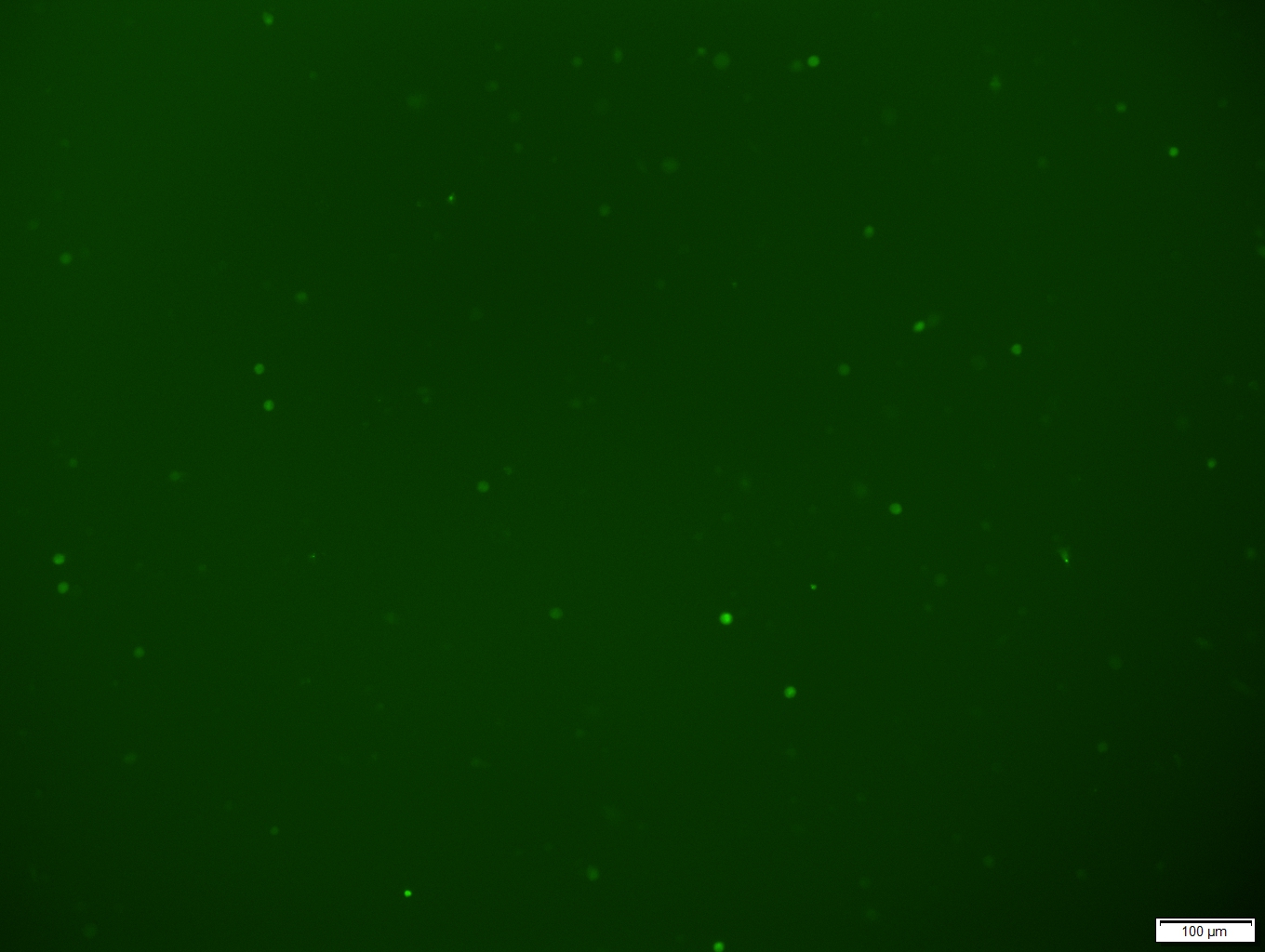

Supplement: Supplementary file 1 [file DataSheet1.ZIP › Figure 3/A-B/dmso.jpg]

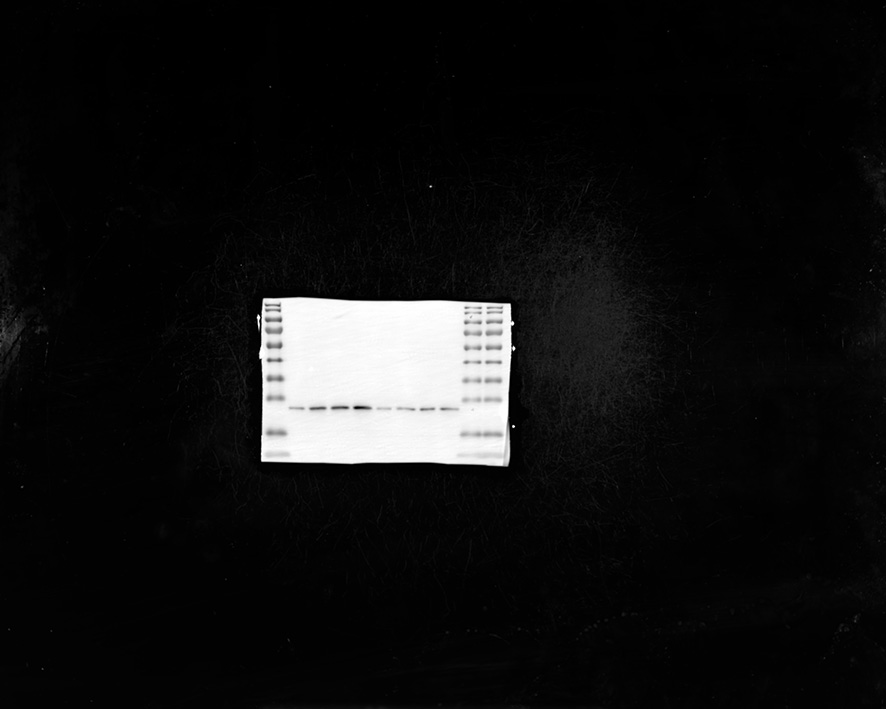

Supplement: Supplementary file 1 [file DataSheet1.ZIP › Figure 3/C-D/ASC-1.jpg]

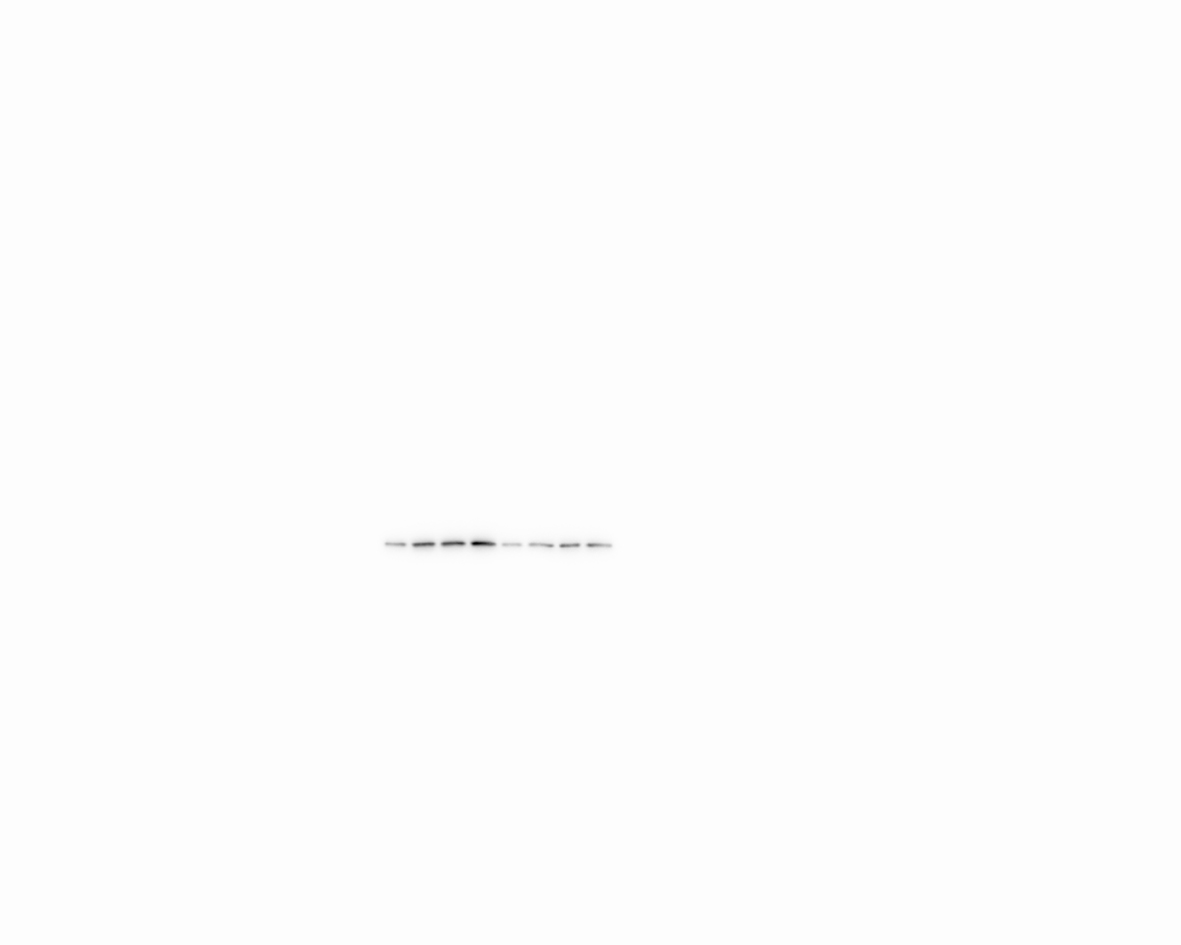

Supplement: Supplementary file 1 [file DataSheet1.ZIP › Figure 3/C-D/asc.jpg]

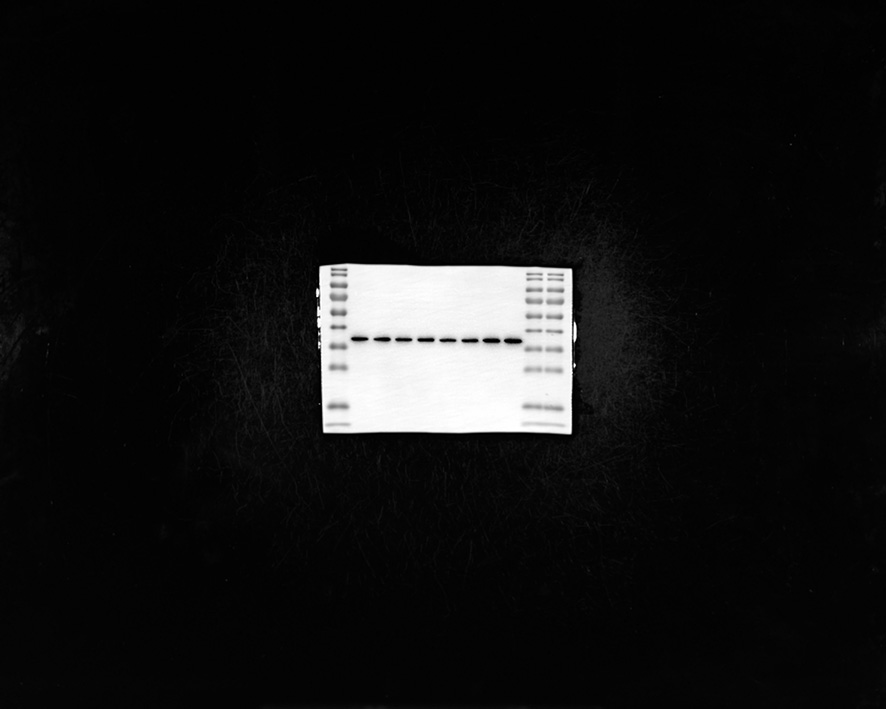

Supplement: Supplementary file 1 [file DataSheet1.ZIP › Figure 3/C-D/GAPDH-1.jpg]

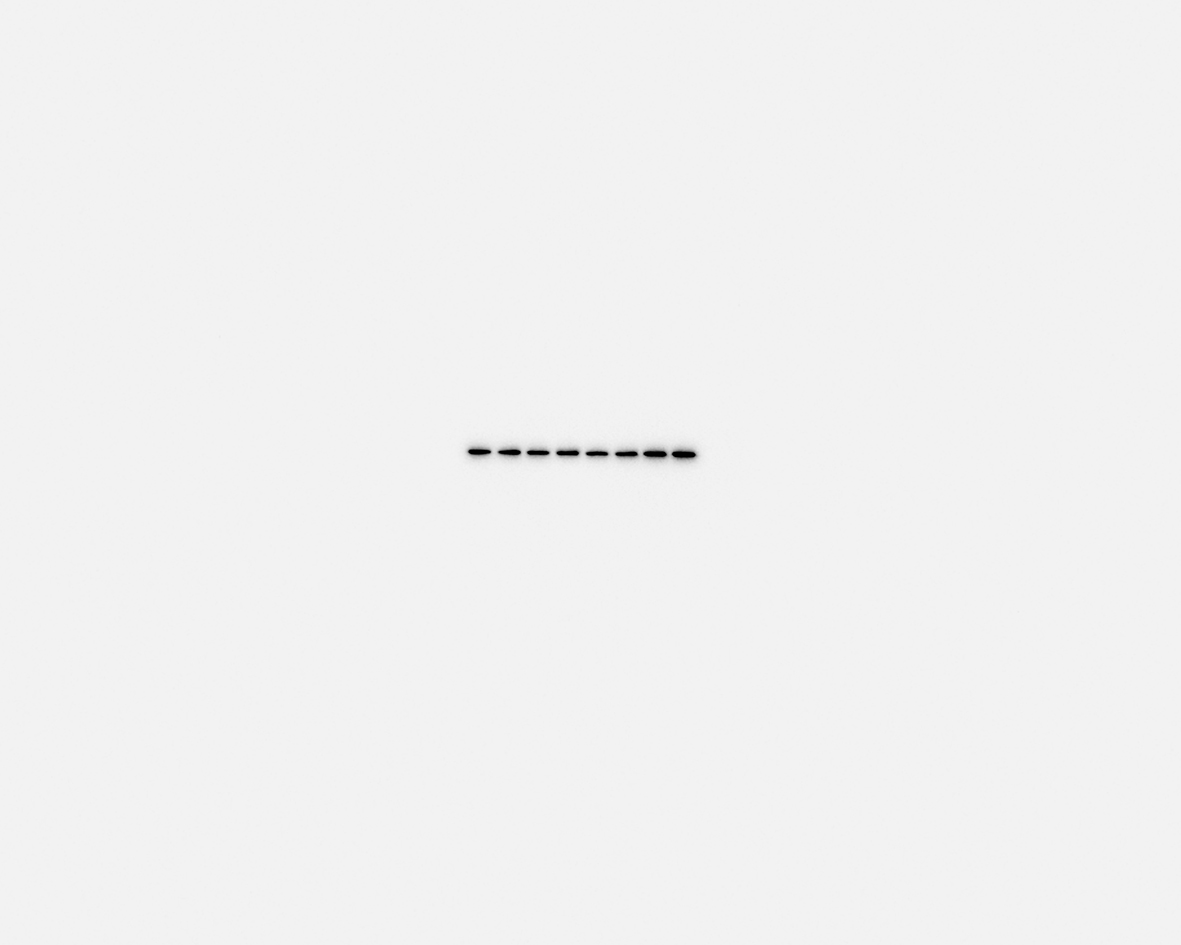

Supplement: Supplementary file 1 [file DataSheet1.ZIP › Figure 3/C-D/GAPDH.jpg]

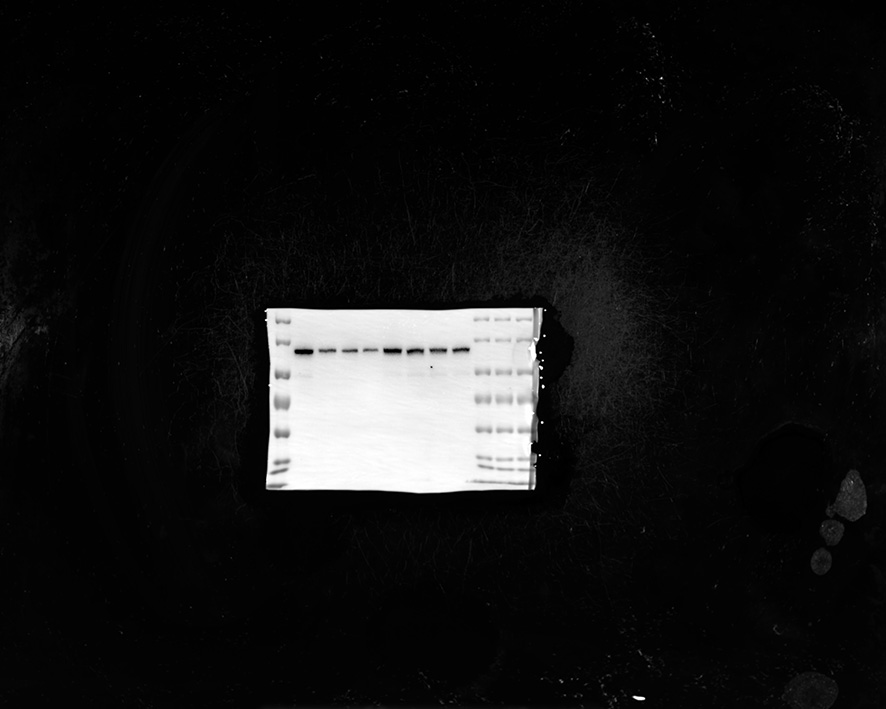

Supplement: Supplementary file 1 [file DataSheet1.ZIP › Figure 3/C-D/NLPR3-1.jpg]

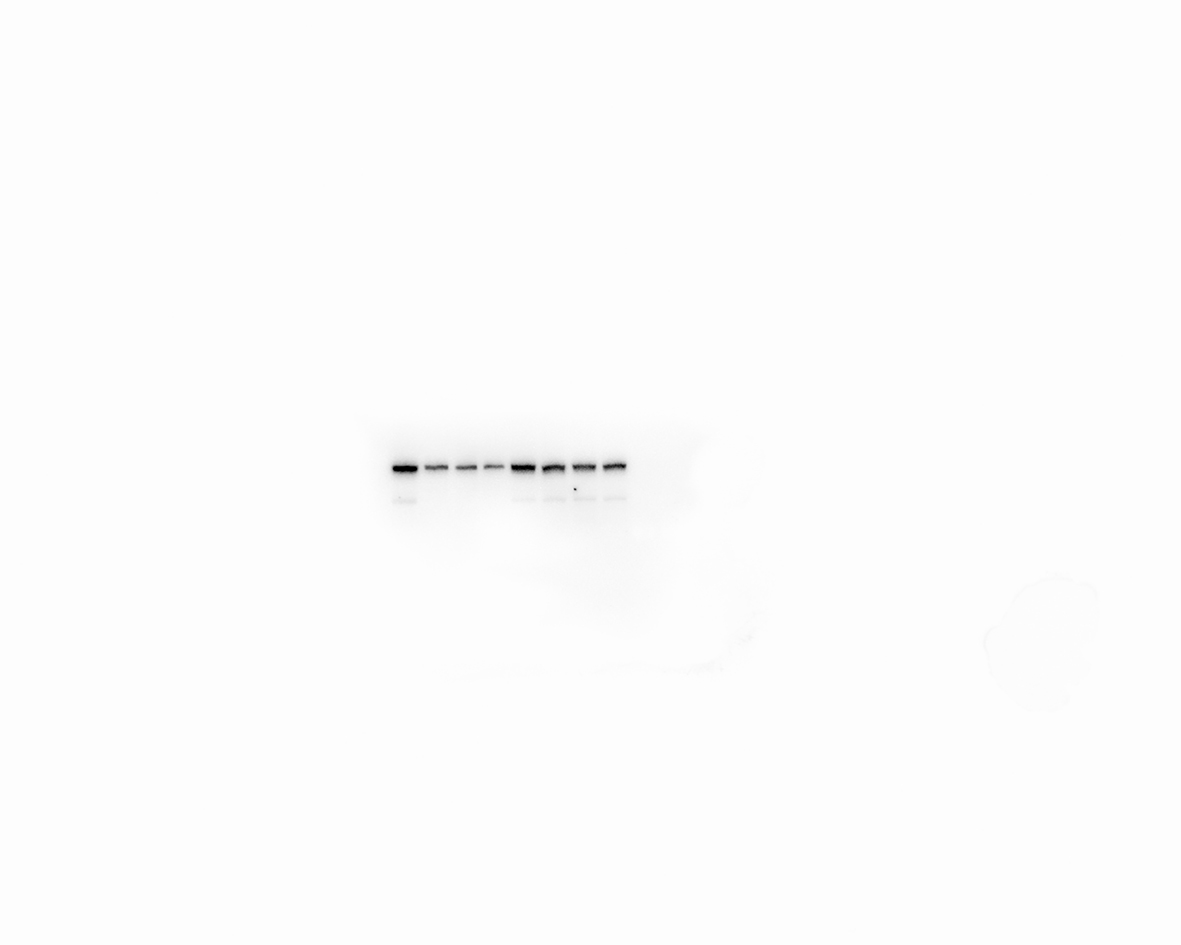

Supplement: Supplementary file 1 [file DataSheet1.ZIP › Figure 3/C-D/NLPR3.jpg]

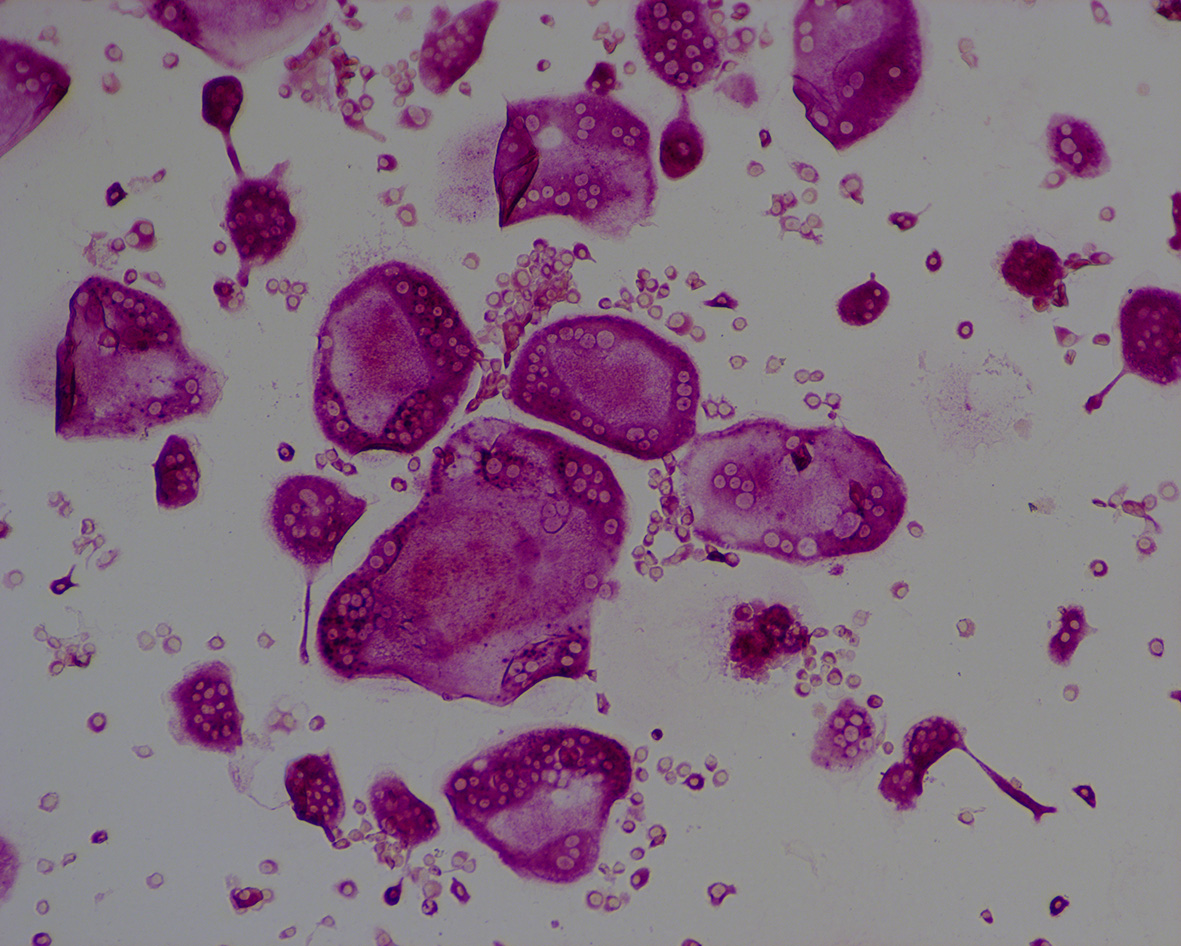

Supplement: Supplementary file 1 [file DataSheet1.ZIP › Figure 1/B-C/0.5.jpg]

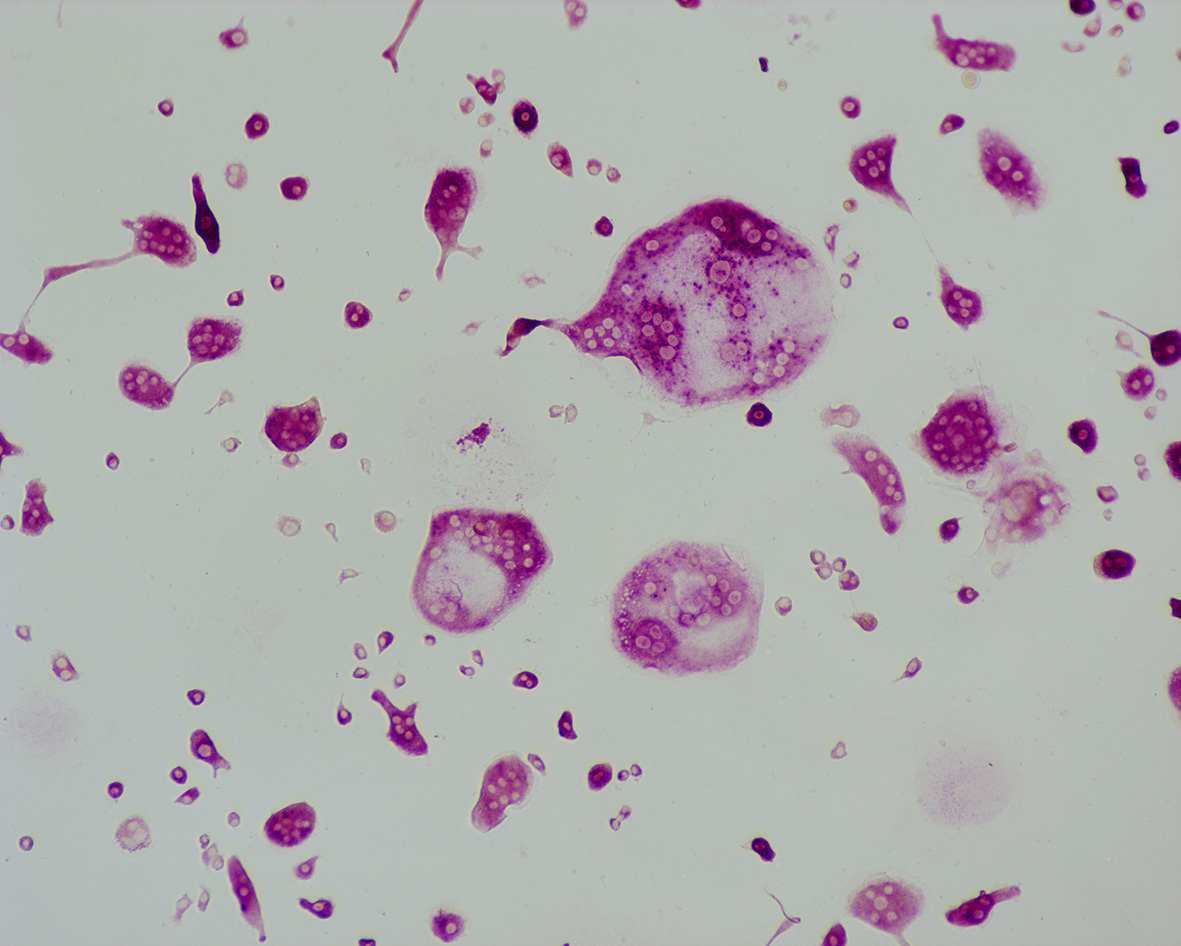

Supplement: Supplementary file 1 [file DataSheet1.ZIP › Figure 1/B-C/1.jpg]

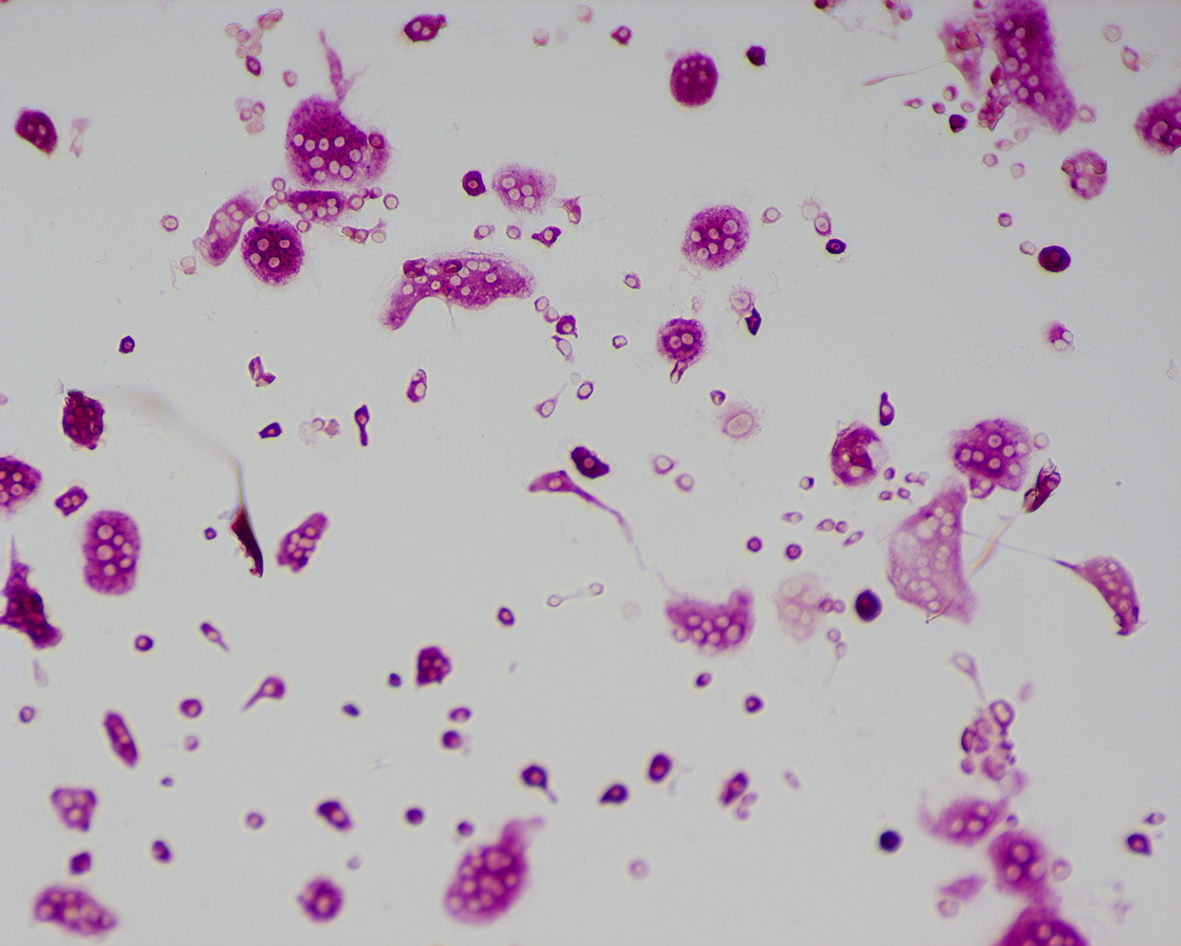

Supplement: Supplementary file 1 [file DataSheet1.ZIP › Figure 1/B-C/2.jpg]

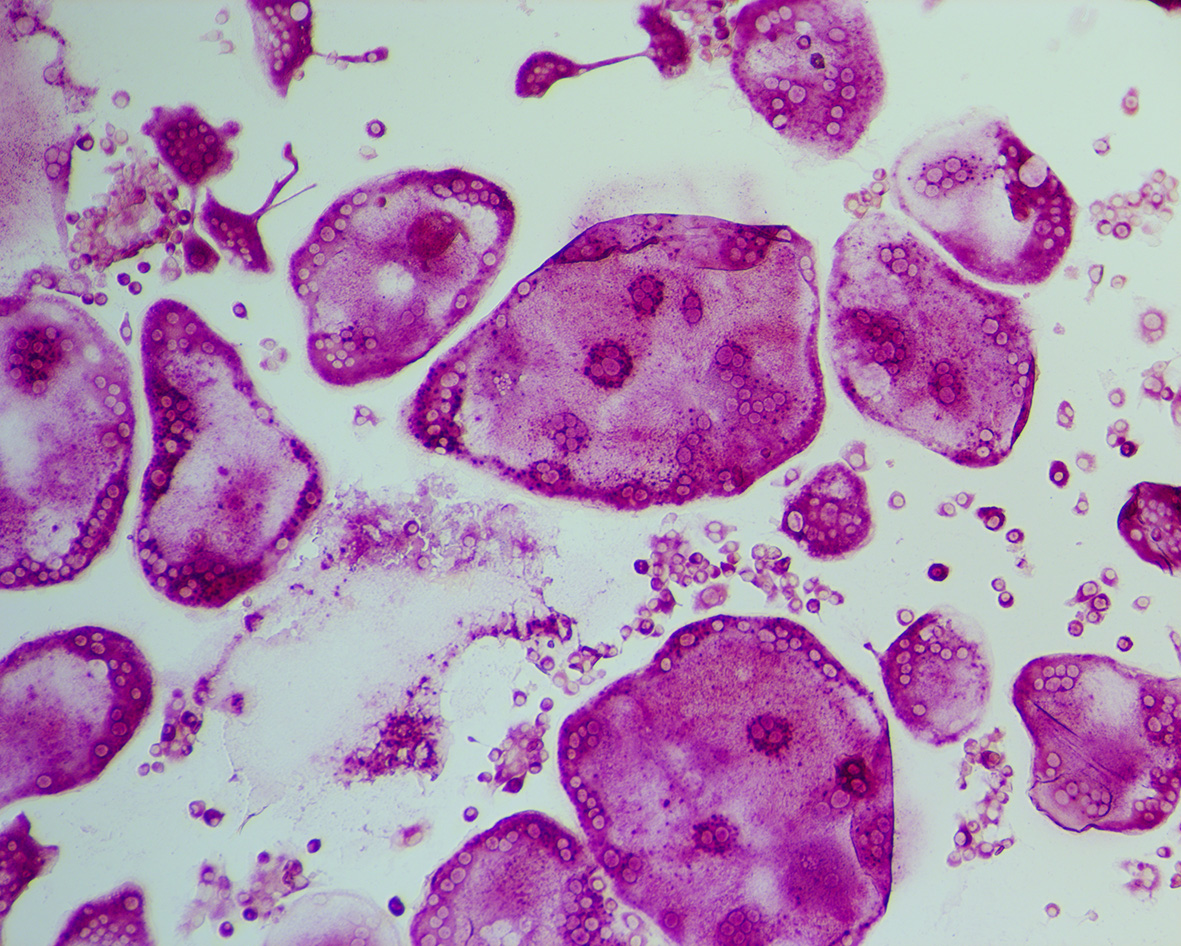

Supplement: Supplementary file 1 [file DataSheet1.ZIP › Figure 1/B-C/DMSO.jpg]

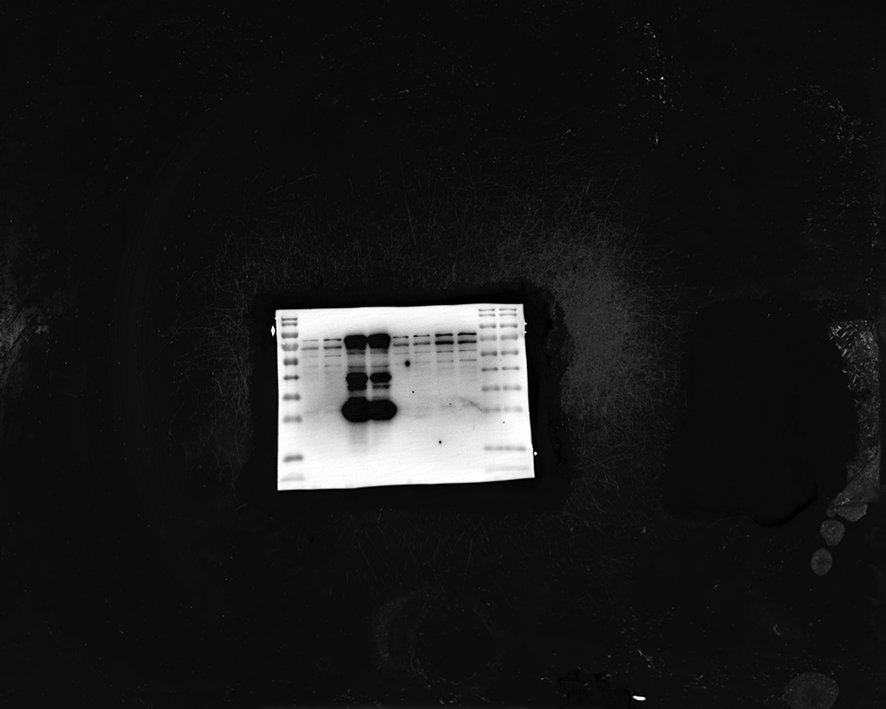

Supplement: Supplementary file 1 [file DataSheet1.ZIP › Figure 1/J/Cathepsin k.jpg]

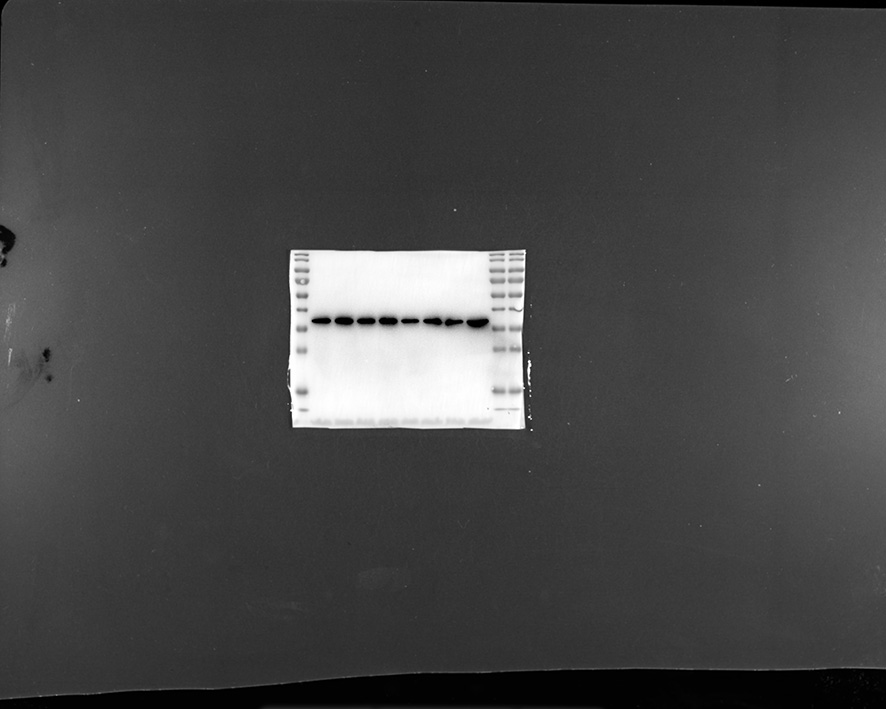

Supplement: Supplementary file 1 [file DataSheet1.ZIP › Figure 1/J/GAPDH 1.jpg]

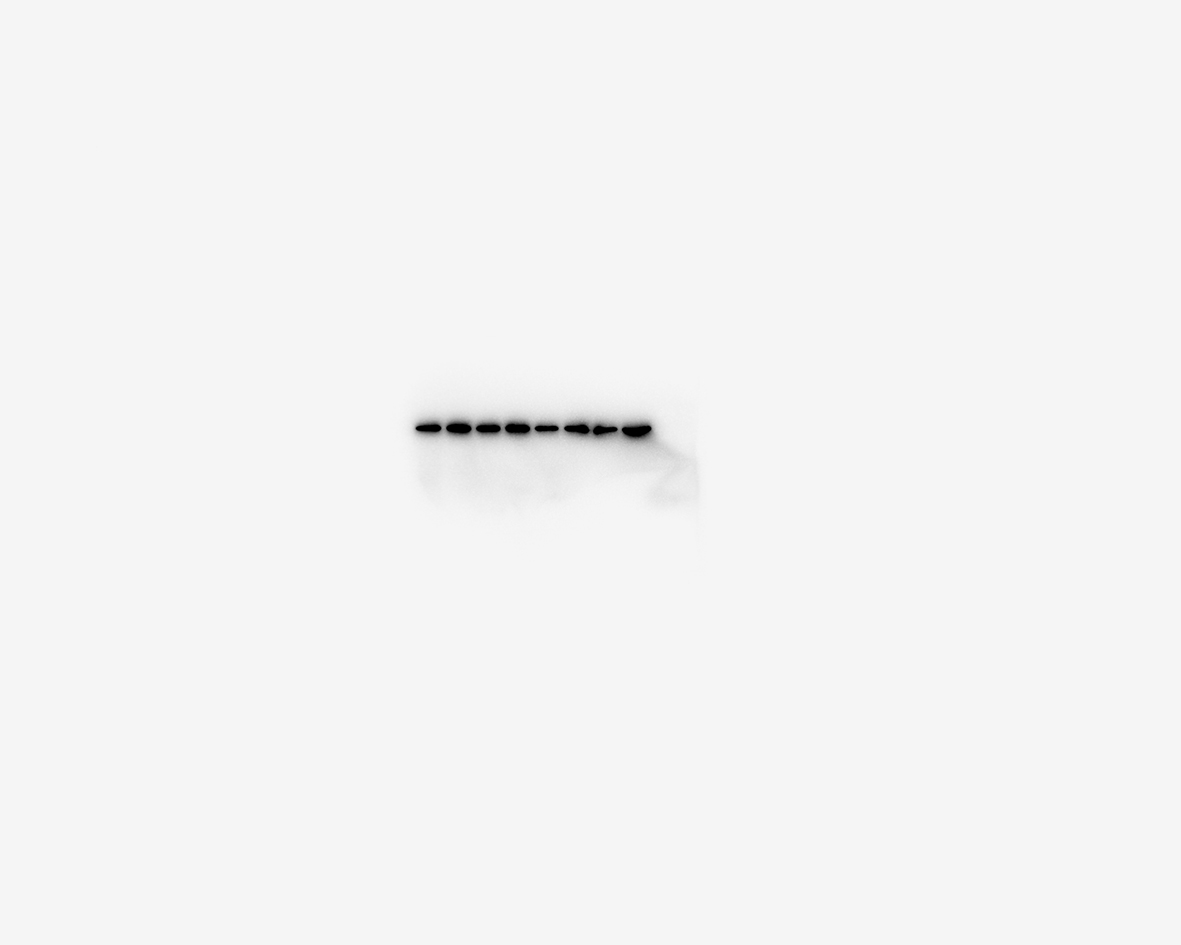

Supplement: Supplementary file 1 [file DataSheet1.ZIP › Figure 1/J/gapdh.jpg]

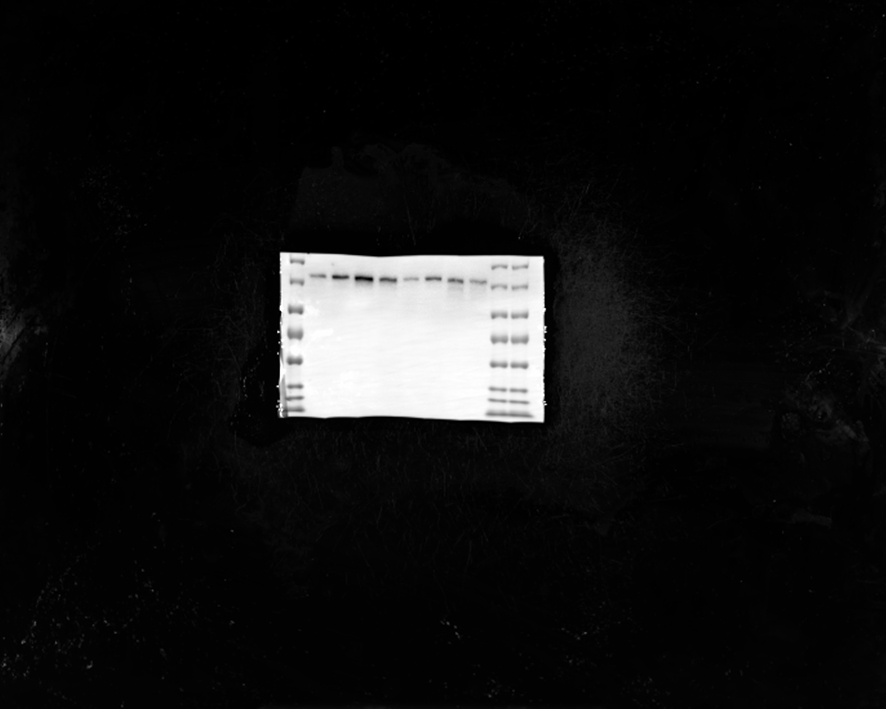

Supplement: Supplementary file 1 [file DataSheet1.ZIP › Figure 1/J/NFATc1-1.jpg]

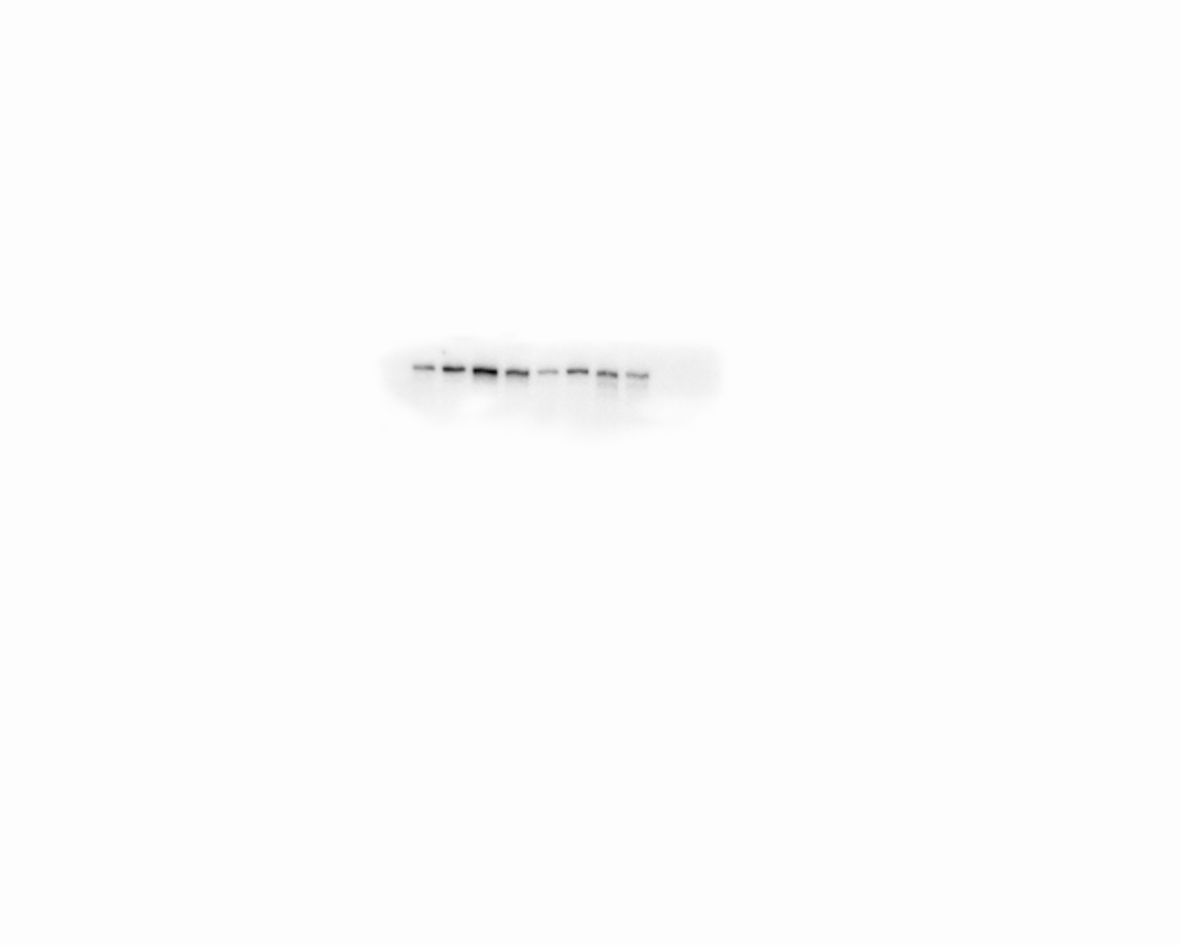

Supplement: Supplementary file 1 [file DataSheet1.ZIP › Figure 1/J/NFATC1.jpg]

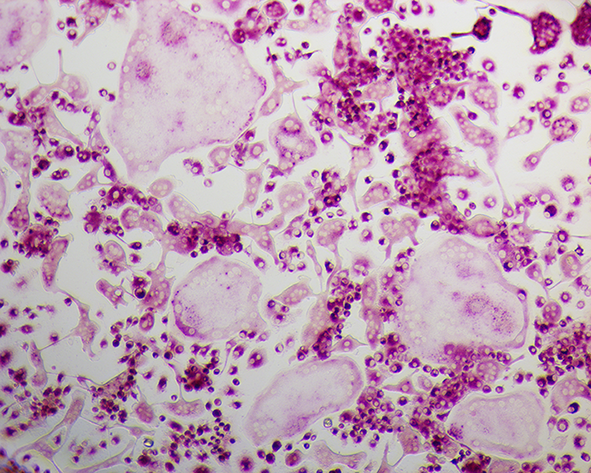

Supplement: Supplementary file 1 [file DataSheet1.ZIP › Figure 2/A-B/DMSO.tif]

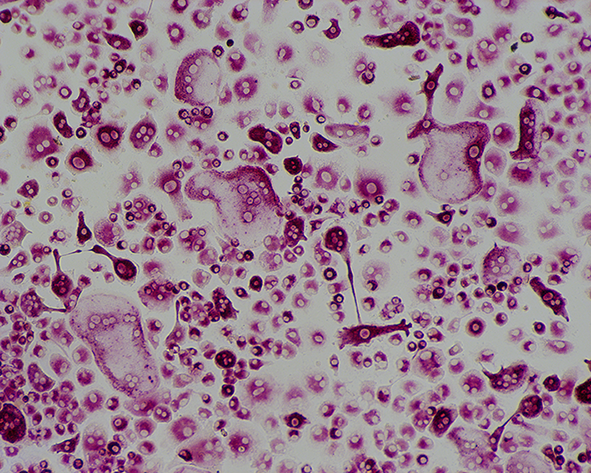

Supplement: Supplementary file 1 [file DataSheet1.ZIP › Figure 2/A-B/EARLY.tif]

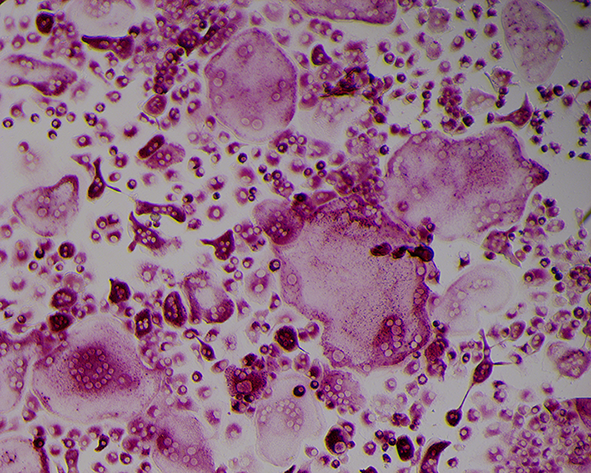

Supplement: Supplementary file 1 [file DataSheet1.ZIP › Figure 2/A-B/LATE.tif]

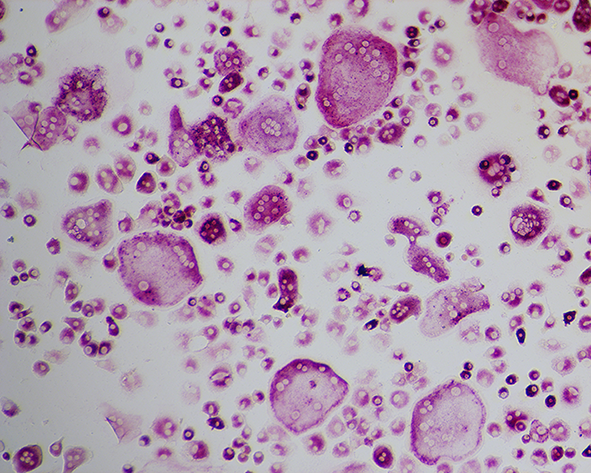

Supplement: Supplementary file 1 [file DataSheet1.ZIP › Figure 2/A-B/MIDDLE.tif]

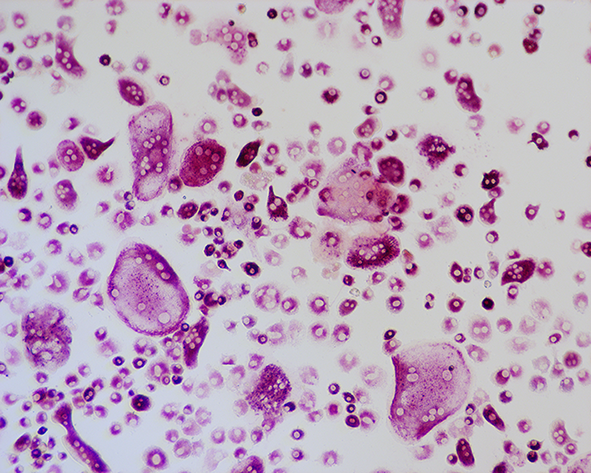

Supplement: Supplementary file 1 [file DataSheet1.ZIP › Figure 2/A-B/WHOLE.tif]

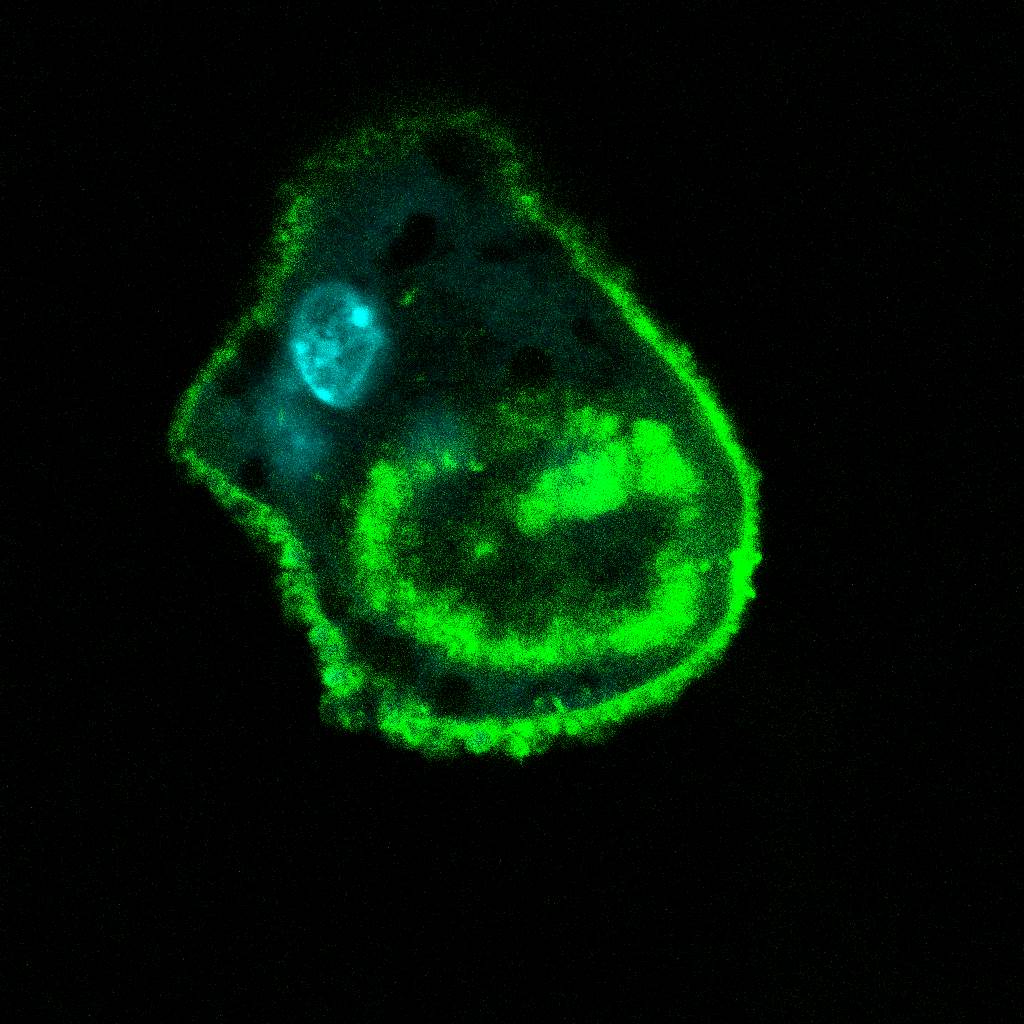

Supplement: Supplementary file 1 [file DataSheet1.ZIP › Figure 2/C-D/0.5-1.jpg]

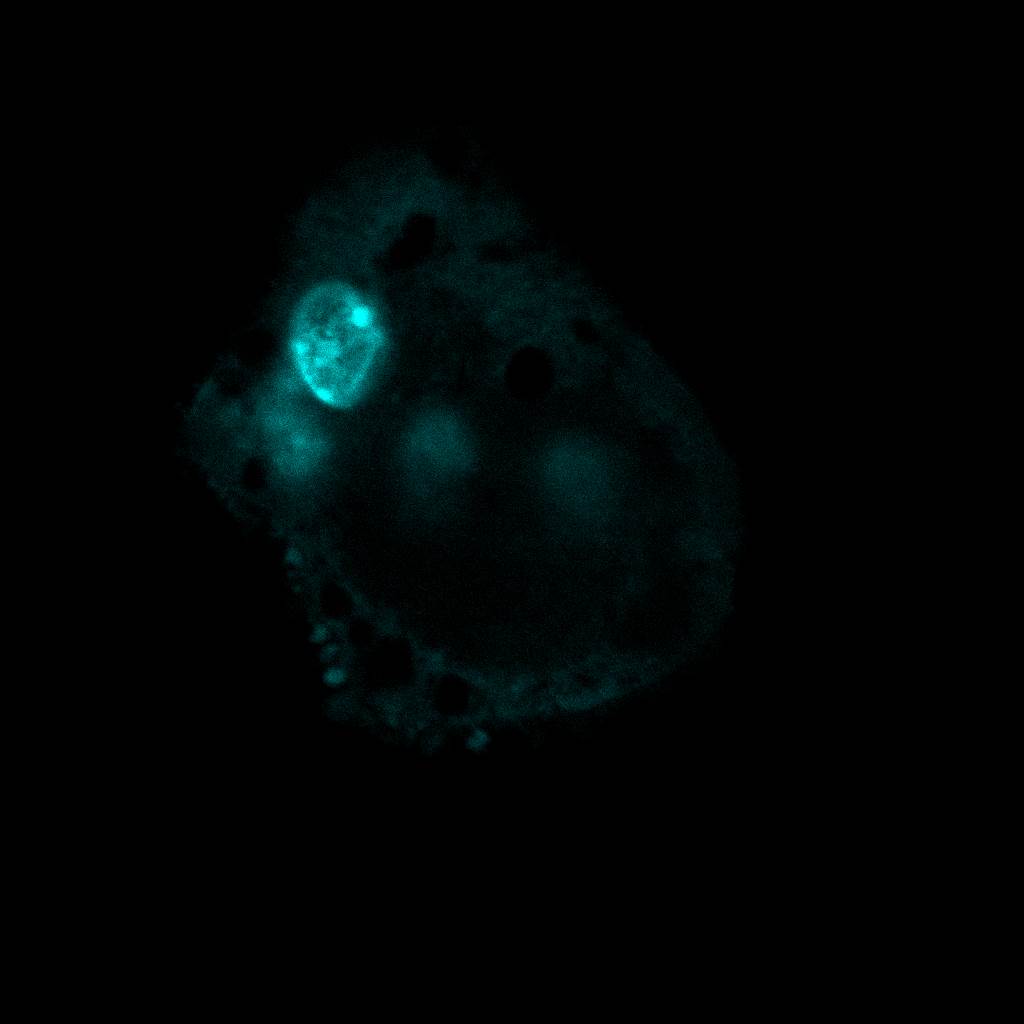

Supplement: Supplementary file 1 [file DataSheet1.ZIP › Figure 2/C-D/0.5-11.jpg]

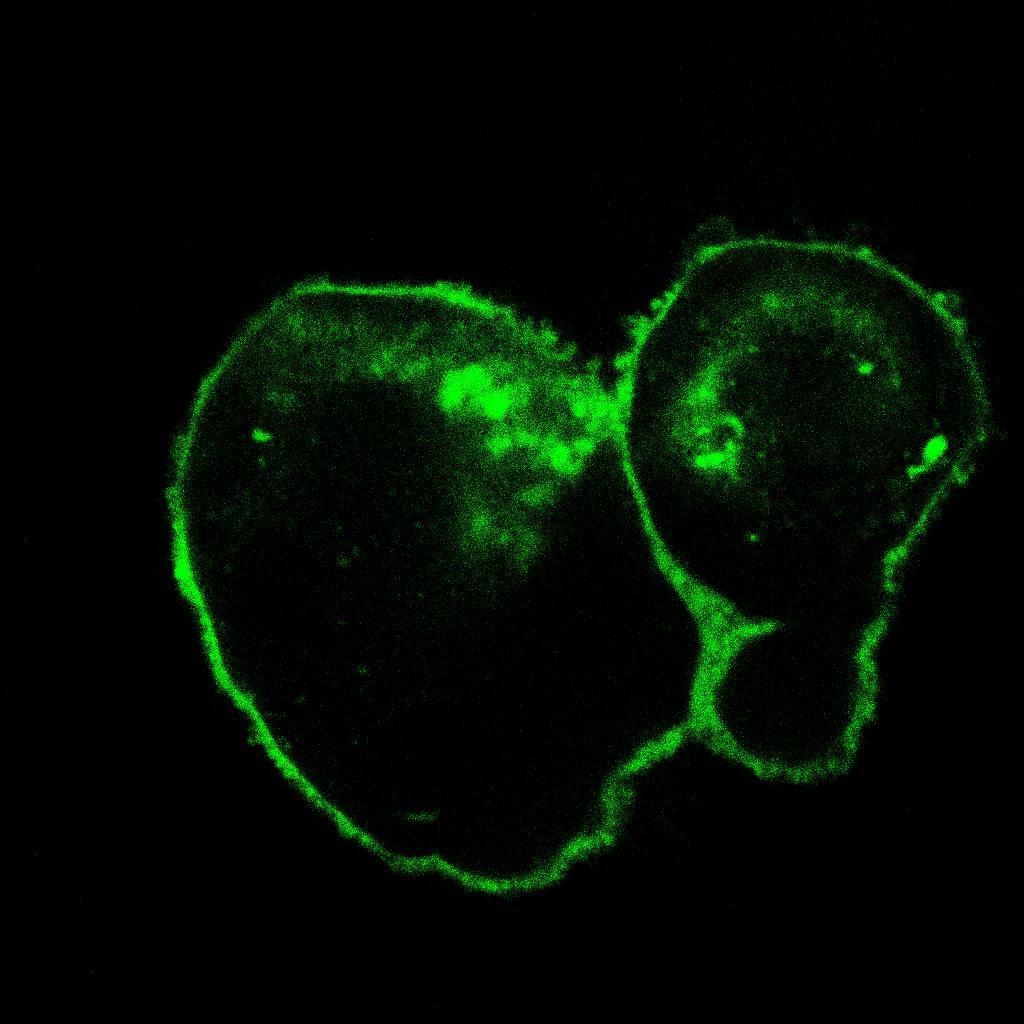

Supplement: Supplementary file 1 [file DataSheet1.ZIP › Figure 2/C-D/1-1.jpg]

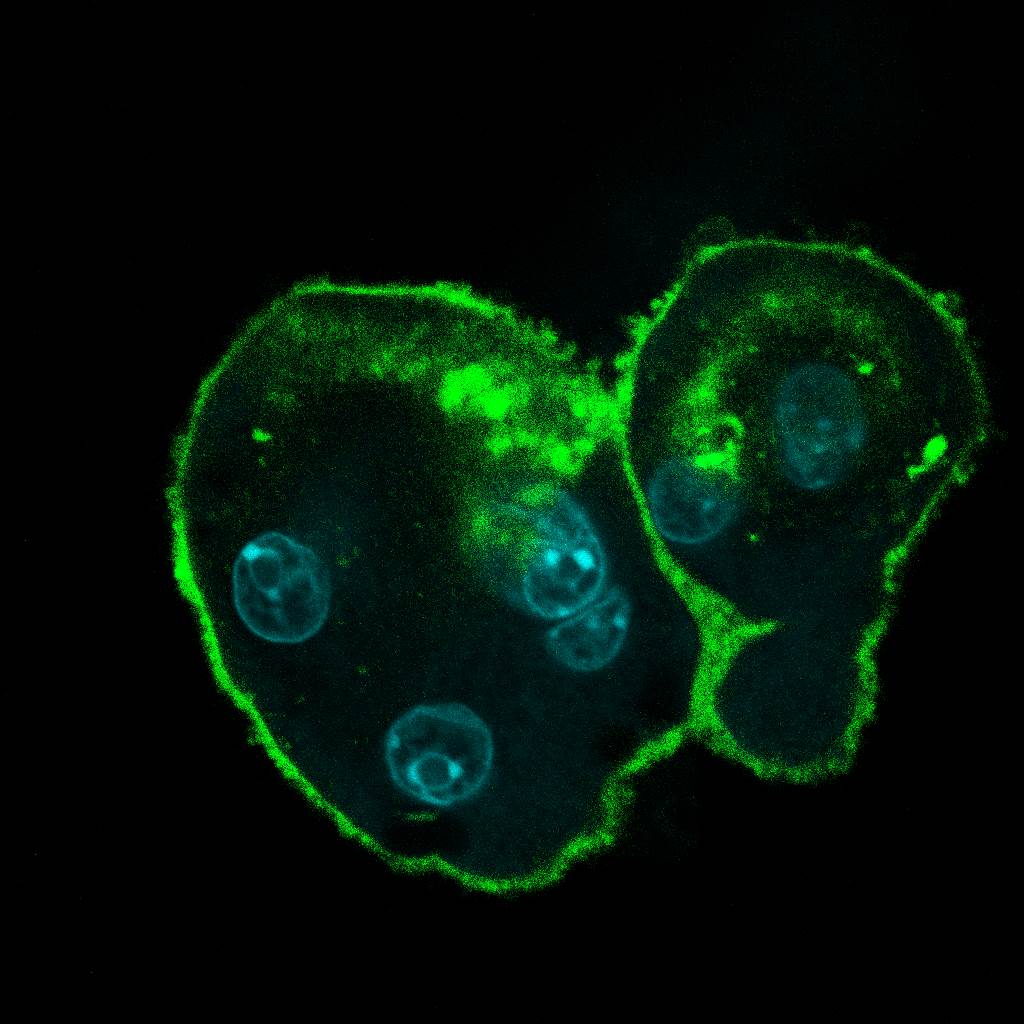

Supplement: Supplementary file 1 [file DataSheet1.ZIP › Figure 2/C-D/1-11.jpg]

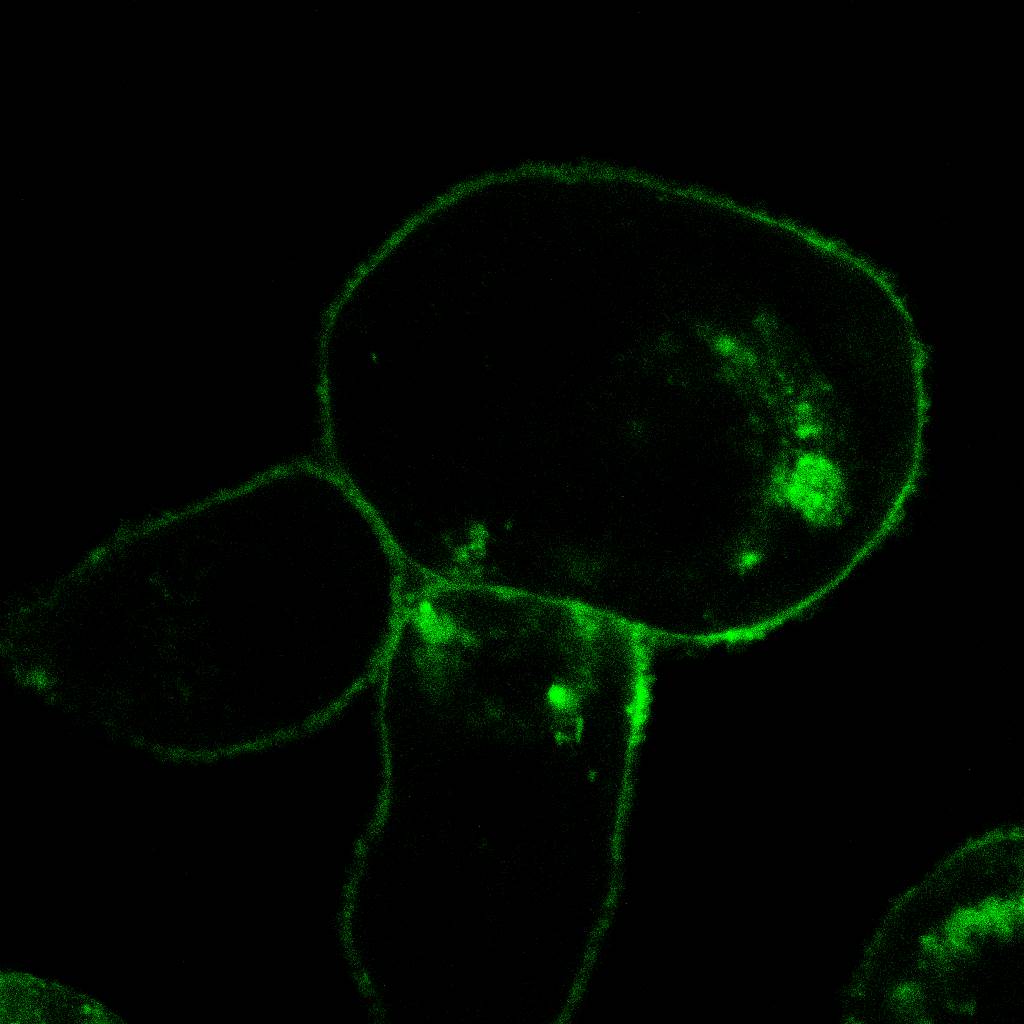

Supplement: Supplementary file 1 [file DataSheet1.ZIP › Figure 2/C-D/2-1.jpg]

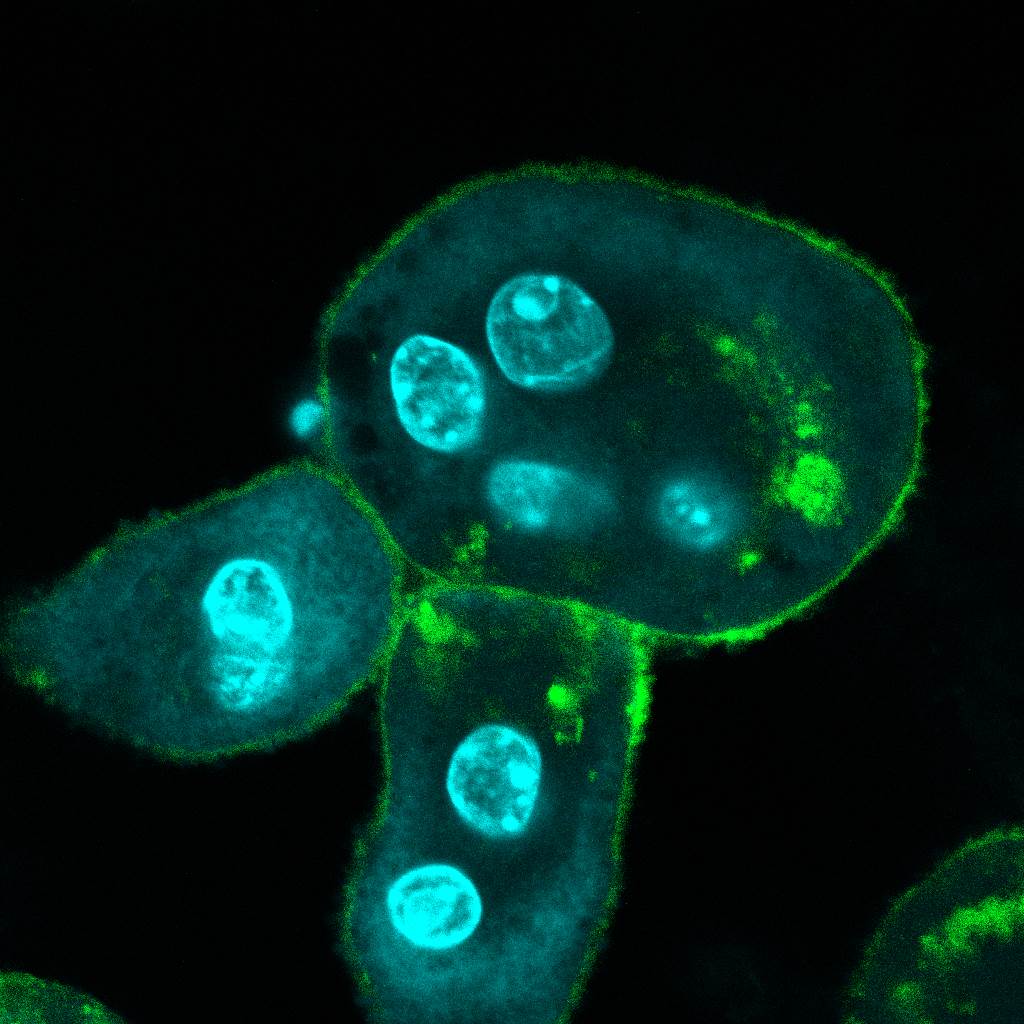

Supplement: Supplementary file 1 [file DataSheet1.ZIP › Figure 2/C-D/2-11.jpg]

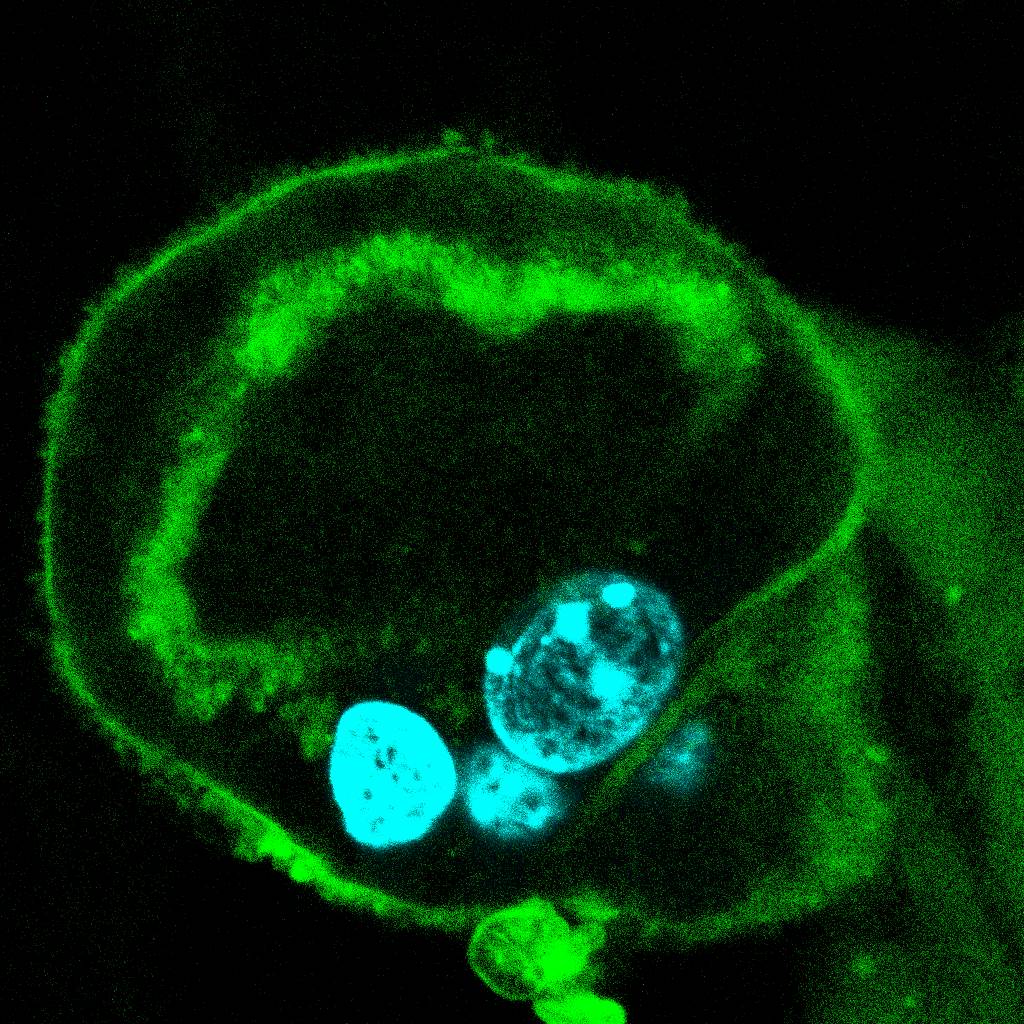

Supplement: Supplementary file 1 [file DataSheet1.ZIP › Figure 2/C-D/DMSO-1.jpg]

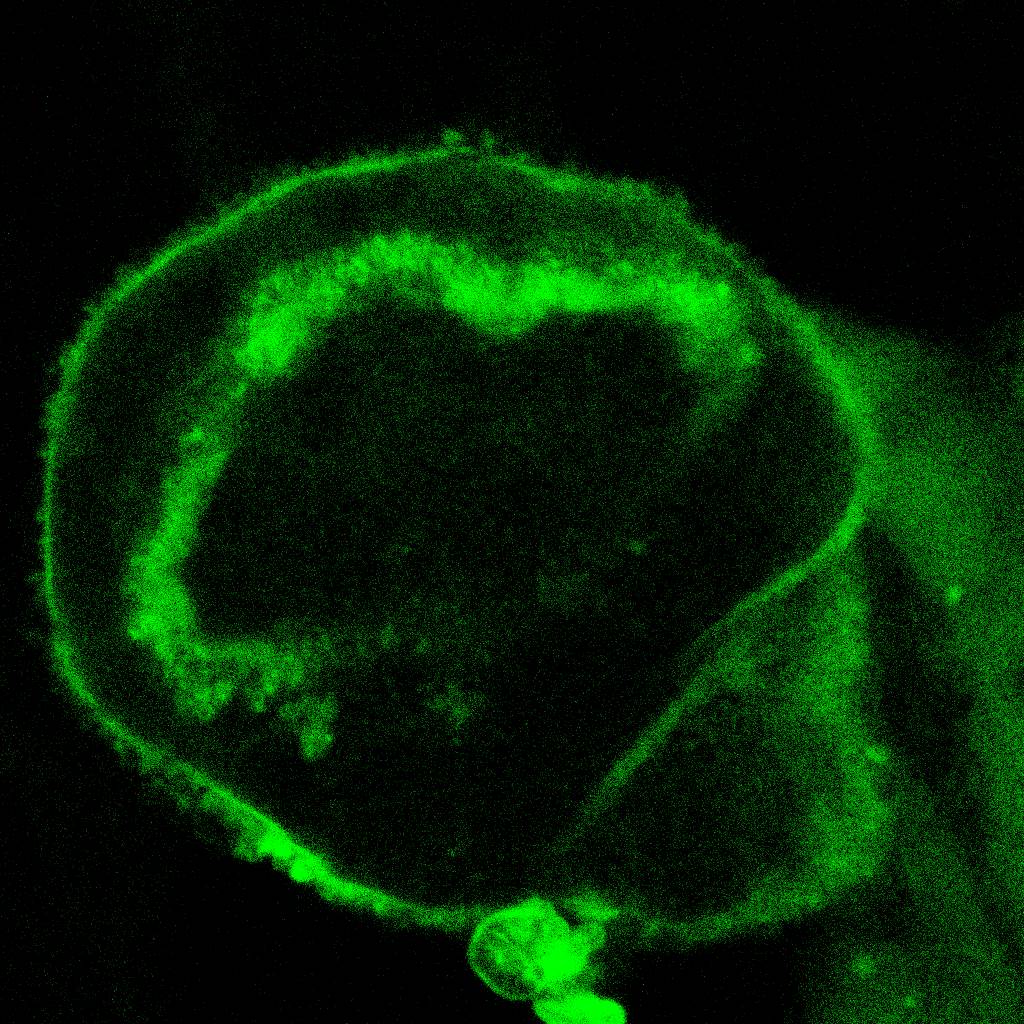

Supplement: Supplementary file 1 [file DataSheet1.ZIP › Figure 2/C-D/DMSO.jpg]

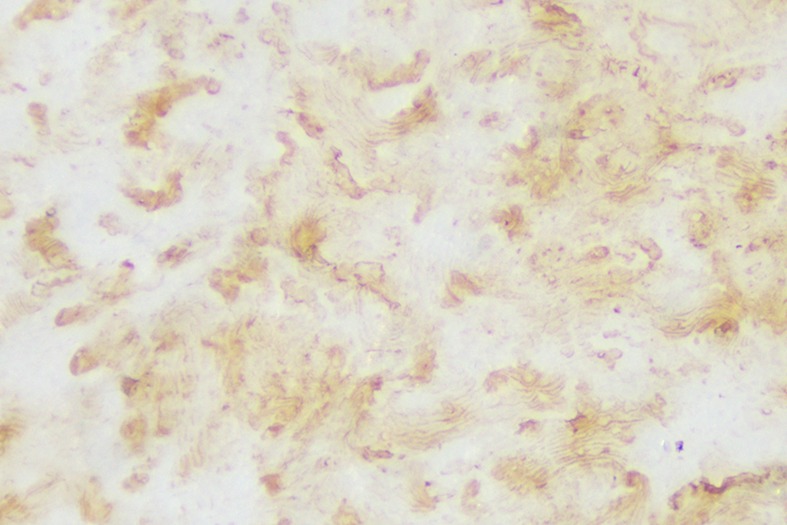

Supplement: Supplementary file 1 [file DataSheet1.ZIP › Figure 2/E-F/0.5.jpg]

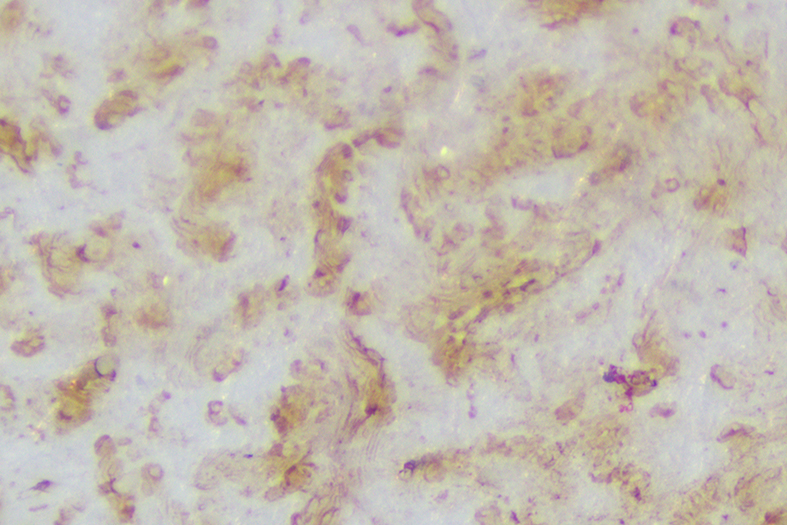

Supplement: Supplementary file 1 [file DataSheet1.ZIP › Figure 2/E-F/1.jpg]

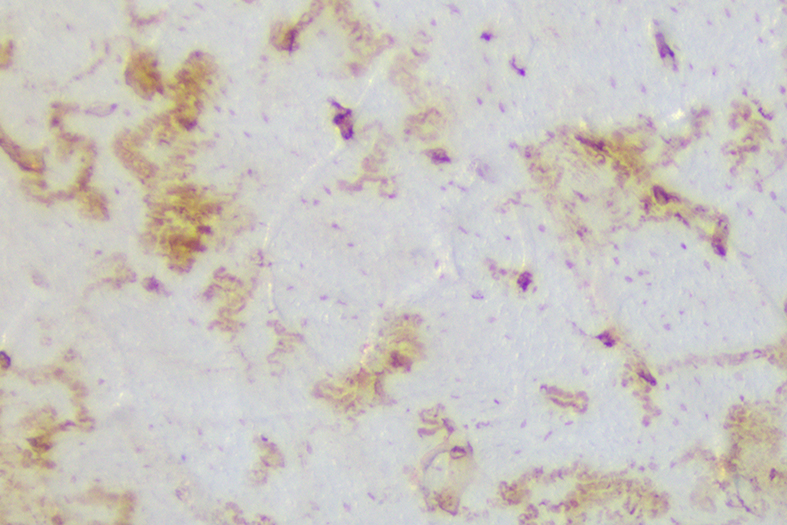

Supplement: Supplementary file 1 [file DataSheet1.ZIP › Figure 2/E-F/2.jpg]

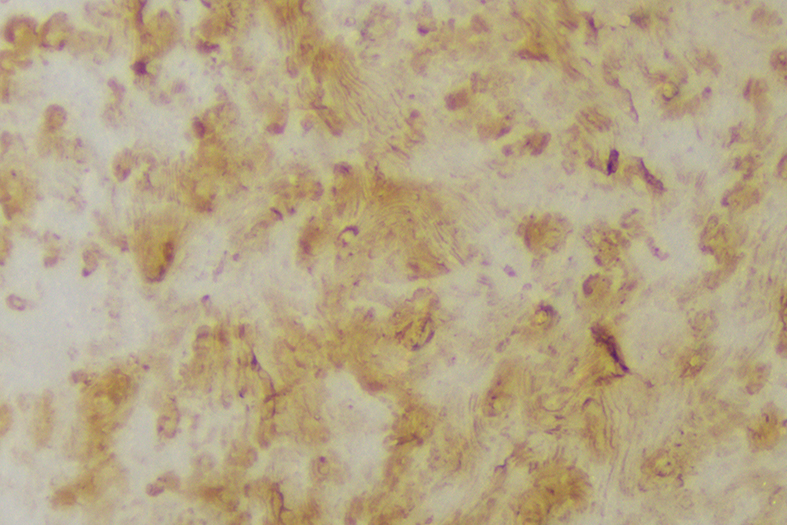

Supplement: Supplementary file 1 [file DataSheet1.ZIP › Figure 2/E-F/dmso.jpg]

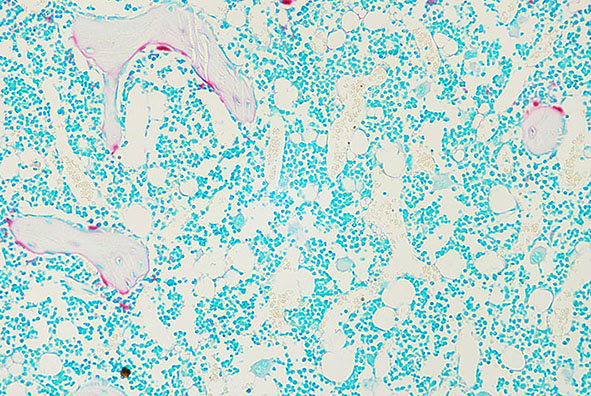

Supplement: Supplementary file 2 [file DataSheet2.ZIP › Figure 6/HD.jpg]

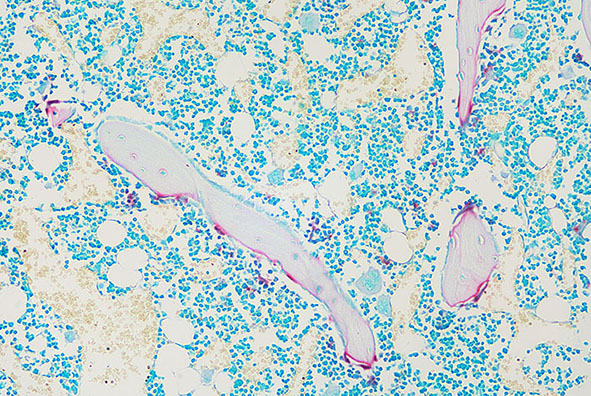

Supplement: Supplementary file 2 [file DataSheet2.ZIP › Figure 6/LD.jpg]

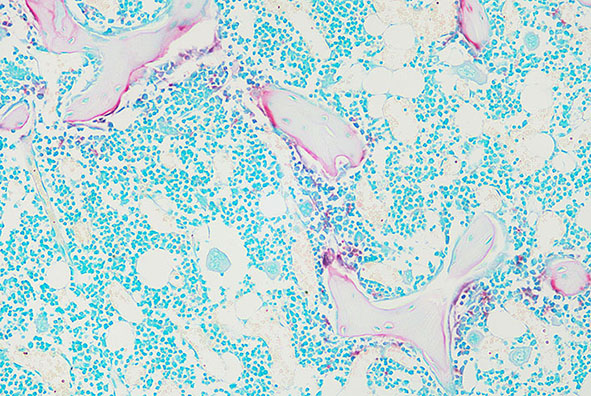

Supplement: Supplementary file 2 [file DataSheet2.ZIP › Figure 6/OVX.jpg]

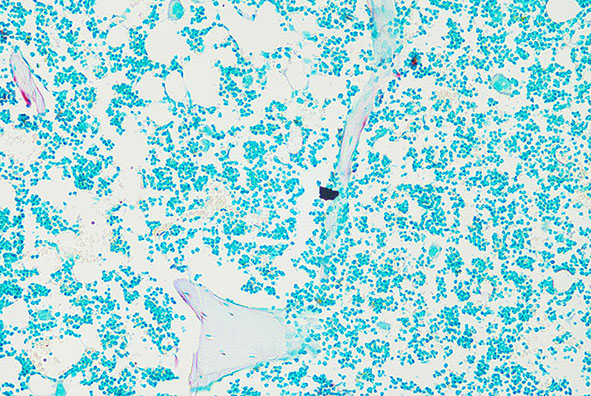

Supplement: Supplementary file 2 [file DataSheet2.ZIP › Figure 6/SHAM.jpg]

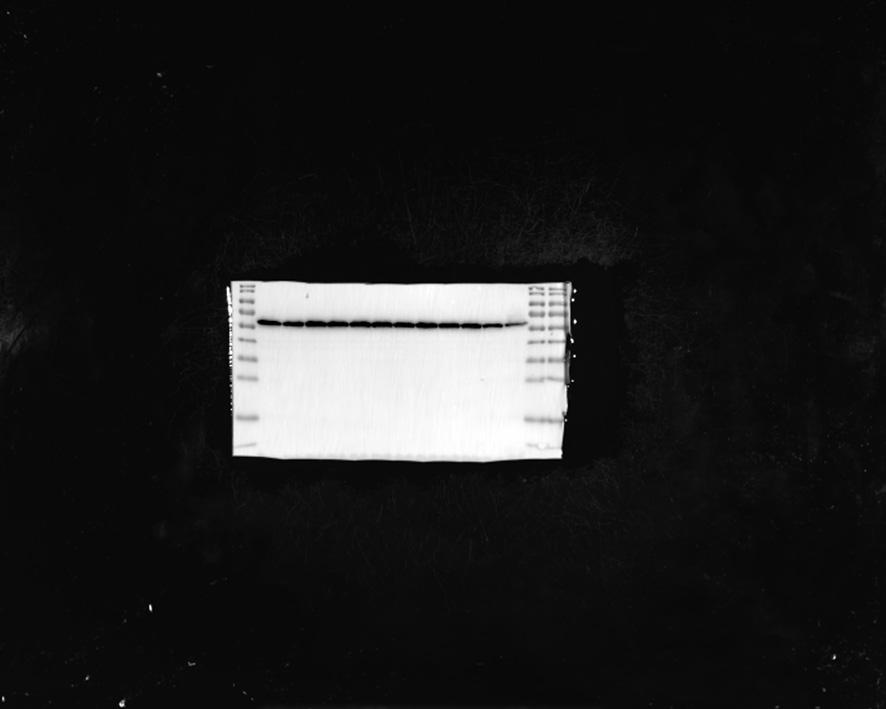

Supplement: Supplementary file 2 [file DataSheet2.ZIP › Figure 4/AKT-1.jpg]

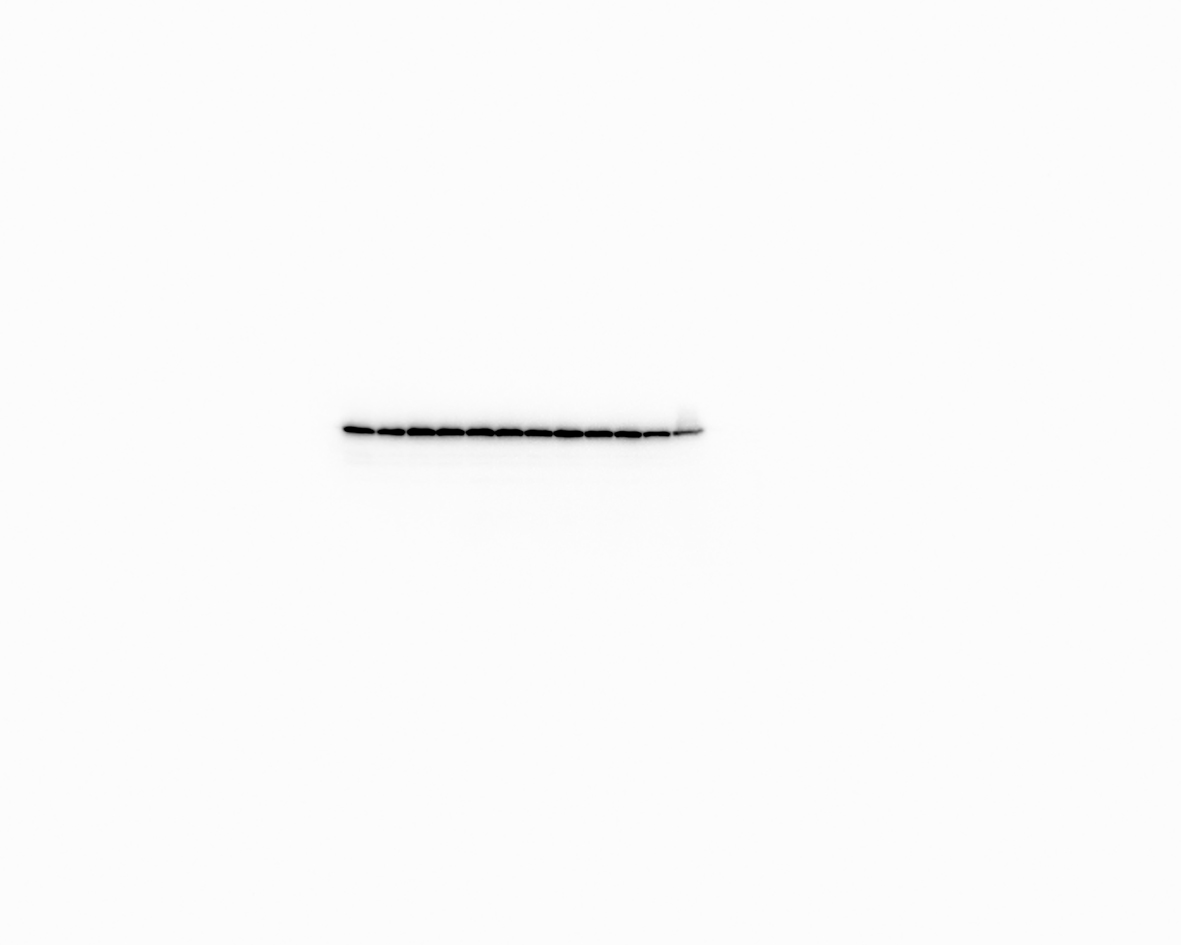

Supplement: Supplementary file 2 [file DataSheet2.ZIP › Figure 4/AKT.jpg]

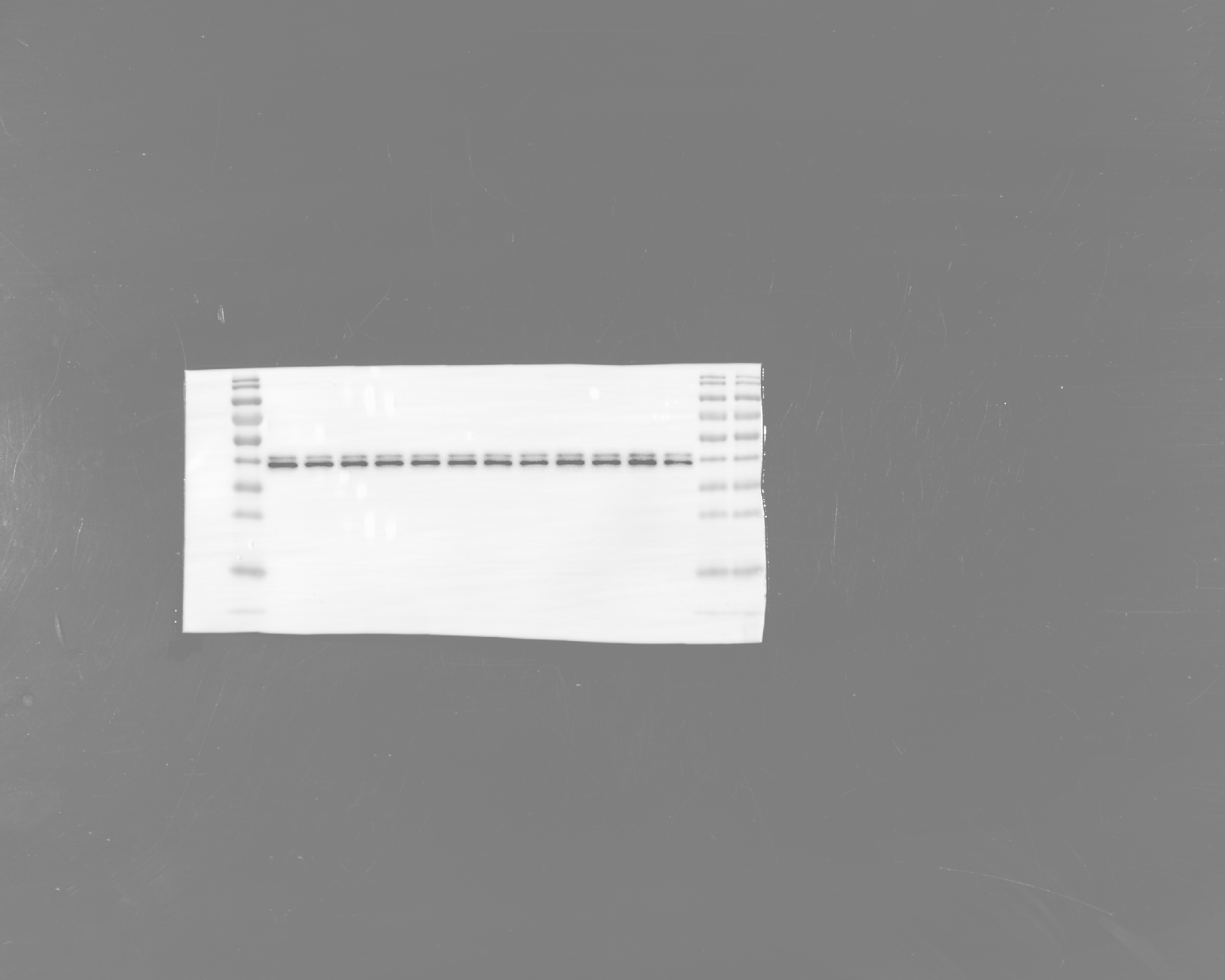

Supplement: Supplementary file 2 [file DataSheet2.ZIP › Figure 4/ERK-1.jpg]

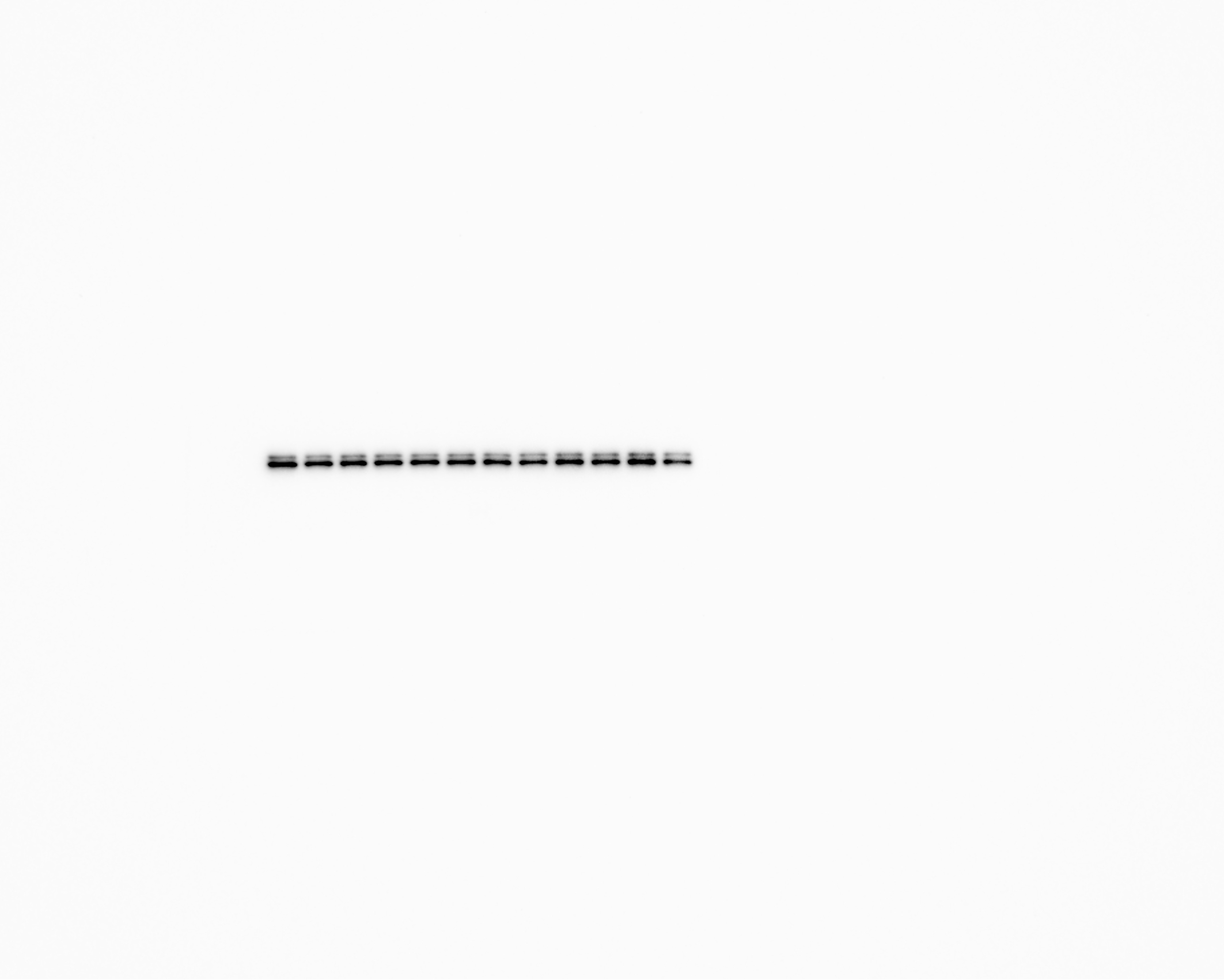

Supplement: Supplementary file 2 [file DataSheet2.ZIP › Figure 4/ERK.jpg]

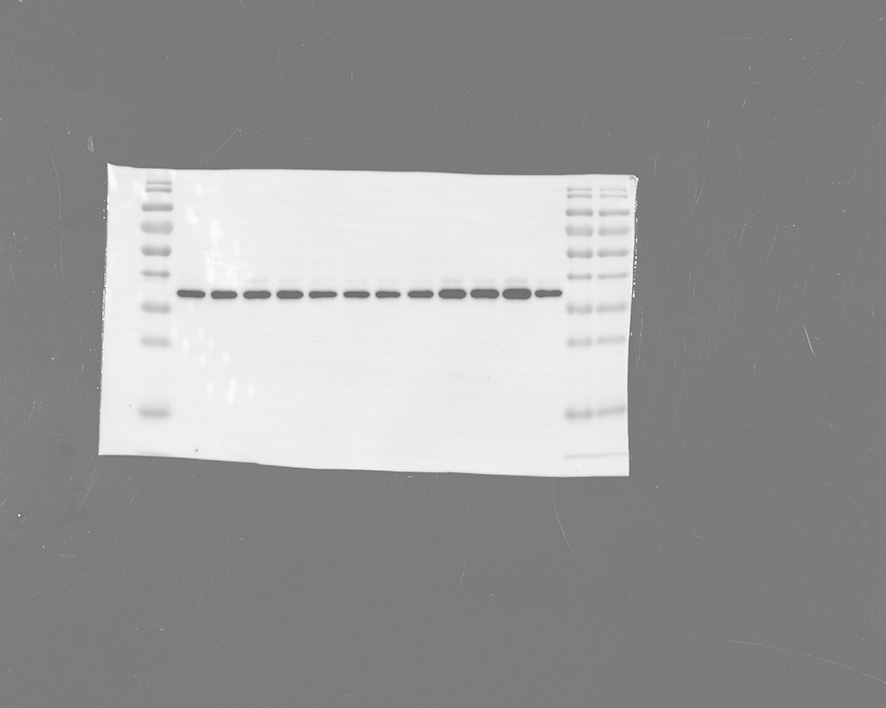

Supplement: Supplementary file 2 [file DataSheet2.ZIP › Figure 4/GAPDH-1.jpg]

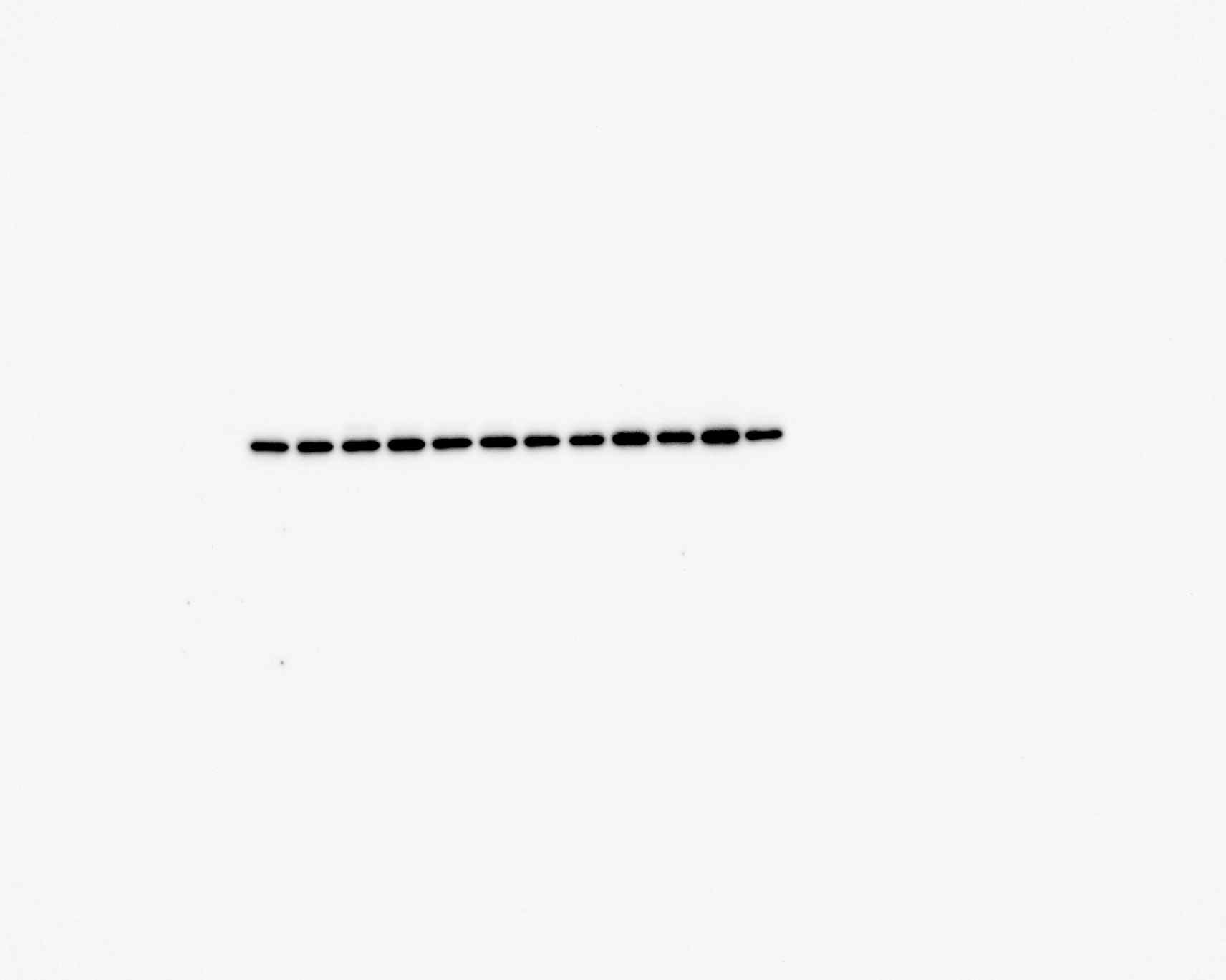

Supplement: Supplementary file 2 [file DataSheet2.ZIP › Figure 4/GAPDH.jpg]

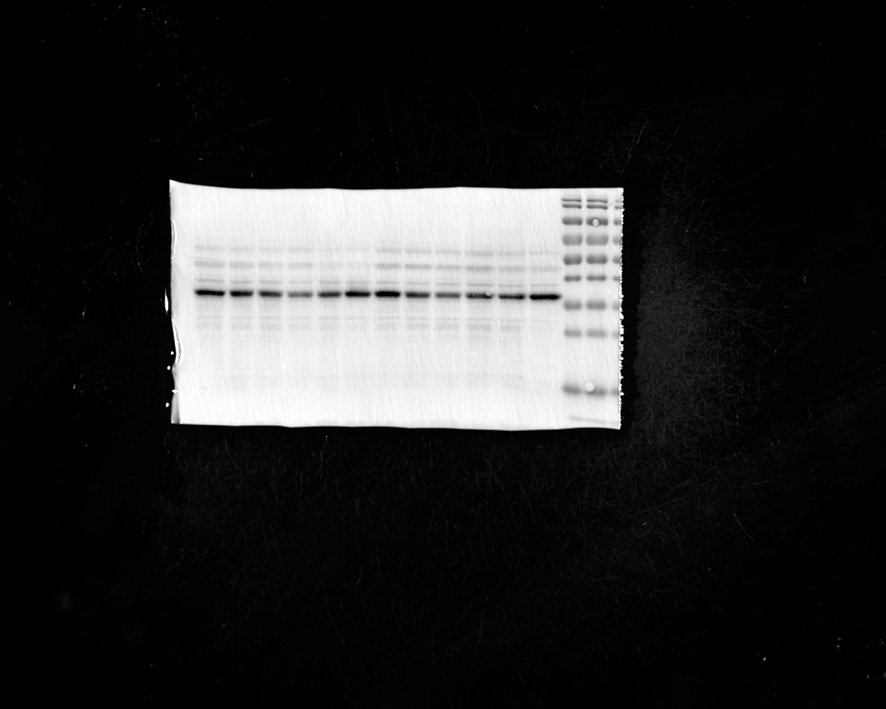

Supplement: Supplementary file 2 [file DataSheet2.ZIP › Figure 4/IKBA-1.jpg]

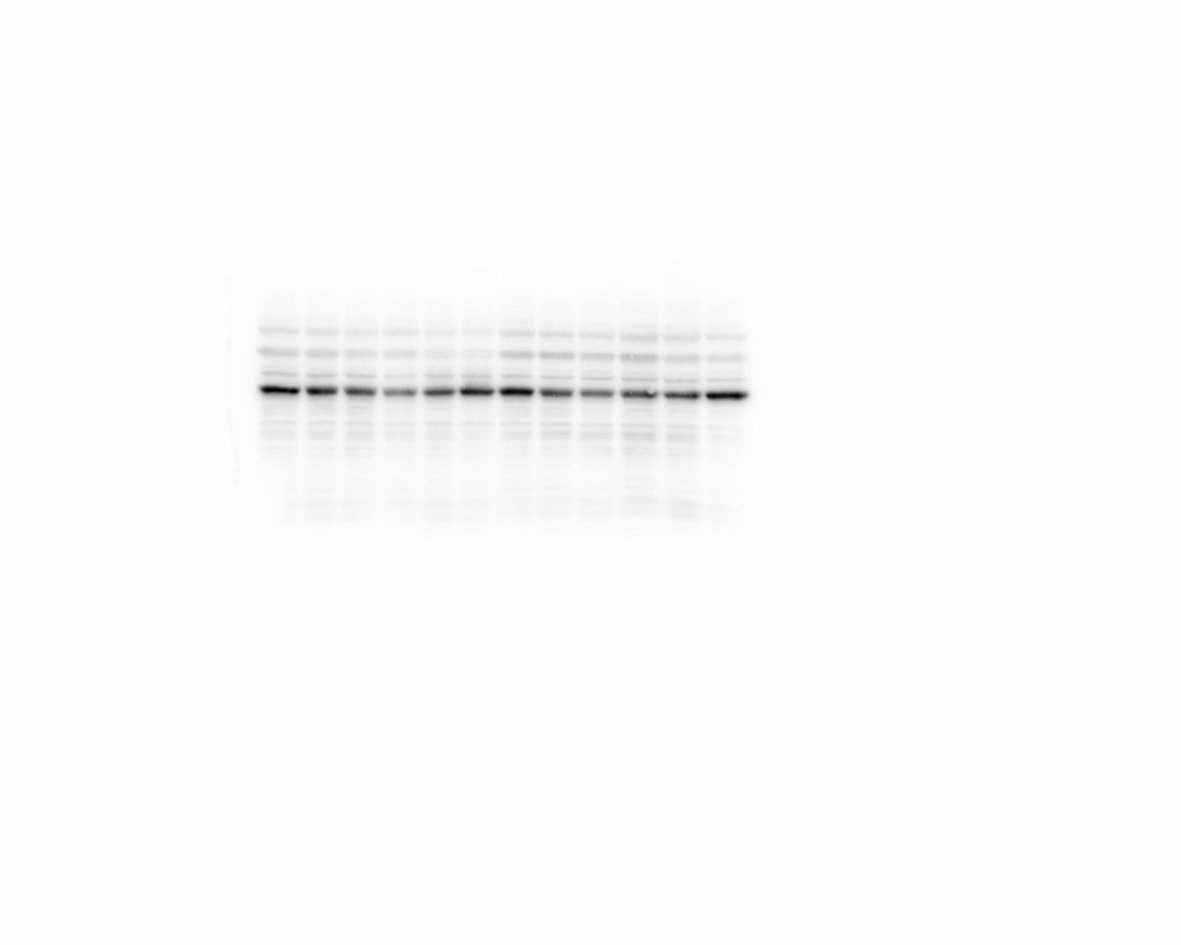

Supplement: Supplementary file 2 [file DataSheet2.ZIP › Figure 4/IKBA.jpg]

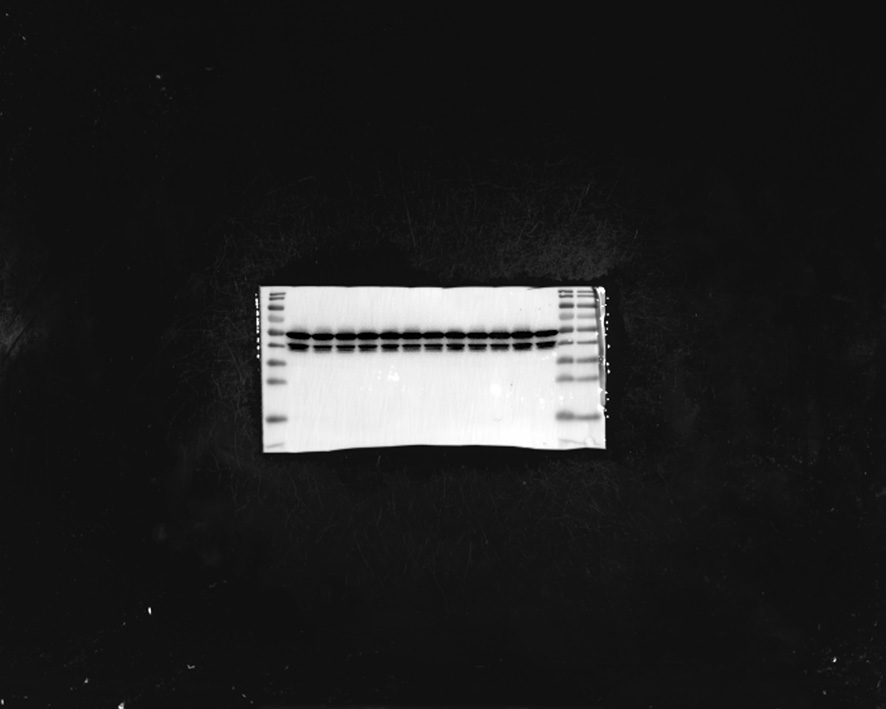

Supplement: Supplementary file 2 [file DataSheet2.ZIP › Figure 4/JNK-1.jpg]

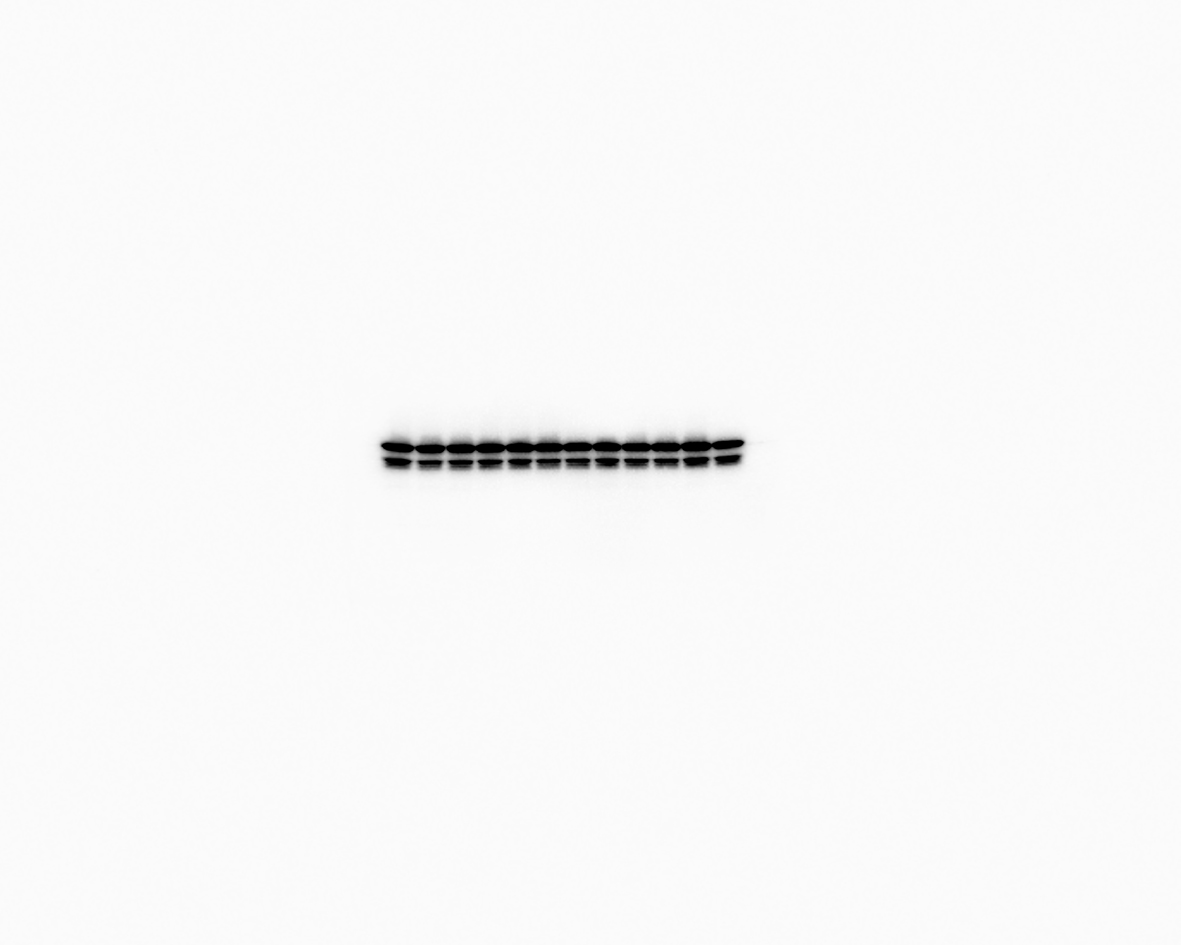

Supplement: Supplementary file 2 [file DataSheet2.ZIP › Figure 4/JNK.jpg]

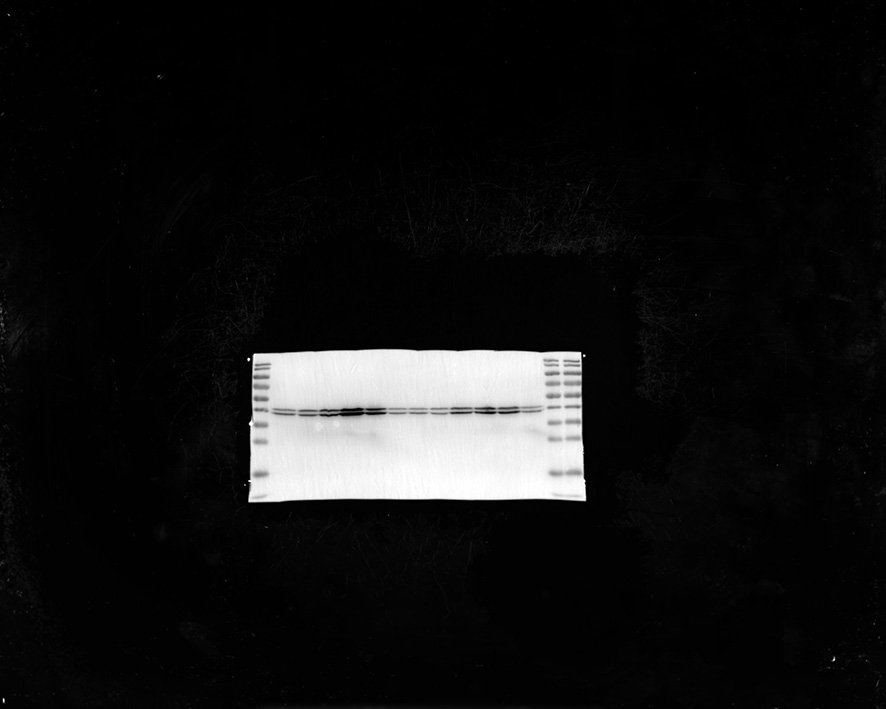

Supplement: Supplementary file 2 [file DataSheet2.ZIP › Figure 4/P-ERK-1.jpg]

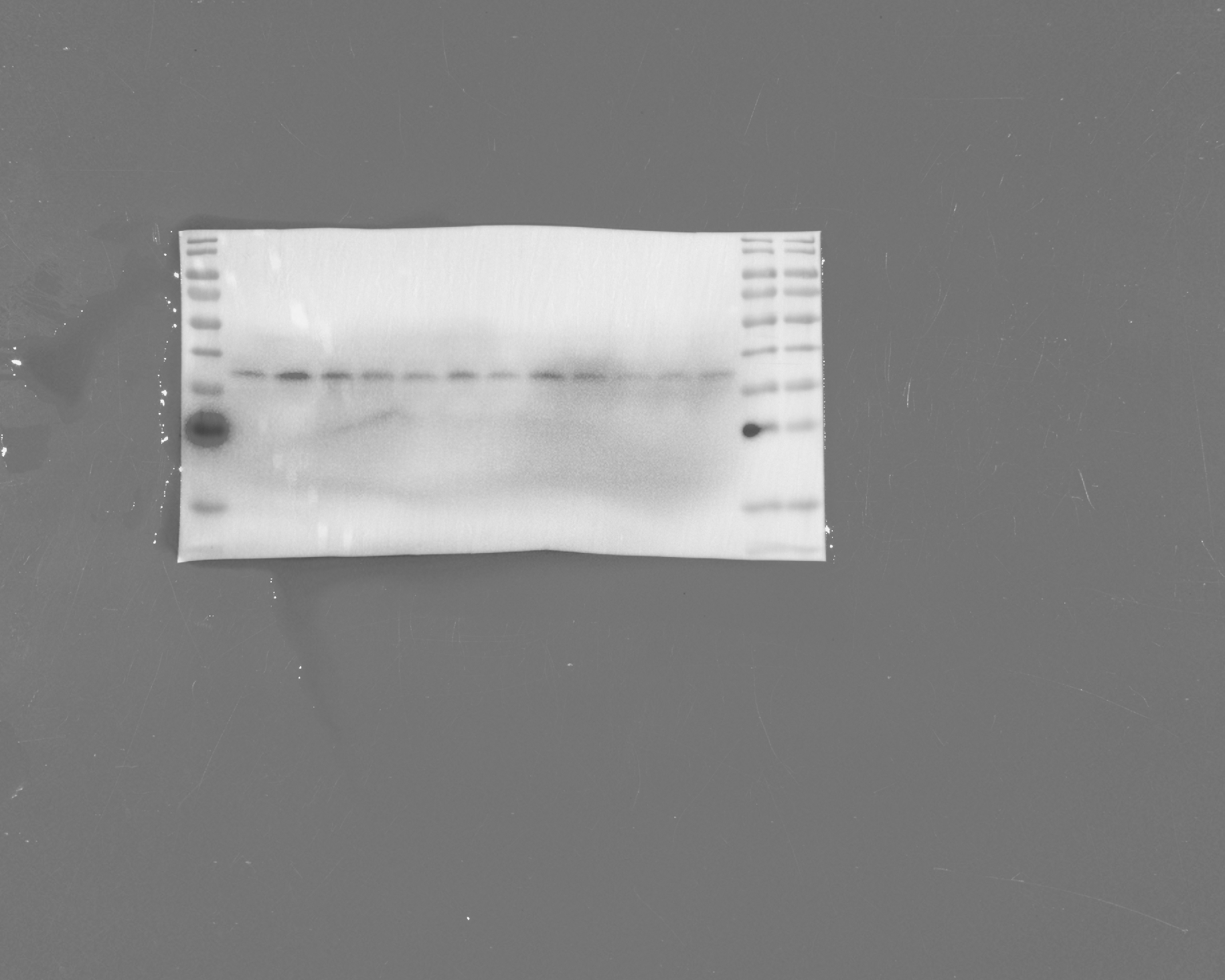

Supplement: Supplementary file 2 [file DataSheet2.ZIP › Figure 4/P-IKBA-1.jpg]

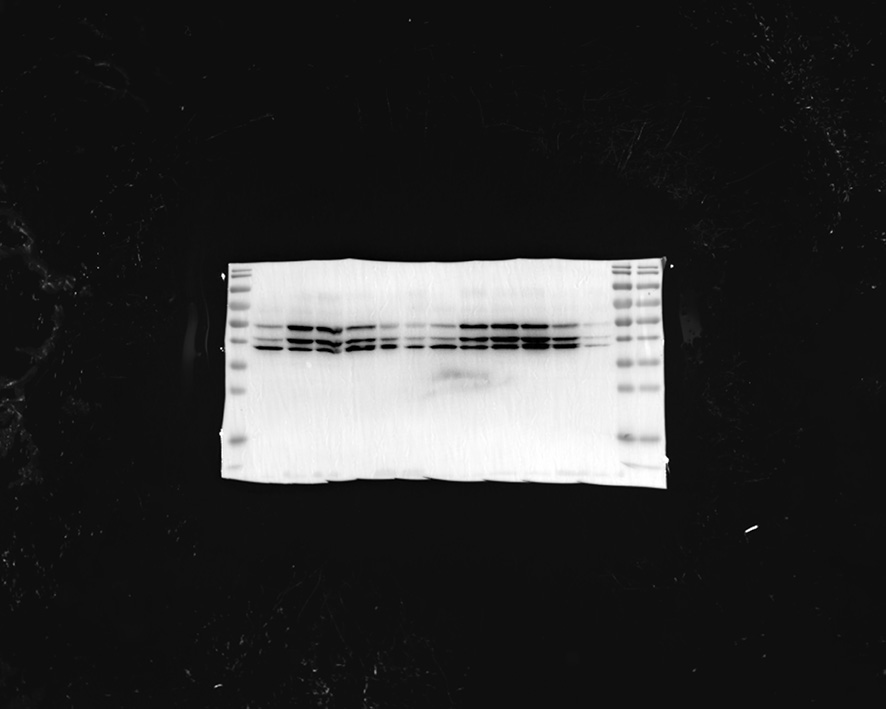

Supplement: Supplementary file 2 [file DataSheet2.ZIP › Figure 4/p-jnk-1.jpg]

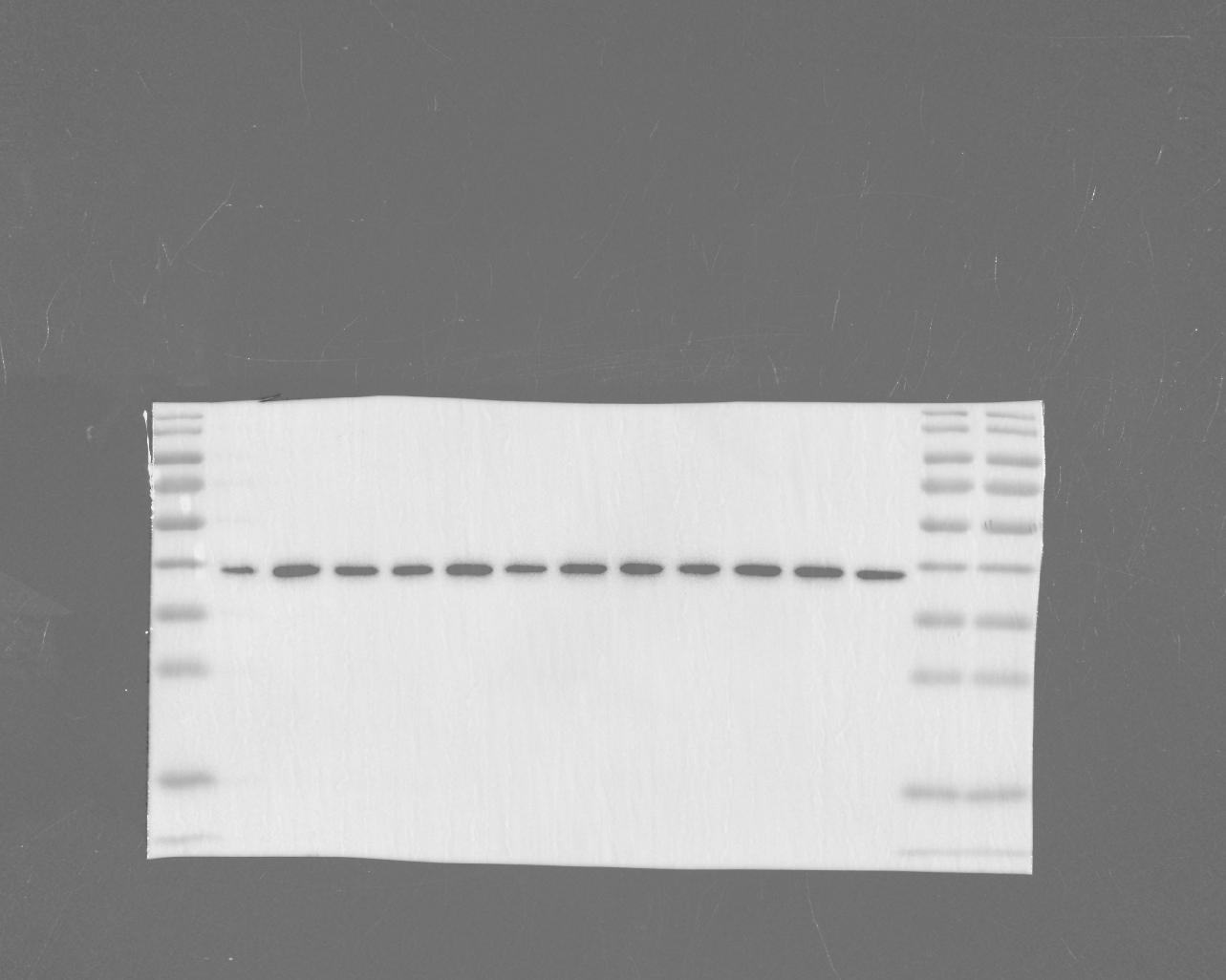

Supplement: Supplementary file 2 [file DataSheet2.ZIP › Figure 4/P38-1.jpg]

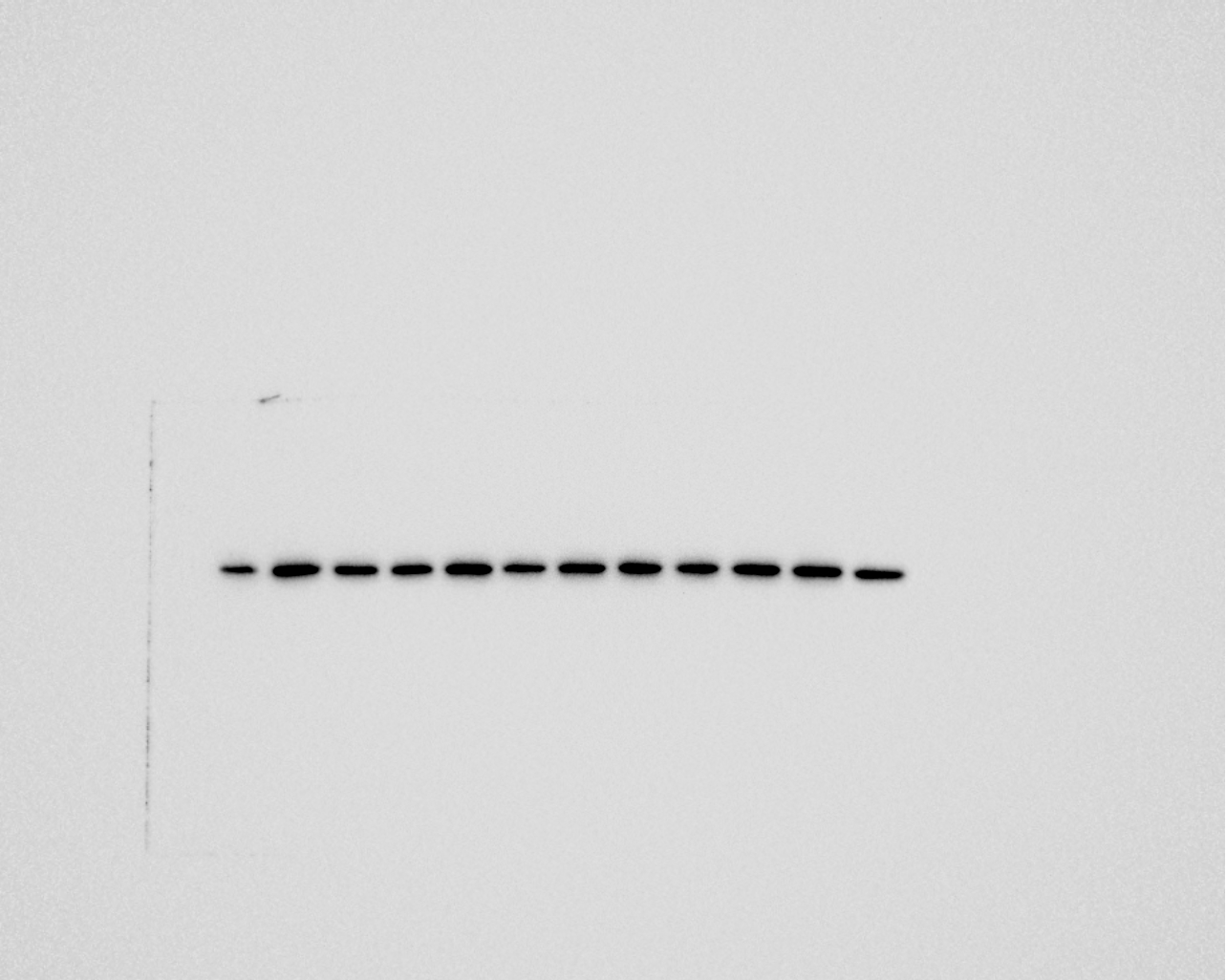

Supplement: Supplementary file 2 [file DataSheet2.ZIP › Figure 4/p38.jpg]

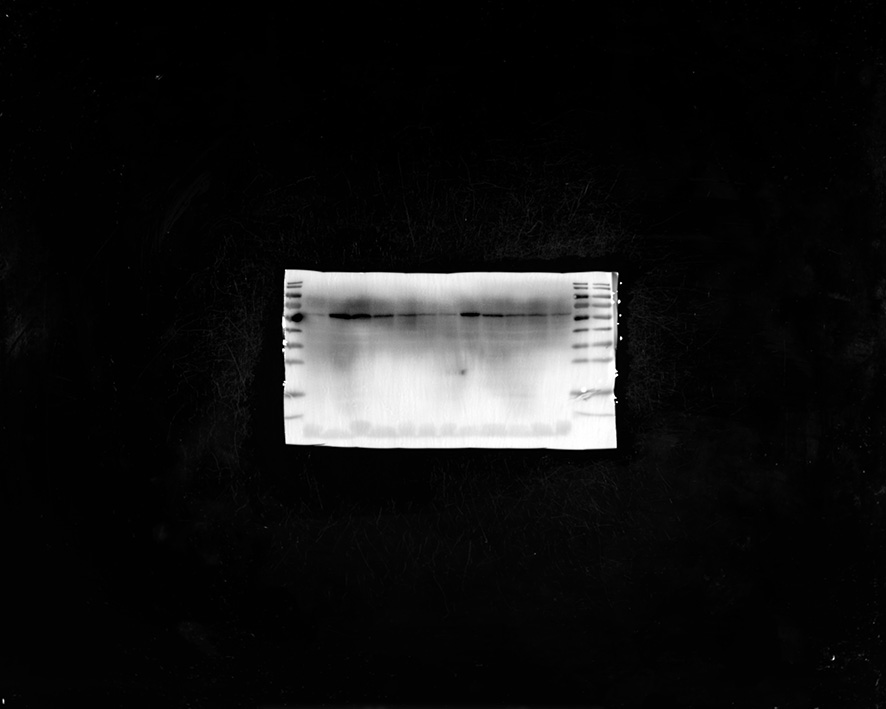

Supplement: Supplementary file 2 [file DataSheet2.ZIP › Figure 4/PAKT-1.jpg]

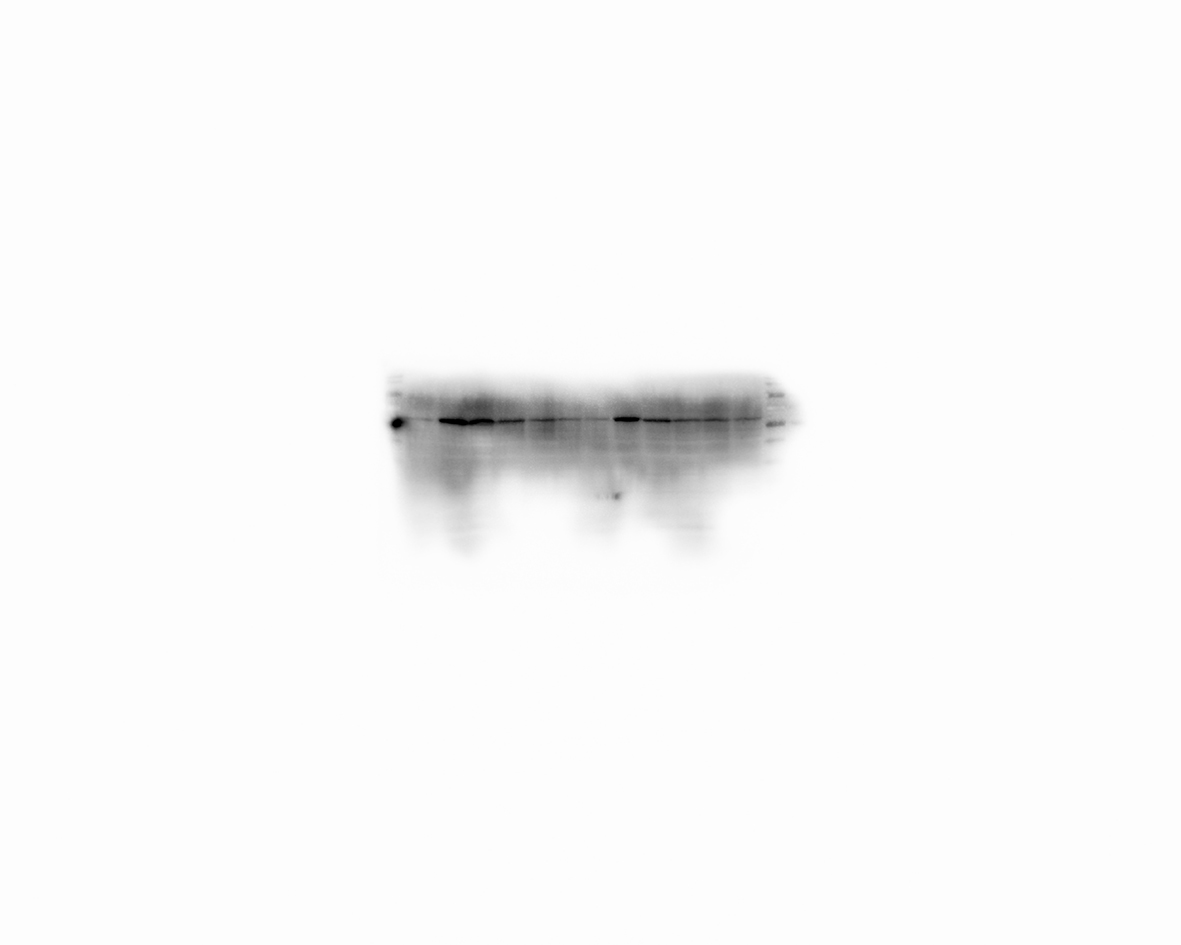

Supplement: Supplementary file 2 [file DataSheet2.ZIP › Figure 4/PAKT.jpg]

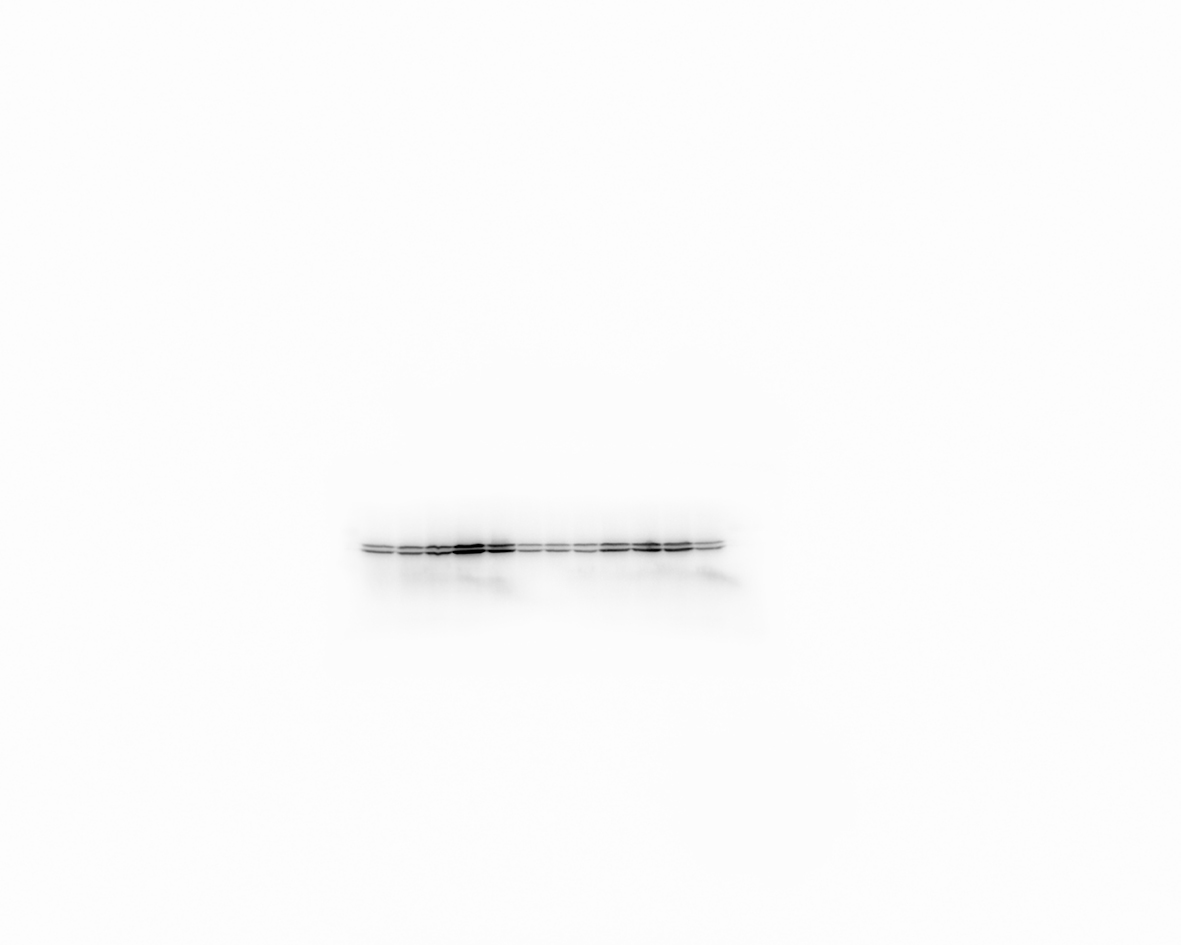

Supplement: Supplementary file 2 [file DataSheet2.ZIP › Figure 4/pERK.jpg]

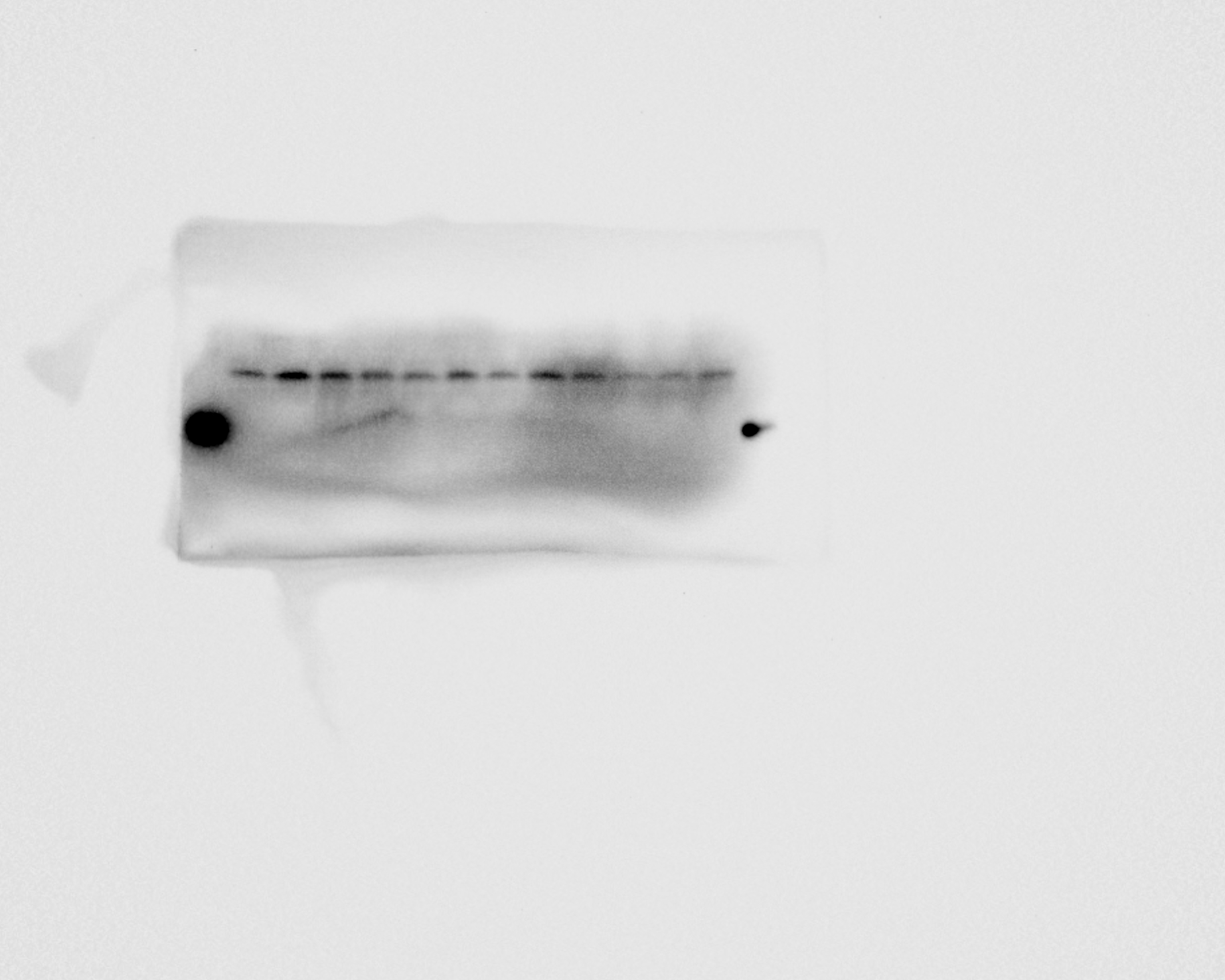

Supplement: Supplementary file 2 [file DataSheet2.ZIP › Figure 4/PIKBA.jpg]

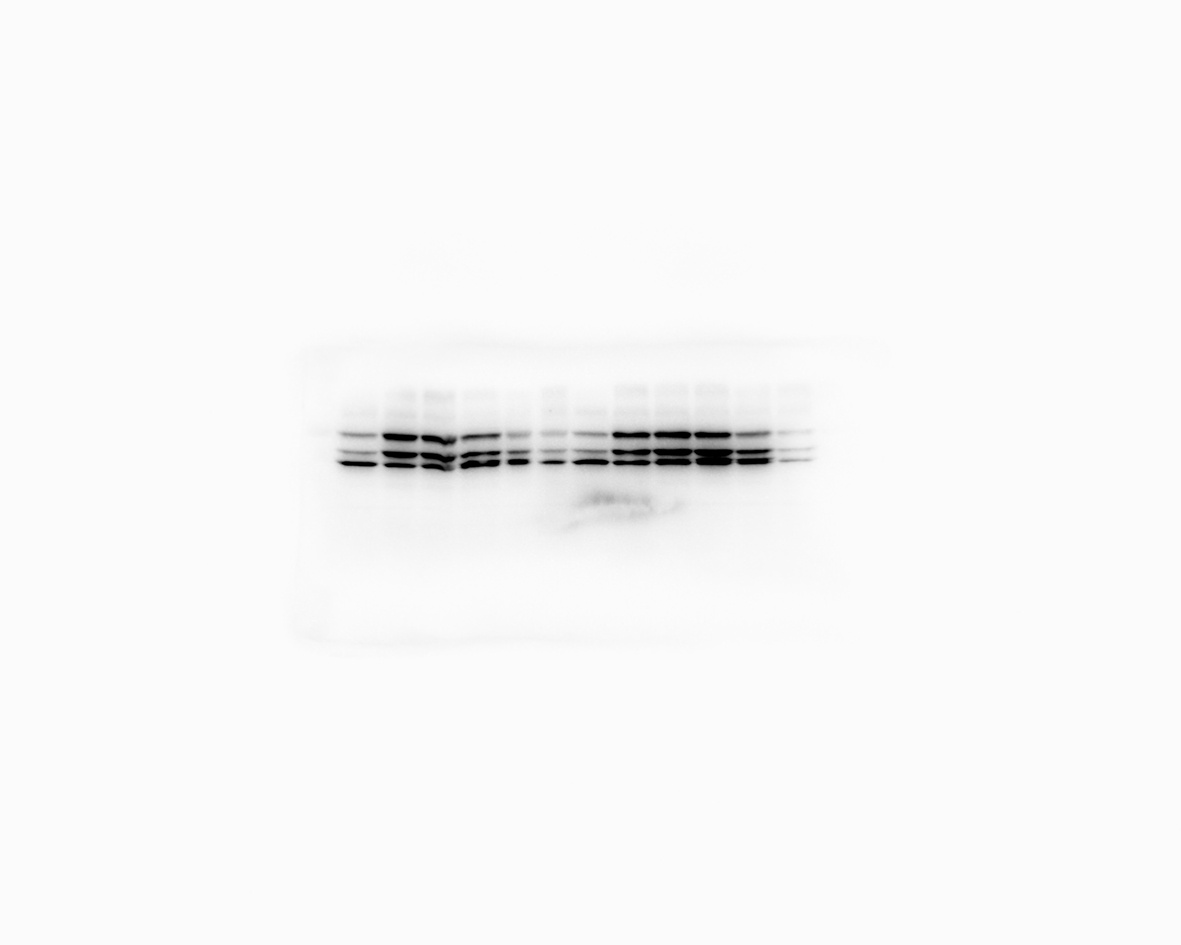

Supplement: Supplementary file 2 [file DataSheet2.ZIP › Figure 4/PJNK.jpg]

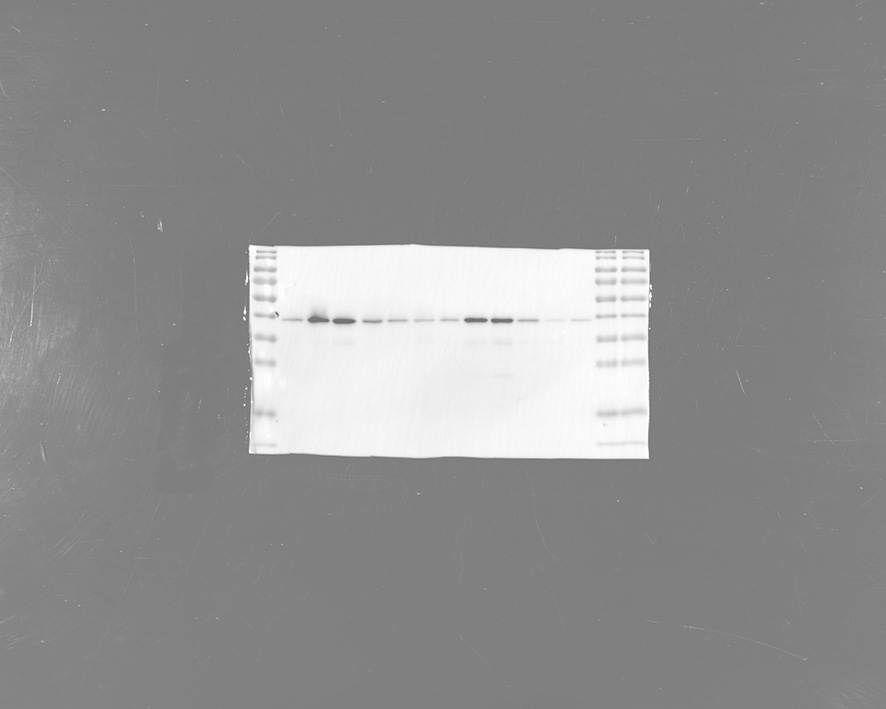

Supplement: Supplementary file 2 [file DataSheet2.ZIP › Figure 4/pp38-1.jpg]

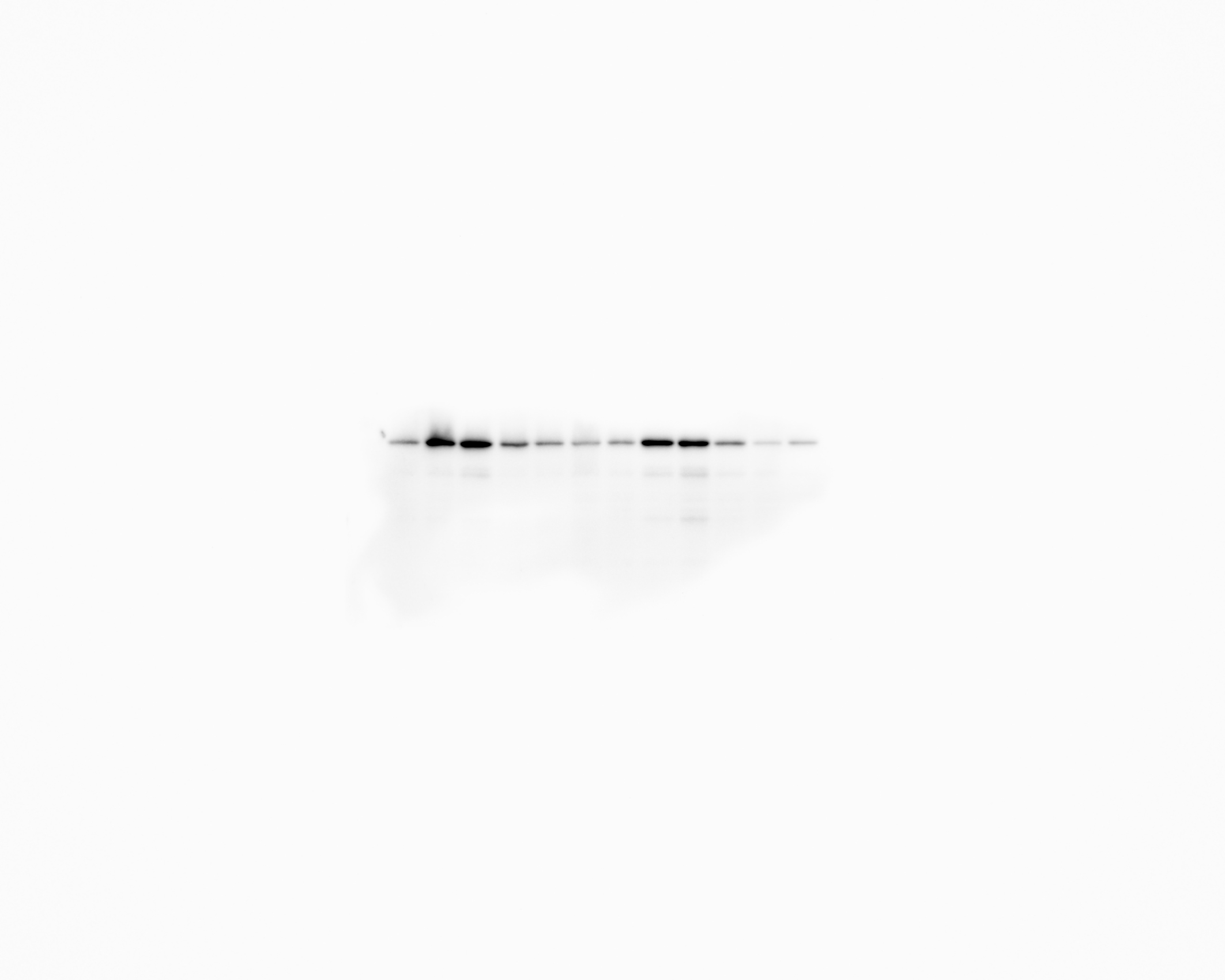

Supplement: Supplementary file 2 [file DataSheet2.ZIP › Figure 4/pp38.jpg]

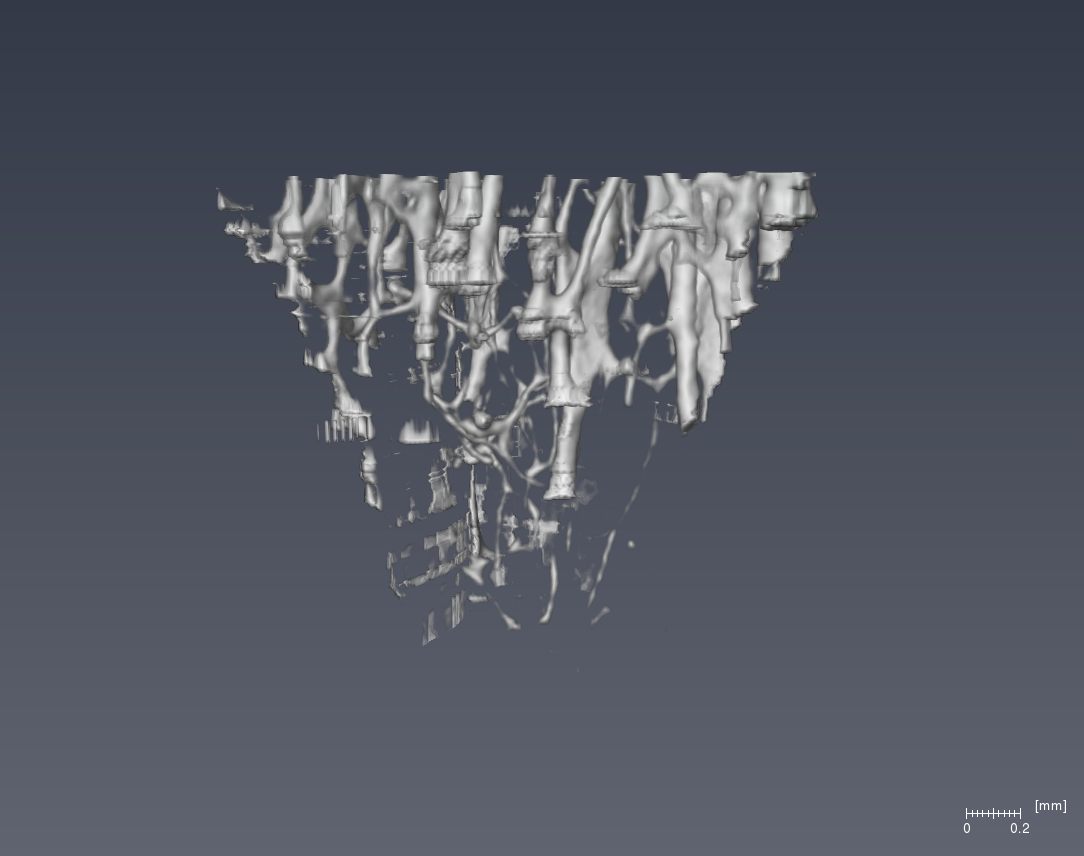

Supplement: Supplementary file 2 [file DataSheet2.ZIP › Figure 5/HD-1.tif]

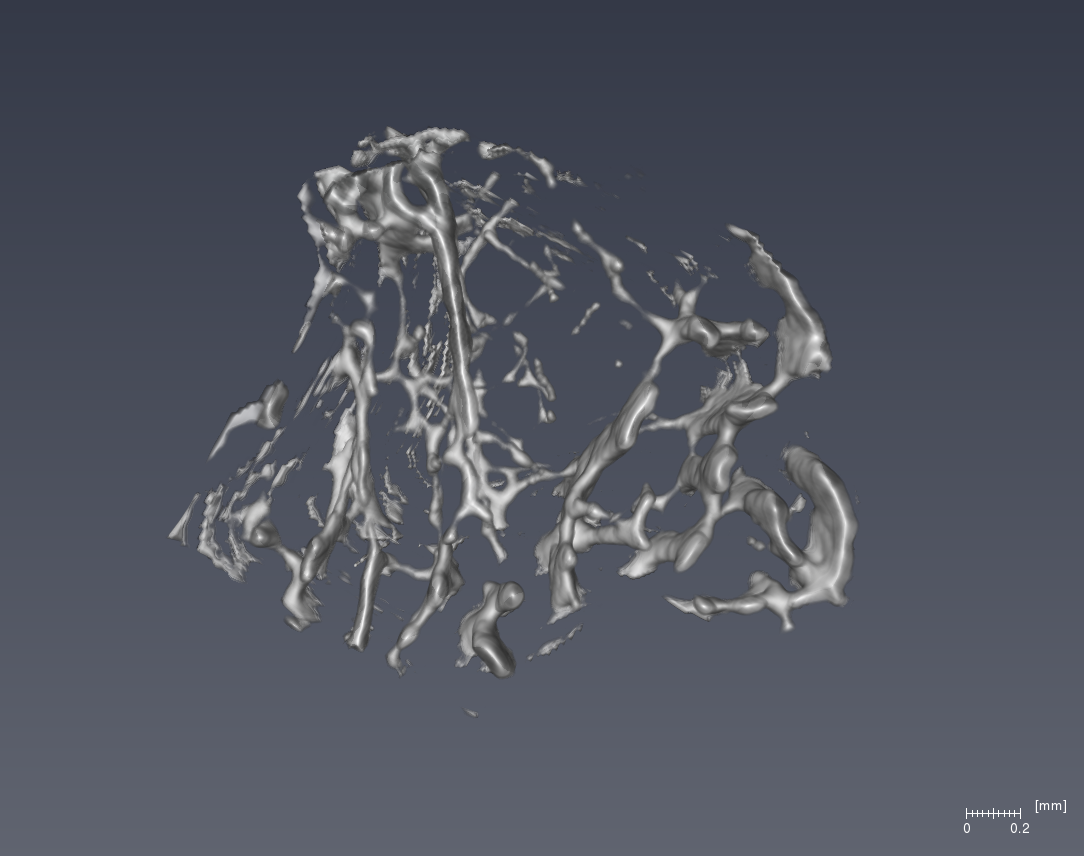

Supplement: Supplementary file 2 [file DataSheet2.ZIP › Figure 5/HD-2.tif]

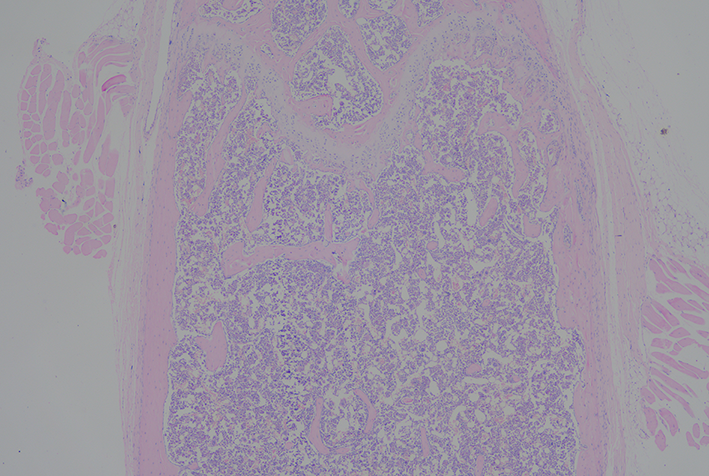

Supplement: Supplementary file 2 [file DataSheet2.ZIP › Figure 5/HD-HE.tif]

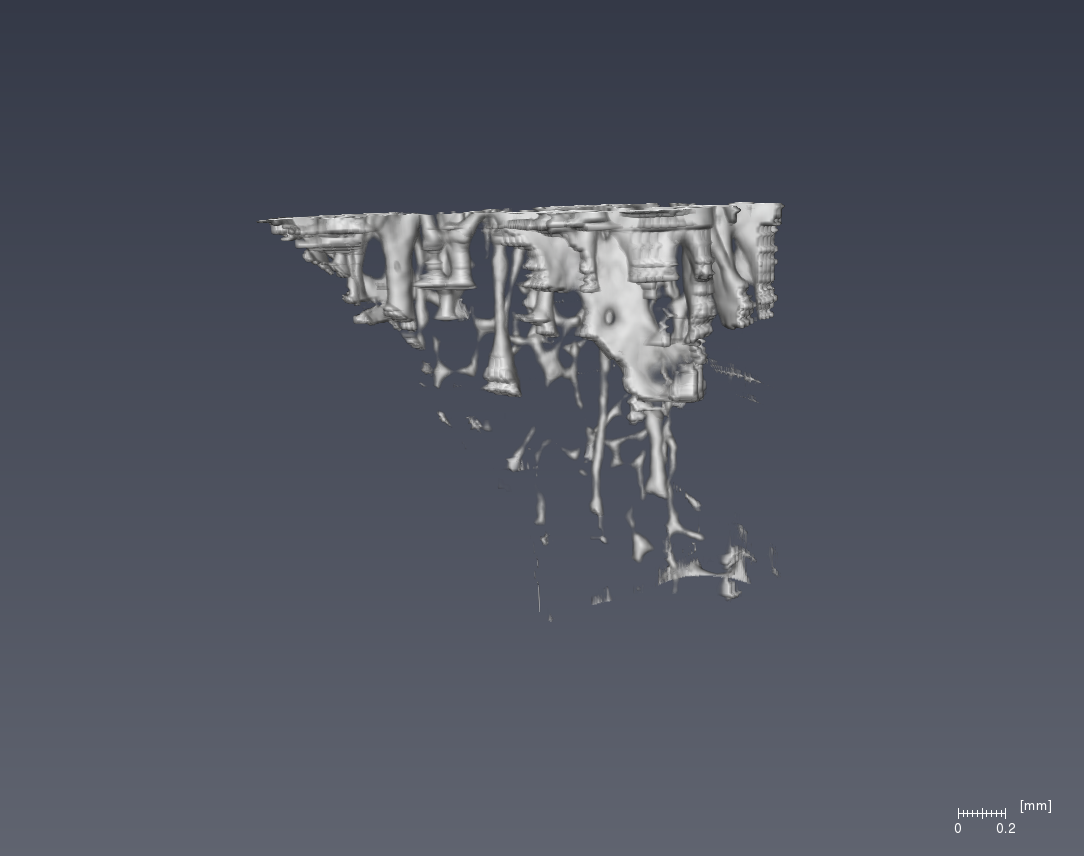

Supplement: Supplementary file 2 [file DataSheet2.ZIP › Figure 5/LD-1.tif]

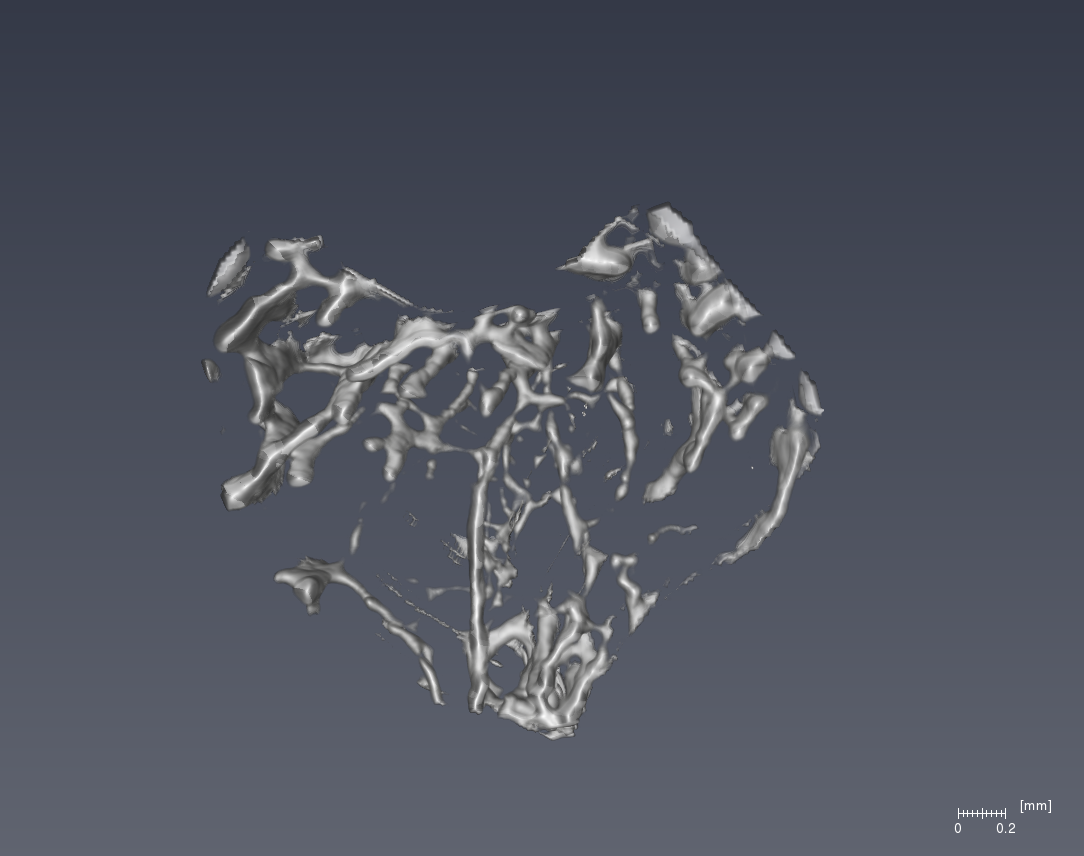

Supplement: Supplementary file 2 [file DataSheet2.ZIP › Figure 5/LD-2.tif]

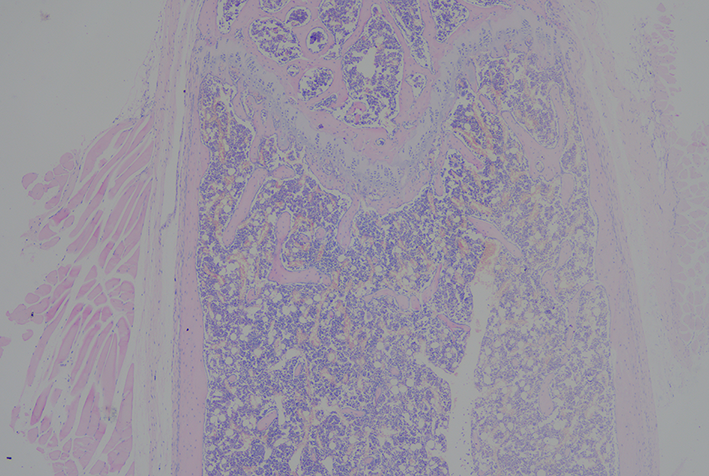

Supplement: Supplementary file 2 [file DataSheet2.ZIP › Figure 5/LD-HE.tif]

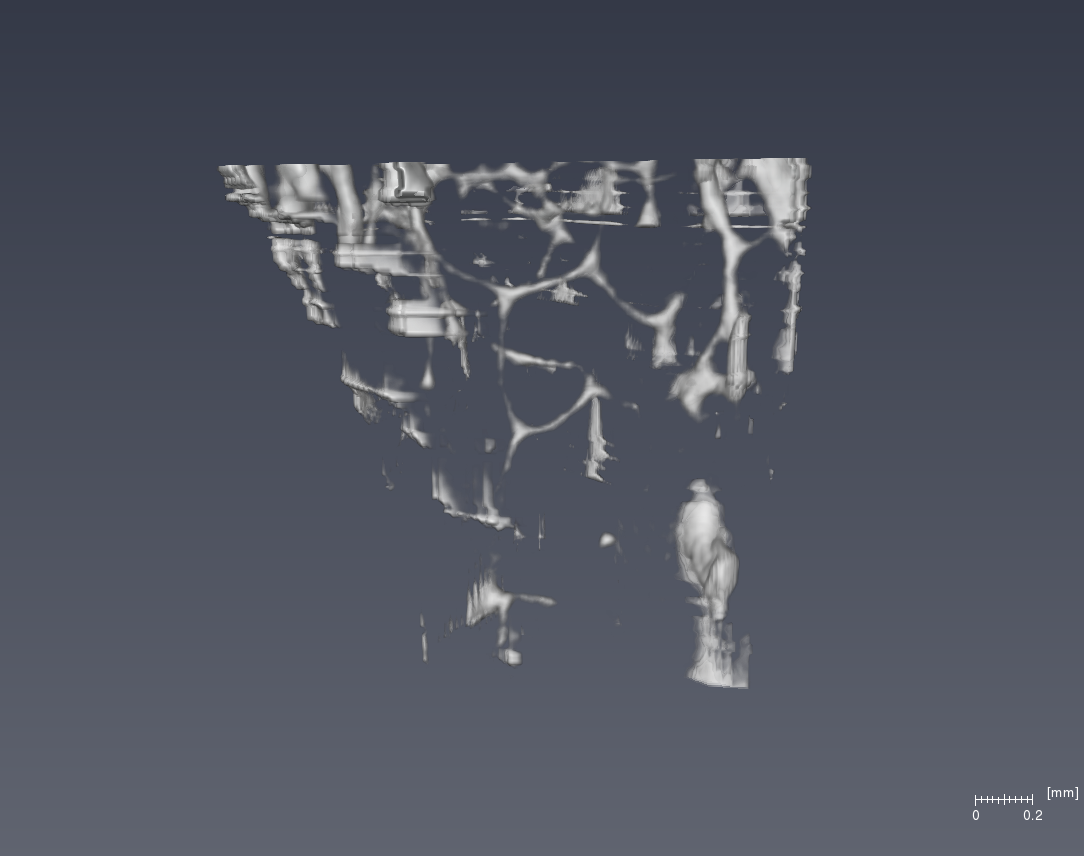

Supplement: Supplementary file 2 [file DataSheet2.ZIP › Figure 5/OVX-1.tif]

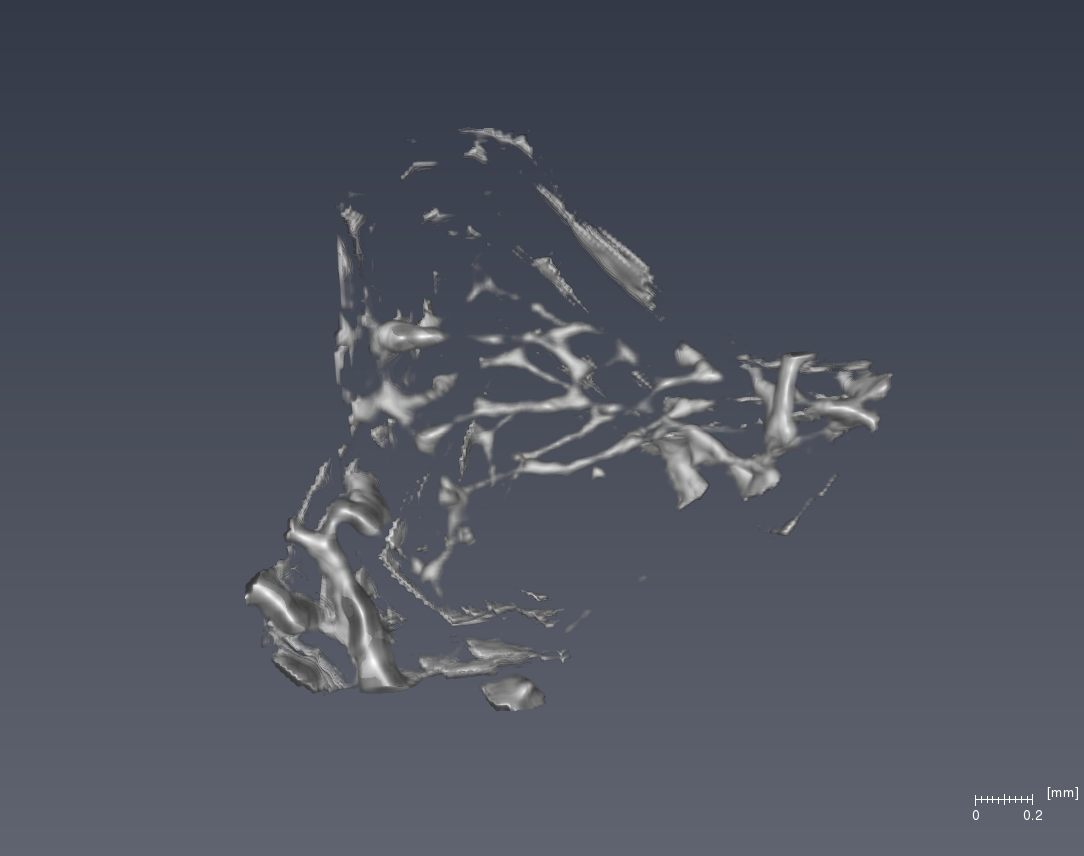

Supplement: Supplementary file 2 [file DataSheet2.ZIP › Figure 5/OVX-2.tif]

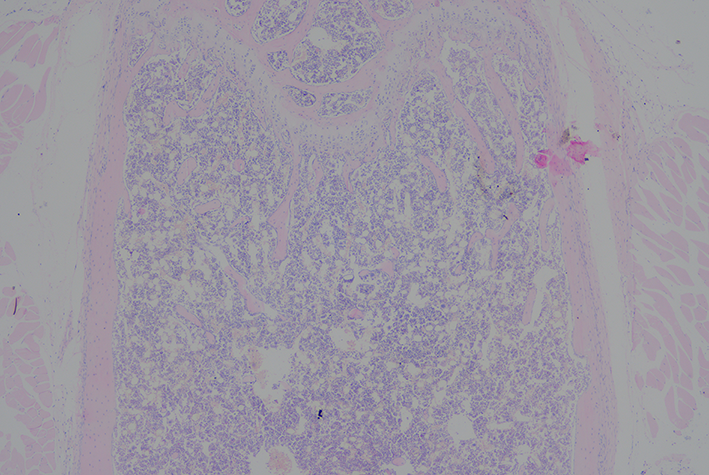

Supplement: Supplementary file 2 [file DataSheet2.ZIP › Figure 5/OVX-HE.tif]

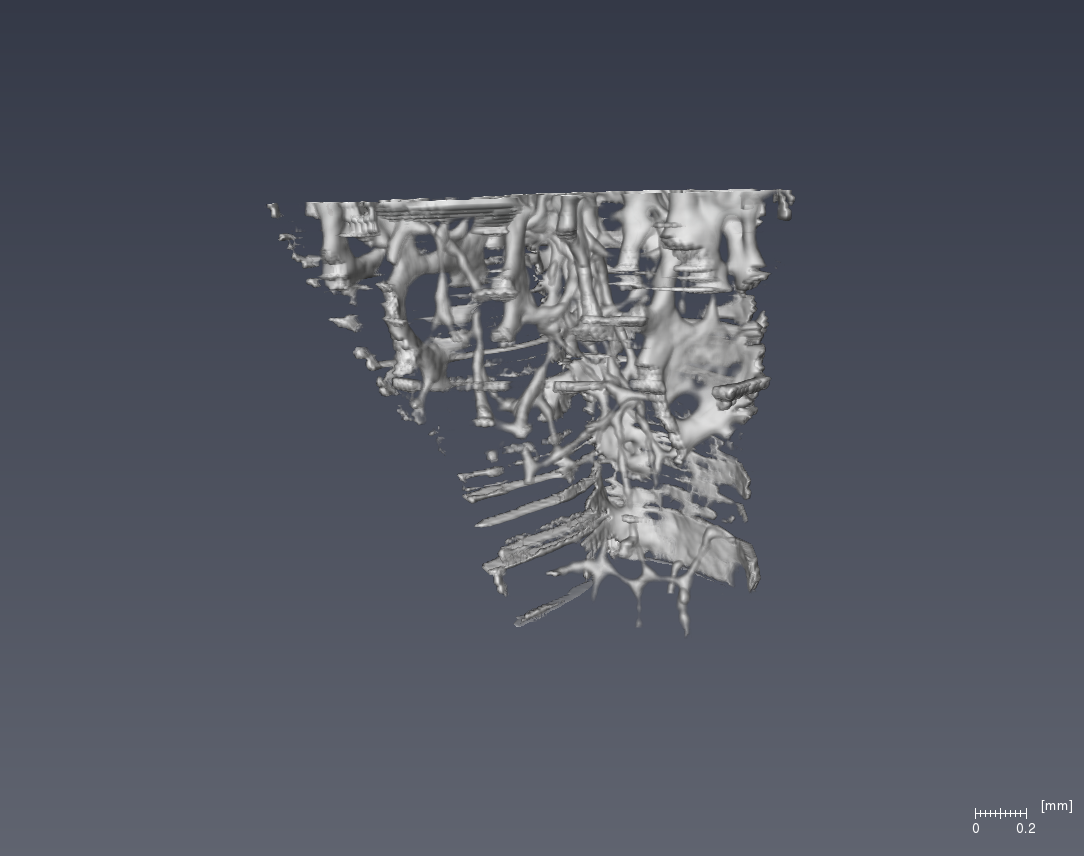

Supplement: Supplementary file 2 [file DataSheet2.ZIP › Figure 5/SHAM-1.tif]

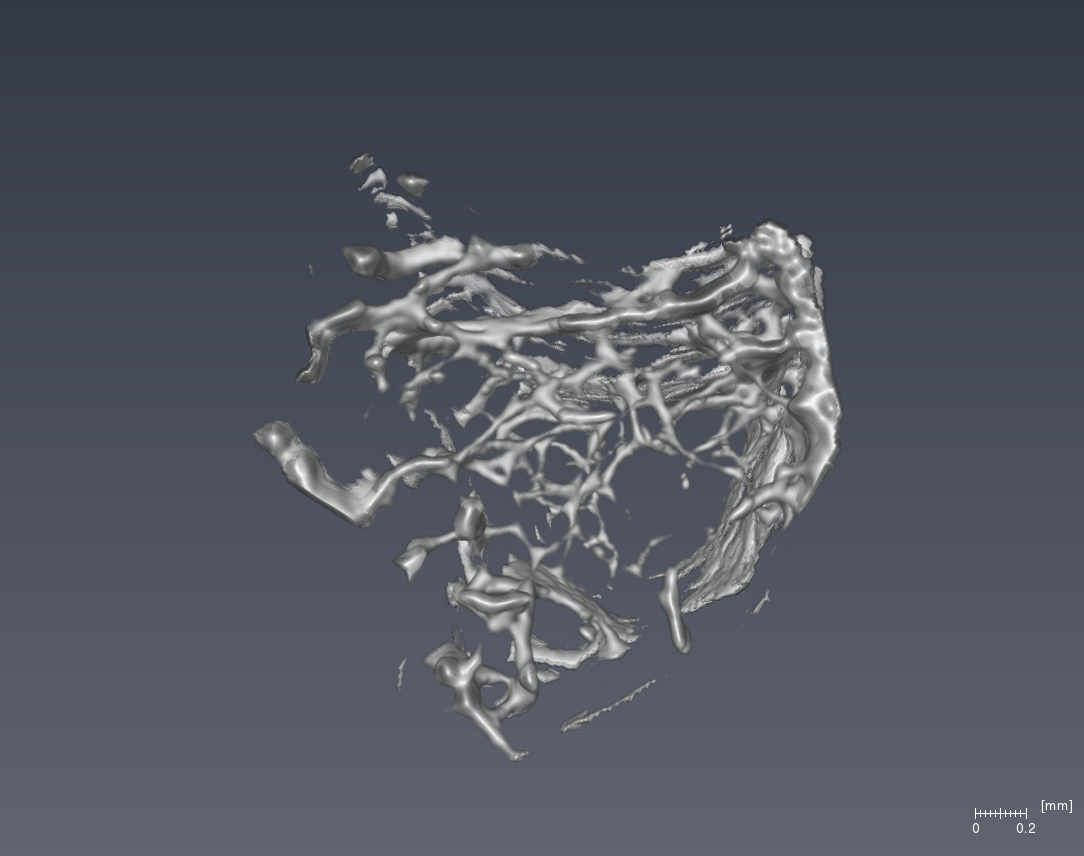

Supplement: Supplementary file 2 [file DataSheet2.ZIP › Figure 5/SHAM-2.tif]

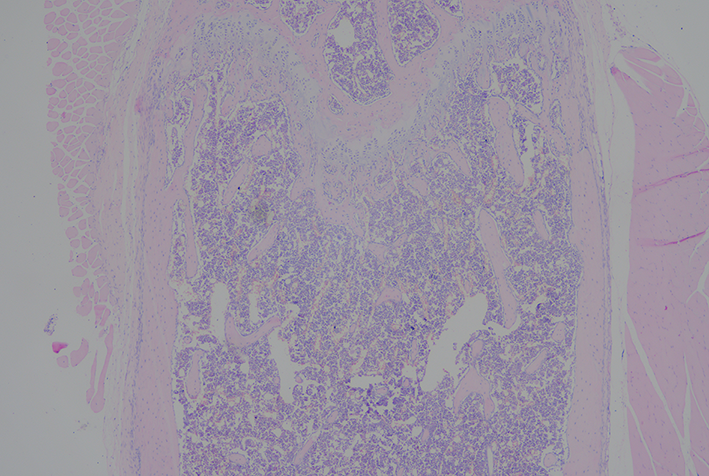

Supplement: Supplementary file 2 [file DataSheet2.ZIP › Figure 5/SHAM=HE.tif]
